# Cotton D genome assemblies built with long-read data unveil mechanisms of centromere evolution and stress tolerance divergence

**DOI:** 10.1186/s12915-021-01041-0

**Published:** 2021-06-03

**Authors:** Zhaoen Yang, Xiaoyang Ge, Weinan Li, Yuying Jin, Lisen Liu, Wei Hu, Fuyan Liu, Yanli Chen, Shaoliang Peng, Fuguang Li

**Affiliations:** 1 Zhengzhou Research Base, State Key Laboratory of Cotton Biology, Zhengzhou University, Zhengzhou, 450001 China; 2 Institute of Cotton Research, Chinese Academy of Agricultural Sciences, Anyang, 455000 China; 3 College of Computer Science and Electronic Engineering, Hunan University, Changsha, 410082 China; 4 School of Computer Science, National University of Defense Technology, Changsha, 410073 China; 5 Peng Cheng Lab, Shenzhen, 518000 China; 6 Biomarker Technologies Corporation, Beijing, 101300 China

**Keywords:** *G. thurberi*, *G. davidsonii*, Salt tolerance, *Verticillium wilt*, Structural variation, Long-range interactions, Hi-C, 3D genome, *Gossypium*

## Abstract

**Background:**

Many of genome features which could help unravel the often complex post-speciation evolution of closely related species are obscured because of their location in chromosomal regions difficult to accurately characterize using standard genome analysis methods, including centromeres and repeat regions.

**Results:**

Here, we analyze the genome evolution and diversification of two recently diverged sister cotton species based on nanopore long-read sequence assemblies and Hi-C 3D genome data. Although D genomes are conserved in gene content, they have diversified in gene order, gene structure, gene family diversification, 3D chromatin structure, long-range regulation, and stress-related traits. Inversions predominate among D genome rearrangements. Our results support roles for 5mC and 6mA in gene activation, and 3D chromatin analysis showed that diversification in proximal-vs-distal regulatory-region interactions shape the regulation of defense-related-gene expression. Using a newly developed method, we accurately positioned cotton centromeres and found that these regions have undergone obviously more rapid evolution relative to chromosome arms. We also discovered a cotton-specific LTR class that clarifies evolutionary trajectories among diverse cotton species and identified genetic networks underlying the *Verticillium* tolerance of *Gossypium thurberi* (e.g., SA signaling) and salt-stress tolerance of *Gossypium davidsonii* (e.g., ethylene biosynthesis). Finally, overexpression of *G. thurberi* genes in upland cotton demonstrated how wild cottons can be exploited for crop improvement.

**Conclusions:**

Our study substantially deepens understanding about how centromeres have developed and evolutionarily impacted the divergence among closely related cotton species and reveals genes and 3D genome structures which can guide basic investigations and applied efforts to improve crops.

**Supplementary Information:**

The online version contains supplementary material available at 10.1186/s12915-021-01041-0.

## Background

Modern cultivated cotton has narrow genetic diversity, a situation which limits the improvement potential of these species [1, 2]. Besides the four cultivated species, there are more than 45 wild cotton species, and these have been grouped into nine genomic types (A–G plus K for diploids; AD for tetraploids) based on their kinship; these wild cotton species represent important resources for cotton breeding and the study of cotton evolution and domestication [3–6]. The diploid D genome type comprises 13 species, distributed from Southwest Mexico to Arizona, with additional disjunct species distributions in Peru and the Galapagos Islands [7].

Even though none of the D diploid species produce commercial fibers, the diploid D genome is known as the donor of the D subgenome in wild and domesticated allotetraploid cotton, and the D genome harbors potentially useful genes for improving fiber quality, disease and pest resistance, and cytoplasmic male sterility, as well as drought and salt tolerance in domesticated cotton [8, 9]. Because of their close relationships to the agronomically important cultivated cotton, the diversity, distribution, phylogenetic relationships, and taxonomy of the D genome wild cotton species have attracted scientific interest [7, 10].

Genomics research about D genomes was substantially advanced by the sequencing and de novo assembly of genomes for *G. raimondii* (D_5_) and *Gossypium turneri* (D_10_), yet genomic information for most D genome species remains unavailable [11, 12]. *G. thurberi* (D_1_) and *G. davidsonii* (D_3_) have the same number of chromosomes and similar content of genes with the closely related *G. raimondii* and *G. turneri* [7]. However, the phylogenetic and genetic data—as well as the plant classifications recognized by early taxonomists—support that *G. thurberi* and *G. davidsonii* are genetically distinct from *G. raimondii* and *G. turneri* [7]. Moreover, they also showed different phenotypic characters: compared with *G. raimondii*, *G. thurberi* is more tolerant to *Verticillium* wilt and *G. davidsonii* is more tolerant to salt.

Functional impacts from centromeres have been appreciated for more than 130 years, and although centromeres have been characterized using both cytological and genetic approaches, elucidating the molecular basis through which centromere exert their functional impacts is a central, ongoing pursuit in molecular biology research [13]. We know that centromeres are functionally conserved across eukaryotes, for example helping to ensure faithful transmission of the genome during cell division, but centromeres are often poorly represented in the genome assemblies. This poor representation reflects the highly repetitive sequences comprising centromeres, which has made them the most technologically challenging genome regions to assemble, particularly when using short-read sequencing data. Indeed, for many species, centromere positions on chromosomes are still determined based on phylogenetic analyses or chromatin immunoprecipitation, methods which are both laborious and indirect [14]. The inability to accurately assemble centromeres in genome assemblies has limited our understanding of the functional mechanisms and evolutionary histories of these highly impactful genomic structures. And the capacity to resolve centromeres has been one of the major application cases for the introduction of long-read sequencing technologies into genomics research.

Here, we report high-quality genomes for *G. thurberi* and *G. davidsonii* that were assembled based on nanopore long reads and high-throughput chromosome conformation capture (Hi-C) technology, thereby substantially improving the quality and utility of the genomic resources available for research in this important cash crop. Using these high-quality genomes, we performed genome-wide comparative studies that revealed the contrasting features among *G. thurberi*, *G. davidsonii*, and *G. raimondii*, including for example chromosomal reconstruction analysis of species divergence, gene family expansion, gene-order and structural variations, methylation features, and long-range interactions between proximal and distal regulatory regions. Some findings from these analyses include the observation that genes with chromosomal interactions have higher expression levels than those without interactions, the finding that the relatively low levels of 5mC and 6mA in A compartments and at TAD boundaries may contribute to the activation of nearby genes, and a demonstration that recent *Gypsy* LTR expansion has driven the substantial divergence in orthologous centromere sequences among these closely related species. We also found that enhanced SA signaling and ethylene biosynthesis contribute to the respective abilities of *G. thurberi* and *G. davidsonii* to cope with biotic (*Verticillium dahliae*) and abiotic (salinity) stress.

## Results

### Genome sequencing, assembly, and annotation

We assembled the genomes of *G. thurberi* and *G. davidsonii* using data from both the nanopore long-read and the Hi-C short-read technologies. We produced 114.3 Gb and 108.3 Gb clean reads, respectively, for *G. thurberi* (~ 146×) and *G. davidsonii* (~ 135×) using the Nanopore platform (Additional file 1: Table S1-S2). After correction using the Illumina short reads, we generated a *G. thurberi* genome of 779.6 Mb with a contig N50 of 24.7 Mb; the corresponding values for *G. davidsonii* were 801.2 Mb and 26.8 Mb (Table 1 and Additional file 1: Table S3); the sequence continuities are significantly improved for both species as compared with other recently reported genome assemblies [15, 16].

**Table 1:** Global statistical comparison of *G. thurberi* and *G. davidsonii* genome

Category	***G. thurberi***					***G. davidsonii***				
	Numbers	N50	Longest	Size	Percentage of assembly	Numbers	N50	Longest	Size	Percentage of assembly
		(Mb)	(Mb)	(Mb)			(Mb)	(Mb)	(Mb)	
Contigs	74	24.7	49.9	779.6	100	104	26.8	47.4	801.2	100
Anchored and oriented	63	24.7	49.9	777.3	99.7	90	26.8	47.4	799.1	99.7
Gene annotated	41,316	NA	NA	111.9	14.4	41,471	NA	NA	113.9	12.5
Repeat sequence	NA	NA	NA	451.8	58.0	NA	NA	NA	469.4	58.6

*NA* not applicable

Using 284 million and 280 million valid Hi-C interaction pairs for the *G. thurberi* and *G. davidsonii* genomes, respectively (Additional file 1: Table S4), we anchored and oriented 777.2 and 799.2 Mb of the assembly onto 13 pseudochromosomes of *G. thurberi* and *G. davidsonii* respectively (Additional file 2: Fig. S1-S2), which represented more than 99.7% of the total assembly, indicating that our new assemblies reached a reference grade for quality. As an indication of the improved contiguity, the contig length for our *G. thurberi* genome represents a 940-fold increase compared to previously published *G. thurberi* sequences (24.7 Mb versus 0.026 Mb) [7], and our *G. thurberi* genome has a 3750-fold reduction in fragmentation (74 versus 277,903). Similarly, there was an 836-fold increase for *G. davidsonii* genome contig length (26.8 Mb versus 0.032 Mb) and 5150-fold reduction in fragmentation (104 versus 535,698). Moreover, the total assembly length and gene annotation number were all higher for our *G. thurberi* and *G. davidsonii* genome assemblies as compared to the recently reported *G. thurberi* and *G. davidsonii* genome resources [7]. Approximately 58.0% and 58.6% of the assembly sequences were annotated as repetitive sequences in the *G. thurberi* and *G. davidsonii* assemblies, respectively (Additional file 1: Table S5).

We next evaluated the assembly completeness by aligning the 192 and 212 million paired-end Illumina short reads against the *G. thurberi* and *G. davidsonii* genome assemblies and BUSCO [17] analysis, both methods showed that both assemblies are of high quality (Additional file 1: Table S6-S7 and Additional file 2: Fig. S3).

### Genomic diversity among six D genomes

Our generation of high-quality genome assemblies for *G. thurberi* and *G. davidsonii* provides an opportunity to compare different D genome species that shared a common ancestor, potentially helping identify the post-divergence genomic rearrangements in cotton. The overall collinearities between the two newly assembly genomes are largely conserved, as supported by more than 78% of *G. thurberi* genome matching in one-to-one syntenic blocks with 80.6 % of the *G. davidsonii* genome. Similarly, we found approximately 78% of the *G. thurberi* genome matched in one-to-one syntenic blocks with ~ 81% of the *G. raimondii* genome (Additional file 1: Table S8). And ~ 77% *G. davidsonii* genome matched in one-to-one syntenic blocks with ~ 83% of the *G. raimondii* genome. Our previous study showed that ~ 86% of the *G. raimondii* genome matched in one-to-one syntenic blocks with the D subgenome of *Gossypium hirsutum* (Gh_D_t1_), confirming that *G. raimondii* is a plausible donor species of allotetraploid cotton species [4].

**Fig. 1.**
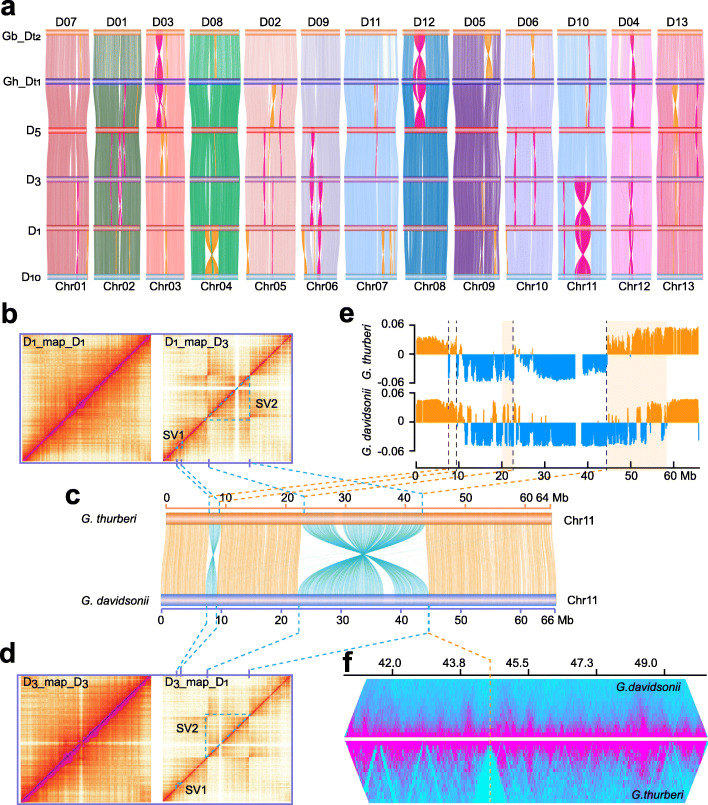
Characterization of genomic variation among different D genomes. **a** Genome comparison of among *G. barbadense* (D subgenome, Gb_D_t2_), *G. hirsutum* (D subgenome, Gh_D_t1_), *G. raimondii* (D_5_), *G. davidsonii* (D_3_), *G. thurberi* (D_1_), and *G. turneri* (D_10_). The inversions are marked in orange and magenta. **b** Identification of a large inversion on Chr11 between *G. thurberi* and *G. davidsonii*. The panel shows chromatin interaction heat maps including *G. thurberi* Hi-C data mapping *G. thurberi* (D_1__map_D_1_) and *G. davidsonii* Hi-C data mapping *G. thurberi* (D_1__map_ D_3_). The triangle marks the inversions in the heat maps. **c** Genomic comparison between *G. thurberi* and *G. davidsonii* on Chr11. **d** The panel shows chromatin interaction heat maps including *G. davidsonii* Hi-C data mapping *G. davidsonii* (D_3__map_ D_3_) and *G. thurberi* Hi-C data mapping *G. davidsonii* (D_1__map_D_3_). The triangle marks the inversions in the heat maps. **e** A/B compartments in Chr11; orange represents the A compartments and blue represents the B compartments. The transparent boxes indicate A-B compartment switching regions. **f** TAD heatmap around the right breakpoint of the large inversion on Chr11

We found inversions are the major rearrangement type among the different D genomes. The inversions between the two new assemblies span approximately 59.6 Mb in *G. thurberi*, a level similar to a previously reported comparison between *G. raimondii* and the TM-1 D subgenome [4]. Of particular note, we detected a large inversion on Chr11 between the *G. thurberi* and *G. davidsonii* occupying 20.4 Mb; note, this was confirmed by mapping the Hi-C data for one accession against to the genome of the other, and *vice versa* (Fig. 1a-d and Additional file 2: Fig. S4-S5). Enlargements from the heatmaps revealed discontinuous signals for these inversions (in the region marked by the color triangle in Fig. 1b).

Our finding that *G. davidsonii*, *G. turneri*, and *G. raimondii* share a conserved syntenic relationship for the large Chr11 inverted region supports that this Chr11 inversion is specific to *G. thurberi*. Further, we detected that *G. thurberi* Chr11 exhibits extensive B-to-A compartment switching specifically in a region neighbor the right breakpoint of the large inversion (Fig. 1e). And we also found that, relative to *G. davidsonii*, the topologically associating domains (TAD) were obviously extensively reorganized near the breakpoints of *G. thurberi* Chr11 breakpoints (Fig. 1f). We analyzed the conserved and switched A-B compartments in the inverted regions and at the whole-genome level. We found 39 conserved and 26 switched A-B compartments in the inverted regions, and the corresponding values for the whole genomes were 1045 and 532. Chi-square tests suggested that there was no bias towards A-B compartment switching in inverted regions (chi-square test, *P* = 0.2664). In contrast, we detected and obviously elevated proportion of reorganized TAD boundaries near the breakpoints (70 out of 190) when compared to the whole genome (1143 out 6184) (chi-square test, *P* < 0.0001) (Additional file 2: Fig. S6). These results offer empirical demonstrations showing that inversions in plant genomes can—in addition to their better understood impacts on one-dimensional linear genome sequences divergence—also drive divergence in TAD boundary formation*.*

In addition to Chr11, we also found some inversions from Chr01, Chr05, Chr06, and Chr12 that are specific to *G. thurberi* because they are shared by *G. davidsonii* and *G. turneri* (Fig. 1a). Similarly, some inversions from *G. davidsonii* include inversions from Chr02, Chr05, Chr06, Chr10, and Chr13, which are shared by *G. raimondii* and *G. thurberi*. Furthermore, we observed that in *G. turneri* (D_10_), *G. hirsutum*, and *Gossypium barbadense* (Gb_D_t2_), most of inversions are species specific (Fig. 1a), indicating that such inversions have formed during species divergence; such structural rearrangements could have directly contributed genetic novelty that contributed to such divergence.

### Genomic landscapes of *G. thurberi* and *G. davidsonii*

**Fig. 2.**
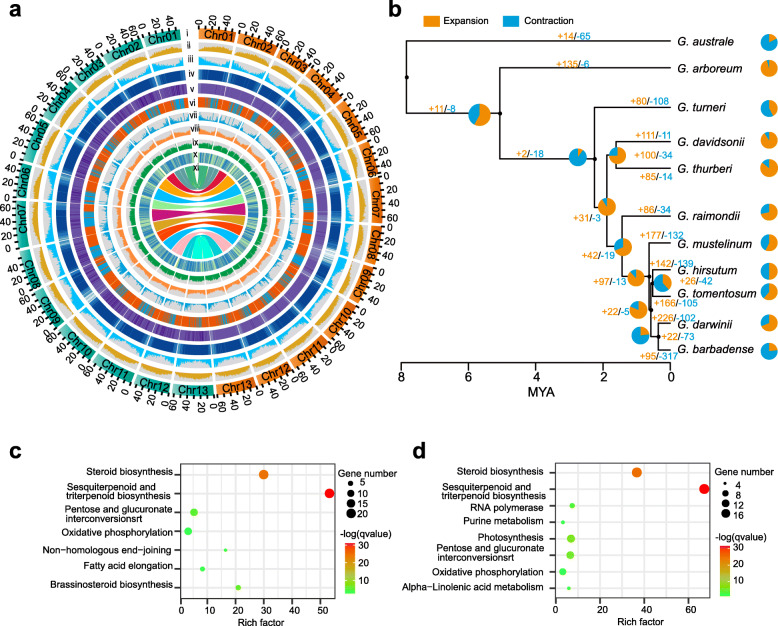
Gene family expansion among 11 cotton species. **a** Genomic landscape between *G. thurberi* and *G. davidsonii* genomes. (i) Genomes of *G. thurberi* (right panel) and *G. davidsonii* (left panel). (ii,iii) Transposable elements and gene density. (iv) 5mC DNA methylation levels. (v) 6mA DNA methylation levels. (vi) A and B compartments across the chromosome, orange indicates A compartments and blue indicates B compartments. (vii) Expression level based on RNA-seq analysis of leaves. The expression level was normalized by the number of reads per bin/(number of mapped read (in millions))×bin length (kb). (viii) InDel density between *G. thurberi* and *G. davidsonii*. (ix) SNP density between *G. thurberi* and *G. davidsonii.* (x) PAV density between *G. thurberi* and *G. davidsonii*. (xi) Syntenic block between *G. thurberi* and *G. davidsonii*. All data in panel (i)-(x) are shown in 500-kb windows. **b** A phylogenetic tree based on 7561 single-copy genes. The ratios of gene expansion and contraction of each branch are showed in the pie diagrams. The digits present the number of gene families which have experienced expansion or contractions. **c**,**d** KEGG pathway enrichment of the gene families which have experienced expansion or contraction in *G. thurberi* and *G. davidsonii*

As with most genomes, the *G. thurberi* and *G. davidsonii* sequences positioned near the telomere are enriched of coding genes while having a lower-than-average level of repeat sequences (Fig. 2a). Again as expected, the pericentromeric regions are enriched for repeat sequences but show a deficit for coding genes compared to the genome-wide average (Fig. 2a).

Our RNA-seq (coding and non-coding) expression profiling of *G. thurberi* and *G. davidsonii* (young leaves) showed that sequences in pericentromeric regions are expressed at generally lower levels compared to sequences in chromosome arms (Fig. 2a). We next examined small variations (InDels and SNPs) between *G. thurberi* and *G. davidsonii*. The InDel densities showed a decreasing pattern, and the SNP densities showed an increasing tendency moving from the telomere region to the centromere region (Fig. 2a and Additional file 2: Fig. S7).

We next detected presence/absence variations (PAVs) and identified a total of 14,401 of *G. thurberi*-specific genomic PAVs and 15,684 of *G. davidsonii*-specific genomic PAVs, occupying 39.5 Mb and 52.0 Mb in *G. thurberi* and *G. davidsonii* genomes. The PAVs are evenly distributed across the chromosomes, with most of the PAVs being shorter than 10 kb (Fig. 2a and Additional file 2: Fig. S8a).

A total of 490 and 570 PAV-localized genes were identified as *G. thurberi*- or *G. davidsonii*-specific genes. Approximately 39.6% and 37.4% of the PAV genes from *G. thurberi* and *G. davidsonii* had apparent orthologs from at least one of the three other *Gossypium* species, underscoring that a relatively small proportion of these PAV genes were present in the ancestral genome (Additional file 2: Fig. S8b). PAV genes without obvious orthologs in the examined *Gossypium* species are likely to have arisen during the divergence and may represent sources of impactful genes that have contributed to the speciation and presently adapted characteristics of *G. thurberi* and *G. davidsonii*.

### Evolution within and between eleven cotton genomes

We also compared new coding genes from the new assemblies with *Gossypium arboreum* (A_2_), *Gossypium australe* (G_2_), *G. raimondii* (D_5_), *G. turneri* (D_10_), and the D subgenomes of the five allotetraploid cotton species (*G. hirsutum*, *G. barbadense*, *Gossypium tomentosum*, *Gossypium mustelinum*, and *Gossypium darwinii*). Our phylogenetic tree supports a monophyletic origin for the allotetraploid species that was likely derived from a hybridization between *G. raimondii* and an A genome species (Fig. 2b). A total of 35,454 orthologous groups were identified through orthoMCL, and as expected, the *G. australe* (G_2_) and *G. arboreum* (A_2_) has more unique genes than those of D genome species, because the genomic divergences are more significant in diverse chromosomal groups than within a single group (Additional file 2: Fig. S9a). GO analysis revealed enrichment for “DNA recombination,” “DNA integration,” and “DNA metabolic process” among the unique gene sets for *G. thurberi* and *G. davidsonii* (Additional file 2: Fig. S9b-c).

Using *G. arboreum* and four D genome species (*G. thurberi*, *G. davidsonii*, *G. raimondii*, and *G. turner*i), we evaluated the divergence times between the diploid A genome and four D genome species and found they apparently diverged between 5.07 and 5.13 MYA, and the four D genomes diverged between 1.51 and 2.04 MYA (Additional file 2: Fig. S10). Within the D genome clade, the greatest extents of divergence were detected between *G. turneri* and the other 3 species, then the followed divergence was between *G. raimondii* and the other 3 species, and the most recent divergence was between *G. thurberi* and *G. davidsonii* (Fig. 2a).

We next used CAFE (Computational Analysis of gene Family Evolution) to estimate gene family expansions and contractions among the 23,825 ortholog groups, which revealed that 8 out of the 11 tested species have experienced more gene family expansions than gene family contractions (*P* < 0.05) (Fig. 2b). This is informative when considered against gene family dynamics known for the D subgenomes of allotetraploid cottons: our finding that a relatively higher proportion of species-specific gene families have experienced expansion or contractions in diploid D genome species compared with gene family dynamics in D subgenomes support that this form of genome divergence is less active in the D subgenomes than in the D genome species (Additional file 2: Fig. S11).

We detected that *G. thurberi* has experienced expansion for genes related to steroid biosynthesis and brassinosteroid biosynthesis, as well as for genes encoding pectinesterase enzymes (Additional file 2: Fig. S12). Given the reported roles of these biochemical pathways and enzymes in diverse stress tolerance responses, perhaps such expansion has contributed to the previously reported *Verticillium dahliae* resistance of *G. thurberi* [18]*.* Enriched genes specific to *G. davidsonii* included genes which function in photosynthesis and oxidative phosphorylation pathways, in the photosystem I reaction center (PsaB), and in the photosystem II reaction center (psbD and psbE) are enriched in *G. davidsonii* (Additional file 2: Fig. S13), results clearly suggesting that the potential for differential photosynthetic capacities in *G. davidsonii.*

### Epigenetic modifications variations in 3D structure

Both PacBio and Nanopore can distinguish modified bases from standard nucleotide bases in plants [19, 20]. However, the accuracy of SMRT sequencing for detecting DNA methylation is known to be heavily affected by the sequence coverage [21, 22]. We used the nanopore data to analyze the global landscape of epigenetic modifications on chromosomes. The global N6-methyldeoxyadenine (6mA) level is approximate 1.1% of all adenines for *G. thurberi* and 1.3% for *G. davidsonii,* these proportions are much higher than previous reports about *G. hirsutum* and *G. barbadense* that were based on PacBio sequencing data [19]. For both *G. thurberi* and *G. davidsonii*, the 6mA distribution is uneven across the chromosomes, for example exhibiting enrichment at both the middle regions of chromosome arms and in pericentromeric regions (Fig. 2a), findings supporting the proposal from a rice study that the genomic distribution of 6mA is not random [23]. A comparison of the *G. davidsonii* genome methylation frequencies generated using Nanopore technology with the methylation frequencies obtained through whole-genome bisulfite sequencing technology showed an excellent correlation between the two methods (*R* = 0.88). Among the three types of methylation (CHG, CG, and CHH), CHG showed the highest correlation (0.95), followed by CG (0.92) and CHH (R = 0.77) (Additional file 2: Fig. S14).

The chromosome can be experimentally delineated into open (A) or closed (B) compartments, and these A/B compartments can be further divided into smaller TADs. We found that A compartments tended to cluster at chromosome arms, while B compartments tended cluster near pericentromeric regions (Fig. 2a). Approximately 41.5% of the *G. thurberi* genome belongs to A compartments; this was 42.3% for *G. davidsonii* and 42.0% for *G. raimondii* genome. Note that these A/B compartment ratios are similar with ratios previously reported for allotetraploid D subgenomes [24].

**Fig. 3.**
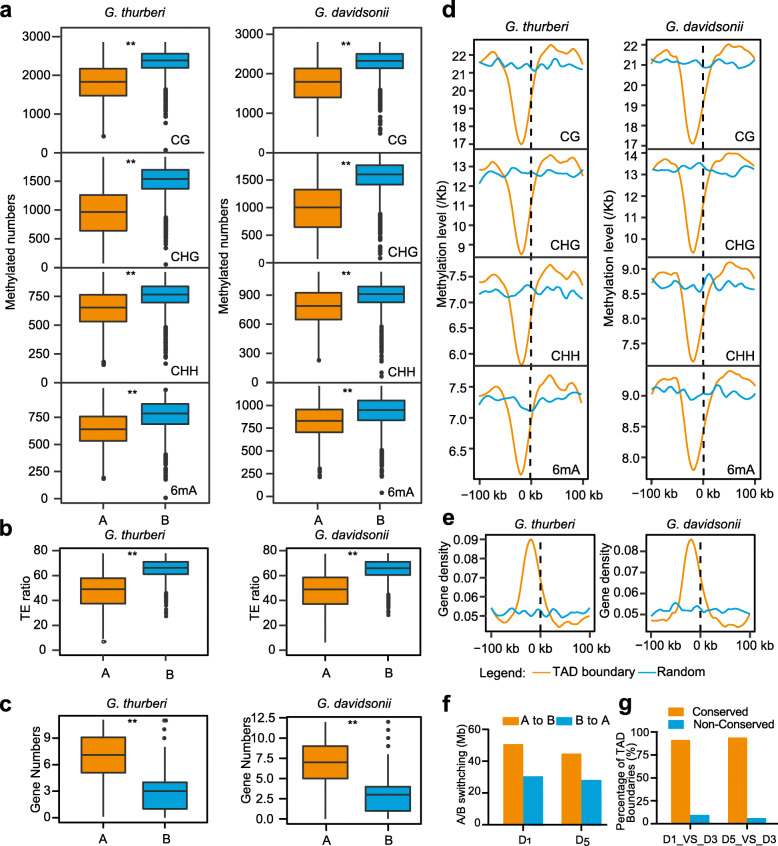
Methylation features of 3D chromatin. **a–c** Methylation level, TE ratio, and gene density in A and B compartments in *G. thurberi* and *G. davidsonii*. A two-sided Wilcoxon signed-rank indicates there were significant differences at **P < 0.001. **d** Methylation feature around TAD boundaries. The methylation levels in TAD boundaries (orange lines) flanking 100 kb was compared with those methylation levels in random genome regions (blue lines). The lines on the right side (0 to 100 kb) indicate TAD regions, and the lines on the left side (− 100 to 0 kb) indicate TAD regions when TADs were organized consecutively or non-TAD regions when one TAD was not closely adjacent to the others. **e** Gene distribution around the TAD boundaries. The method for extracting genomic regions around boundaries was the same as that in panel **d**. **f** A-B compartment switching between *G. thurberi* (D_1_) and *G. davidsonii* (D_3_) or between *G. raimondii* (D_5_) and *G. davidsonii* (D_3_). **g** Comparison of TAD boundaries between *G. thurberi* and *G. davidsonii* (D_1__Vs_D_3_) or *G. raimondii* and *G. davidsonii* (D_5__Vs_D_3_)

We further evaluated the epigenetic features in the A/B compartments for *G. thurberi* and *G. davidsonii* in an analysis using 100-kb windows. For both the *G. thurberi* and *G. davidsonii* genomes, the gene densities were much higher in the A compartments than the B compartments (Fig. 3c). Further, it was intriguing to observe that the levels of both 5mC (CG, CHH, and CHG) and 6mA were significantly lower in A compartments than in B compartments (Fig. 3a). Similarly, the TE content was much lower in A compartments as compared to B compartments (Fig. 3b).

We also analyzed epigenetic modifications around TAD boundaries and found that chromatin surrounding the TAD boundaries in both of the examined cotton species had relatively lower levels of 5mC (CG, CHG, and CHH) and 6mA compared against randomly sampled genomic regions (Fig. 3d). Notably, there is enrichment for ORF sequences at TAD boundaries, suggesting that epigenetic modifications may apparently contribute to the differential activation of genes positioned at TAD boundaries.

To check for higher-order structural variations possibly related to the divergence of D genome species, we compared 3D structures among *G. thurberi*, *G. davidsonii*, and *G. raimondii*. Specifically, we constructed chromatin interaction maps for *G. thurberi*, *G. davidsonii*, and *G. raimondii* at 50 kb resolution, and as expected, the frequency of intra-chromosomal interactions displayed a rapid decrease with extended linear distance (Additional file 2: Fig. S15-S17). This analysis revealed strong rewiring of chromatin interactions in the inverted regions, consistent with a model of distinct territories formed by individual chromosome arms (Additional file 2: Fig. S18). For instance, *G. thurberi* carrying a pericentromeric inversion on Chr11 showed preferential interactions between these loci when present on the same chromosome arm (Additional file 2: Fig. S18).

We compare the organization of A/B compartments between the *G. thurberi* and *G. davidsonii* or between *G. davidsonii* and *G. raimondii*. A total of 57.8 Mb and 44.7 Mb in the *G. thurberi* and *G. raimondii* genomes represented apparent A-to-B compartment switching as compared with the compartment status data for *G. davidsonii* (Fig. 3f). Similarly, a total of 28.9 Mb and 28.1 Mb of genome regions apparently represent B-to-A compartment switching between the *G. thurberi* and *G. raimondii* genomes (Fig. 3f), findings highlighting that B-to-A switching and A-to-B switching are uneven among the diploid D genomes. We also checked the potential for differential expression of genes located in the A/B switching regions: among 3189 A/B switching genes between *G. thurberi* and *G. davidsonii*, 556 were DEGs. Among 3670 A/B switching genes between *G. raimondii* and *G. davidsonii*, 613 were DEGs. These findings support the previous idea that only a small subset of genes are transcriptionally affected by compartment changes [25].

We next compared the TAD boundaries and found that more than 90% of the *G. thurberi* and *G. raimondii* TAD boundaries were conserved in *G. davidsonii* (Fig. 3g), indicating that the TAD boundaries have been relatively strongly conserved among sister species after divergence.

### Long-range interactions in *G. thurberi* and in *G. davidsonii*

**Fig. 4.**
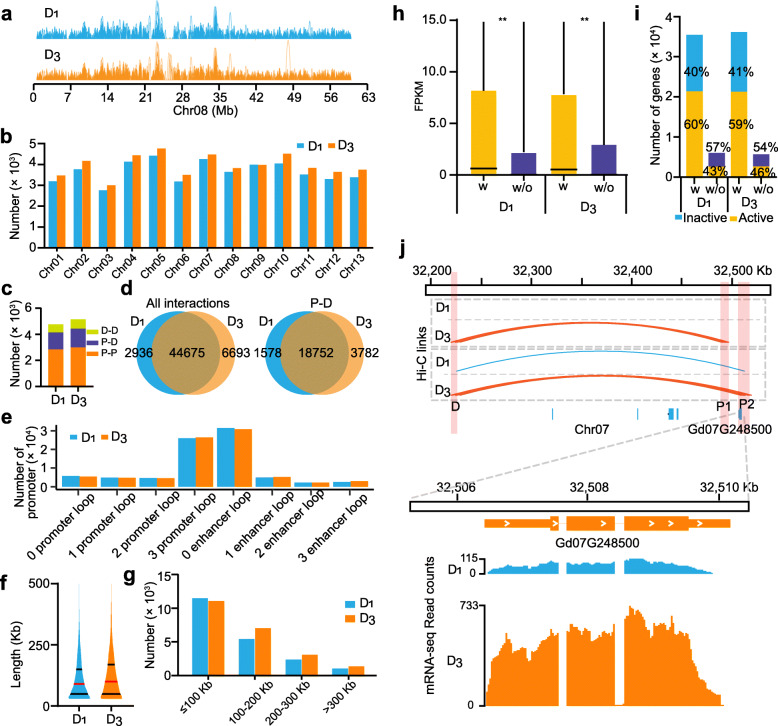
Long-range interactions between the proximal and distal regulatory regions. **a** An example of long-range interactions on Chr08 in *G. thurberi* and *G. davidsonii*. **b** Distribution of long-range interactions in each chromosome. **c** The long-range interactions were divided into the P-P, P-D, and D-D interactions. **d** Comparison of all interactions between *G. thurberi* and *G. davidsonii*. **e** Comparison of P-D interactions between *G. thurberi* and *G. davidsonii*. **f** Violin plots for long-range interactions in *G. thurberi* and *G. davidsonii*. The center red line in plot indicates the median, and the black lines indicate the upper and lower quartiles of insertion time. **g** Summary of the number of P–D interaction with variable distances in *G. thurberi* and *G. davidsonii*. **h** Comparison of expression level for genes interacting or not interacting with chromatin interactions (**P < 0.0001, a two-sided Wilcoxon signed-rank test). **i** Transcriptional status for genes with or without chromatin interactions. “Inactive” represents gene with FPKM < 0.1; “Active” represents gene with FPKM ≥ 0.1. “w” indicates genes with chromatin interactions; “w/o” indicates genes without chromatin interactions. **j** An example of one D interacted with two P in *G. davidsonii*. In the upper panel, the orange and blue lines represent Hi-C links in *G. thurberi* and *G. davidsonii.* respectively, and the blue boxes represent the genes locating in the interaction loop. The middle panel indicates the gene (Gd07G24850) around the P1 and P2. The lower panel is the read coverage generated by mRNA-seq

Long-range chromatin interactions functionally contribute to gene transcriptional regulation, but very little is known about 3D chromatin interactions in cotton. Seeking to characterize the pattern of long-range chromatin interactions, we conducted a genome-scale analysis and annotated the Hi-C peaks positioned within 2-kb upstream or 1-kb downstream of gene TSSs as “proximal Hi-C peaks” (P); all of the others were annotated as “distal Hi-C peaks” (D). We identified 22,328 P and 8304 D involved in long-range chromatin interactions in *G. thurberi*; *G*. *davidsonii* had 22,816 P and 8808 D involved in long-range chromatin interactions (Fig. 4a).

We also classified all of these interactions into three groups: proximal–proximal (P–P), proximal–distal (P–D), and distal–distal (D–D). A total of 47,604 and 51,367 intra-chromosomal interactions were identified for *G. thurberi* and *G*. *davidsonii*, respectively*.* Approximately 60% of these interactions were P-P interactions, followed by P-D (~ 30%) and D-D (~ 10%) (Fig. 4c). Comparison of the average number of formed loops is informative: we found that one D can form an average of 1.56 or 1.62 loops with P for *G. thurberi* and *G. davidsonii*, respectively; in contrast, one P can form an average of 1.34 or 1.31 loops with P. So, on average, D undergo more interactions than P, and it appears that genes regulated by D prefer to cluster together in the genome.

The number of interactions in each chromosome ranged from 2759 to 4417 in *G. thurberi* and 2999 to 4763 in *G. davidsonii* (Fig. 4b). There were 44,675 intra-chromosomal interactions were identified both in *G. thurberi* and *G. davidsonii*, while 2936 and 6693 interactions were specific to *G. thurberi* and *G. davidsonii*. We found 27,531 P-P interactions, 18,752 P-D interactions, and 5465 D-D interactions were conserved between *G. thurberi* and *G. davidsonii*, whereas 1043 and 2597 P-P interactions, 1578 and 3782 P-D, and 761 and 1067 D-D interactions were respectively specific for *G. thurberi* and *G. davidsonii* (Fig. 4d). This result emphasizes that only a small subset of intra-chromosomal interactions is divergent between cotton sister species.

Strikingly, more than 73% of the promoters of these cotton genomes have 3 or more P-P interactions, with most promoters having about 1 P-D (Fig. 4e). We found that the median length of intra-chromosomal interactions were 90 kb and 100 kb for *G. thurberi* and *G. davidsonii*, respectively (Fig. 4f). Previous studies in human have showed that enhancers prefer to regulate nearby genes [26]. Most of the P-D interactions were within 100 kb for both species, and fewer than 6% of these interactions were larger than 300 kb (Fig. 4g).

We next generated the transcriptome datasets using mRNA-Seq of *G. thurberi* and *G. davidsonii* leaves to help experimentally unravel the relationships between chromatin interactions and the transcriptional activation of cotton genes. We compared the expression profiles of genes with or without chromosomal interactions and found that genes with chromatin interactions had relatively higher expression levels than those without interactions (*P* < 0.0001, Wilcoxon rank-sum test) (Fig. 4h). Although the chromatin interactions were captured by Hi-C, we found that ~ 40% of the genes with interactions not expressed or expressed very lowly (FPKM < 0.1) (Fig. 4i). However, between 43 and 46% of genes which had no chromatin interactions in either *G. thurberi* and *G. davidsonii* were expressed in leaves, a level slightly higher than from a report for such genes in an analysis of shoots and immature ears in maize [26] (Fig. 4i).

We then examined the intersection of the differentially expressed genes between *G. thurberi* and *G. davidsonii* and differential P-D interaction genes to explore the possible roles of enhancers on the gene expression. Among the genes with differential P-D interactions between *G. thurberi* and *G. davidsonii*, there 509 genes which significantly altered expression levels, and these genes exhibited enrichment for GO terms including “response to biotic stimulus” and “defense response” (Additional file 2: Fig. S19). An example of these genes is the homeobox gene *Gd07G248500*, which encodes an ortholog of *AtHB16*, which is known to regulate leaf development and photoperiod sensitivity in *Arabidopsis thaliana* [27]. We found that the promoter of *Gd07G248500* interacted with 2 D peaks in both *G. thurberi* and *G. davidsonii*, but the interaction intensities were stronger in *G. davidsonii* than those in *G. thurberi* (15.13_vs_0.06 and 12.94_vs_1.2), which may promote its expression in *G. davidsonii* leaves (Fig. 4j), a situation like the maize PSB1 that had a shoot-specific P-D interaction with a higher expression in shoot than that in immature ear [26].

### Gene-order and structural variation between *G. thurberi* and *G. davidsonii*

To analyze gene order, a total of 32,981 orthologous pairs were identified between *G. thurberi* and *G. davidsonii*, among which 1104 orthologous gene pairs were located in the inversion regions (Table 2); these account for ~ 3.3% of the total analyzed orthologous gene pairs. The fact that this represents a higher proportion than that between the *G. hirsutum* cultivars TM-1 and ZM24 supports that more genes are affected by interspecies inversions compared to intraspecies inversions.

**Table 2:** Variations within genes between *G. thurberi* and *G. davidsonii* genomes

Variation type	Syntenic region		SV region	
	Number	Ratio	Number	Ratio
Structurally conserved genes ^a^	24,094	75.58	729	66.03
Without amino acid substitutions ^b^	2832	8.88	88	7.97
No DNA variation in CDS region	699	2.19	22	1.99
No DNA variation in CDS and intron region	292	0.92	10	0.91
No DNA variation in genic region ^c^	4	0.01	0	0.00
Same sense mutation	2133	6.69	66	5.98
With amino acid changes ^d^	21,262	66.70	641	58.06
With missense mutation in CDS	17,488	54.86	532	48.19
With 3n InDel in CDS	3774	11.84	109	9.87
Genes with large-effect mutations ^e^	2915	9.14	126	11.41
With 3n ± 1 InDel in CDS	1035	3.25	32	2.90
Start-codon mutation	694	2.18	29	2.63
Stop-codon mutation	641	2.01	31	2.81
Splice-acceptor mutation	32	0.10	0	0.00
Splice-donor mutation	513	1.61	34	3.08
Genes with large structural variations ^f^	4868	15.27	249	22.55
At least one CDS missing	4145	13.00	209	18.93
Total	32,981^g^	100.00	1104^g^	100.00

^a^ Structurally conserved genes, including genes without amino acid substitutions (b) and with amino acid changes (d). ^b^ Genes without amino acid substitutions (no DNA variation in the CDS region or intron regions). ^c^ Genic regions including 2 kb upstream and downstream of the gene body. ^d^ Genes with amino acid changes, including missense mutations in the CDS region and with 3n InDels in the CDS region. ^e^ Genes with large-effect mutations, including 3n ± 1 InDels in the CDS region, start-codon mutations, stop-codon mutations, splice-acceptor mutations, and splice-donor mutations. ^f^ Genes with large structural variations, including at least one CDS missing or other structural variation. ^g^ The total number of genes included for the analysis (genes and their orthologs in the counterpart genome, anchored on the 13 chromosomes)

We identified 21,262 (~ 67%) orthologous gene pairs with only missense mutations in their CDS or non-frameshift InDels between *G. thurberi* and *G. davidsonii*. However, only ~ 9% of the orthologous gene pairs had no amino acid changes between *G. thurberi* and *G. davidsonii*, and approximately 2% and 1% of these pairs had no variation in coding sequence (CDS) or gene bodies (CDS and intron regions), respectively (Table 2). These proportions are significantly lower than those from the comparison of *G. hirsutum* cultivars TM-1 and ZM24 (71%, 69%, and 56%), indicating that interspecies orthologs are more divergent than intraspecies homologs. Note that more than 9% of the syntenic orthologous genes pairs carried large-effect mutations, including 3n ± 1 InDel, start-codon mutation, stop-codon mutation, splice-acceptor mutation, and splice-donor mutation in the CDS regions (Table 2). More than 11% of the syntenic orthologous gene pairs were impacted by large structural variations, with 85% of these having lost at least one exon; any biological significance of these variations will require further study.

We also characterized extent of gene amplification in the *G. thurberi* and *G. davidsonii* genomes. More than 3400 tandem duplicated genes were identified in both *G. thurberi* and *G. davidsonii*, among which the stress-related pathways phenylalanine metabolism, glutathione metabolism, plant-pathogen interaction, and phenylpropanoid biosynthesis were found to be enriched in a KEGG analysis, indicating that tandem duplication has apparently enhanced the tolerance of *G. thurberi* and *G. davidsonii* to various stresses (Additional file 2: Fig. S20). In total, 3136 and 3154 genes were identified as singleton genes in *G. thurberi* and *G. davidsonii*, respectively (Additional file 1: Table S9). It was notable that there was a much higher proportion of transcription factors in the whole-genome duplication and segmental duplication sets than those from singleton genes (Fisher’s exact test, *P* < 2.2e−16), supporting our previous finding [4] that transcription factors have a tendency to be retained after duplication (Additional file 1: Table S10).

### Identification of centromeres using a Hi-C heatmap method

Centromeres are mainly composed of repetitive retrotransposons and satellite repeats, and the challenge of accurately assembling centromeres using short-read sequencing data is well-documented [28]; accordingly, centromere evolution is poorly understood. Previous studies of Hi-C matrices have shown that centromeres form a unique type of interacting subcompartment which can function as a barrier and prevent intra-chromosomal arm interactions [29]. By exploiting the insulation feature of centromeres in Hi-C heatmap data, we successfully developed a new method for centromere characterization based on Hi-C data.

**Fig. 5.**
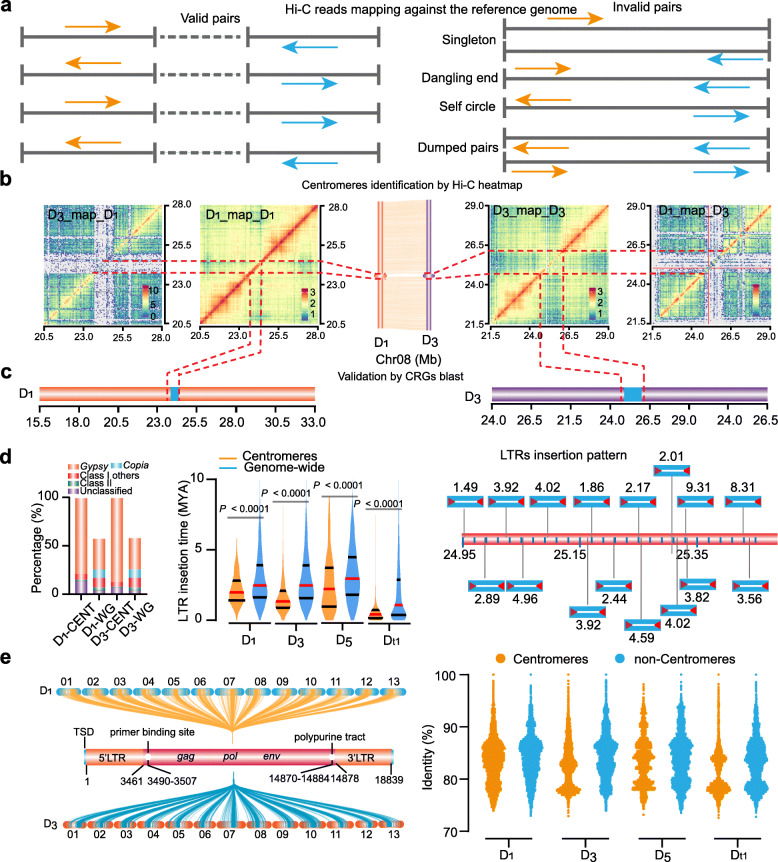
An overview of centromere identification based on Hi-C data. **a** A diagram of Hi-C data mapping against the reference genome. **b** Characterization of centromeres in Hi-C heat maps. The left panel shows chromatin interactions, including *G. davidsonii* mapped to *G. thurberi* (D_3__map_D_1_) and *G. thurberi* mapped to *G. thurberi* (D_1__map_D_1_). The middle panel presents a genomic alignment around the centromeres. The three-dimensional rings indicate the centromeres. The right panel shows chromatin interactions, including *G. davidsonii* mapped to *G. davidsonii* (D_3__map_D_3_) and *G. thurberi* mapped to *G. davidsonii* (D_1__map_D_3_). The regions within the orange lines are the centromere regions. **c** Validation the centromeres by centromeric LTR (Centromere Retroelement *Gossypium*, CRG) BLAST analysis. The data showed the validation on Chr08. **d** Centromere feature analysis. The right panel presents a comparison of the repetitive elements for centromeres vs. the whole genome. The middle shows LTR insertion time distributions for centromeres specifically, and for the whole genome. The center red line in the plot indicates the median, and the black lines indicate the upper and lower quartiles for insertion times. The right panel shows an analysis of the intact LTR insertion pattern. An example is presented for *G. thurberi* Chr04*.* The digits present the insertion time of nearby LTRs. **e** Analysis of centromere LTR enrichment. The left panel represents the sequence identity characteristic of a “CentLTR” sequence, as examined in centromeres and non- non-centromeric regions in four D genomes. The right panel is the identity distribution pattern of CenLTR hits presented as a dot plot. This analysis detected a total of 152,285 CenLTRs in D_1_ centromeres, with 163,217 in D_1_ non-centromeric regions; 158,815 in D_3_ centromeres, with 139,231 in D_3_ non-centromeric regions; 16,093 in D_5_ centromeres, with 76,875 in D_5_ non-centromeric regions; and 80,537 in Gh_D_t1_ centromeres, with 246,791 in Gh_D_t1_ non-centromeric regions

In this method, we first map the Hi-C contact data against its corresponding reference genome to obtain valid read pairs (Fig. 5a). Next, we use the valid read pairs to generate a Hi-C heatmap (at 50 kb resolution), and then use this to search regions which apparently form barriers to intra-chromosomal arm interactions. Testing confirmed that these regions, which have less frequent contacts between chromosome arms on either side compared with their frequency of intra-arm contact, are indeed centromeres (Fig. 5b). Thirdly, based on the phylogenetic relationship, we used the known cotton centromeric LTRs to align against the reference genomes to validate these Hi-C centromeres (Fig. 5c). Finally, the centromere sequence features—including sequence composition, LTR insertion time, LTRs insertion pattern, and centromeric enriched LTRs—can be cataloged systematically to support studies of centromere evolution (Fig. 5d,e). Using this new method (Additional file 2: Fig. S21), we successfully identified the centromeres in the model plant *Arabidopsis thaliana*, *Oryza sativa*, and the new *G. thurberi* and *G. davidsonii* assemblies (Additional file 2: Fig. S22-S25).

As we used nanopore long reads for our new genome assemblies, the centromeres are well assembled with the excellent coverage (Additional file 2: Fig. S26), thereby providing an unprecedented opportunity to study cotton centromere evolution. As we aligned *G. thurberi* against the *G. davidsonii* genome, we clearly found that there were no collinearities in the middle region of each orthologous chromosome (Additional file 2: Fig. S27). Chromosomal collinearity analysis showed that many non-syntenic regions were located in the centromeric regions (Additional file 1: Table S11), indicating that the centromeric regions have higher divergence compared to their neighboring (flanking) regions.

To further support this, we aligned the previously reported *G. raimondii* and *G. hirsutum* CENH3 ChIP-Seq data against the four genomes available for D genome species (*G. thurberi*, *G. davidsonii*, and *G*h_D_t1_) (Additional file 2: Fig. S28). We detected a strong peak in a narrow region on *G. raimondii* Chr08 when mapping *G. raimondii* ChIP-Seq data (Additional file 2: Fig. S28a). However, upon mapping *G. raimondii* ChIP-Seq data against the other three examined *Gossypium* genomes (*G. thurberi*, *G. davidsonii*, and *G. raimondii*), the signals were dispersed over a broader region, with no obvious major peaks. Mapping *G. hirsutum* CENH3 ChIP-Seq data against the *G. hirsutum* genome revealed an apparent peak on D12; no major peaks were detected when we mapped this data to the four other D genomes (Additional file 2: Fig. S28b). These findings underscore that centromeric regions can be highly divergent among closely related species.

We also mapped the *G. thurberi* Hi-C data against the *G. davidsonii* assembly and vice versa, we observed large gaps in the centromeric regions; this indicates that centromeric sequences from the orthologous chromosomes in *G. thurberi* and *G. davidsonii* were highly divergent (Fig. 5b and Additional file 2: Fig. S3-4). Although the centromeric regions are highly divergent (without any syntenic blocks), we found that the flanking regions of the centromeres are highly conserved with good collinearities. For Chr03, Chr04, Chr07, and Chr08, no large-scale inversions were detected between orthologous chromosomes, highlighting that chromosomes arms are highly syntenic and lack obvious changes in their centromeric positions. Although there were inversions located in the chromosome arms in Chr05, Chr06, and Chr10, we observed that these inversions had no effect on centromere locations, since the centromeric flanking regions retained synteny. Chr01, Chr02, Chr09, Chr11, Chr12, and Chr13 experienced pericentromeric inversions; that is, we observed that the collinearities of flanking regions were reversed between the two genomes, suggesting that inversions spanning the centromere occurred after divergence.

### Centromere LTRs have undergone rapid changes

We next examined whether there were any local sequence similarities among the centromeres from non-homologous chromosomes. We used the NCBI blastn tool to align the centromere sequences, and filtered the results with a loose filter (block length larger than 2000 bp with 95% identity). We observed that the centromeric sequences are highly repetitive, and detected more similar sequences from the intraspecies comparison than the interspecies comparison, indicating that centromeres have experienced duplication after speciation (Additional file 2: Fig. S29). Moreover, we found that the sequences from *G. davidsonii* are more similar, indicating that the duplications occurred later than those from *G. thurberi*.

The DNA sequences of plant centromeres usually contain many copies of simple tandem repeats, which occur in head-to-tail arrays; only those which are associated with CENH3 nucleosomes are considered to be part of the functional centromere [30]. However, our understanding of the role of these sequences in centromere function remains rudimentary at best. Unlike centromere tandem repeats in many plants [31], we found that the tandem repeat content is very low in *G. thurberi* and *G. davidsonii* (Fig. 5d)*.* Instead, we observed strong enrichment for LTRs (especially for *Gypsy*-type retrotransposons), suggesting that cotton centromeres have arisen from retrotransposons.

We used Kimura to analyze LTR insertion times, which revealed that LTRs in centromeres are younger than those at the whole-genome level among all D genomes (D_1_, D_3_, D_5_ and Gh_D_t1_) (Fig. 5d). The LTRs in *G. davidsonii* centromeres are younger than those from *G. thurberi* (median of 1.336 MYA vs. 1.979 MYA), indicating that centromeres in *G. davidsonii* have been much more active than those of *G. thurberi* and supporting that the centromeres in *G. davidsonii* experienced expansion compared with those from *G. thurberi* (Fig. 5d). Unlike the nested insertion of full-length LTRs previously reported for *Brassica nigra* and some cereal centromeric regions [20], we detected full-length LTRs that were independently inserted into the centromeric region, e.g., in Chr04 of *G. thurberi*, and we identified 16 intact *Gypsy*-type LTRs that have inserted into centromeres between 1.49 and 9.31 MYA (Fig. 5d).

We constructed a phylogenetic tree of all the LTRs to describe the pattern of diversity (Additional file 2: Fig. S30). Three subclades were mainly found in the centromeric region; these were all quite distinct in sequence from the D cotton genome LTRs from non-centromere regions (Additional file 2: Fig. S30a). Moreover, we found that the LTRs from *G. davidsonii* tend to cluster together in the phylogenetic tree, as did those from *G. thurberi*, findings which indicate that the LTRs of the centromeres in *G. thurberi* and *G. davidsonii* have proliferated and spread after these two species diverged from their common ancestor (Additional file 2: Fig. S30b).

We next identify and characterize the centromeric LTRs by mapping all the intact LTRs in the *G. thurberi* and *G. davidsonii* genomes with blastn. One LTR from Chr12 (26,780,294–26,783,754) of *G. thurberi* had significant BLAST hits for centromeres of each orthologous chromosome in *G. thurberi* and *G. davidsonii* (Fig. 5e), and we detected a variety of highly similar sequences throughout the centromeres (this LTR type was designated as “CenLTR”). Further, alignments clearly indicated strong divergence from centromere LTR types (GhCR1-GhCR4) from *G. hirsutum* (Additional file 1: Table S12)*.*

We further aligned the *G. raimondii* and Gh_D_t1_ genomes and found that the CenLTRs are also enriched in the centromeric their regions, indicating that CenLTRs are apparently widely distributed in the centromeres of D genome species. We compared the sequence identities between the centromeres and the non-centromere sequences for each species. A lot of CenLTR polymorphisms were detected between *G. davidsonii* centromeres and *G. davidsonii* non-centromere sequences (Fig. 5e). Similar CenLTR polymorphisms were evident between Gh_D_t1_ centromeres and non-centromere sequences (Fig. 5e). Surprisingly, the identity with consensus sequence was lower in the centromeric regions compared with non-centromeric regions (Fig. 5e), indicating that the LTRs have undergone rapid changes in the centromeres.

### Divergent evolution of genes involved in stress tolerance

**Fig. 6.**
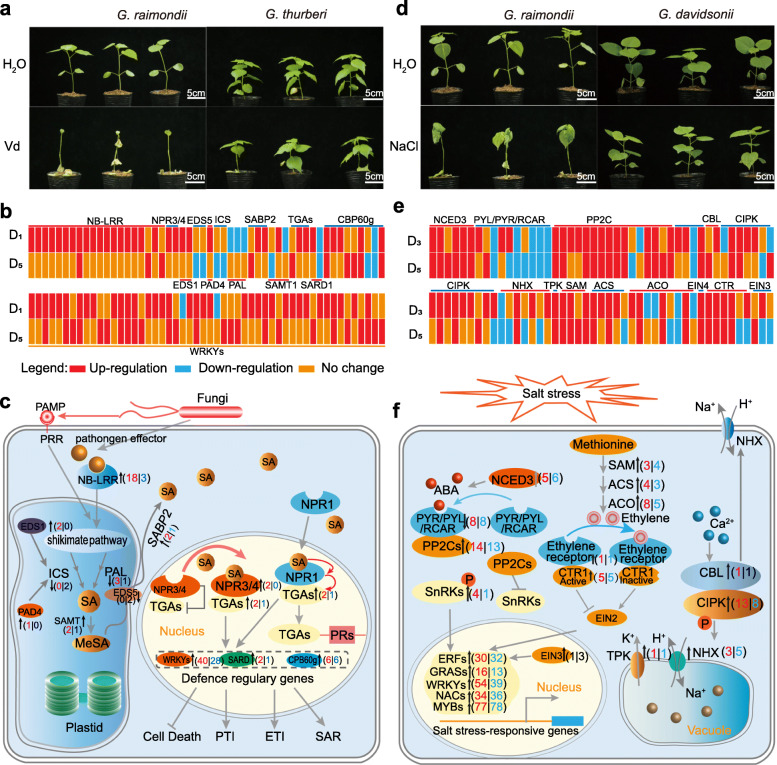
Models depicting the molecular basis of *Verticillium* wilt and salt stress tolerance in *G. thurberi* and *G. davidsonii*. **a** Phenotypic comparison of *G. thurberi* (D_1_) and *G. raimondii* (D_5_) seedlings (35-day-old seedlings) in response to challenge with *Verticillium dahliae*. Photographs were taken under normal conditions or 14 days after challenge with *Verticillium dahliae*. **b** Heat maps for differentially expressed genes with annotations related to salicylic acid (SA) signaling, NB-LRR, and WRKYs. Genes with an adjusted *P* value < 0.05 and an absolute value of log_2_[foldchange] > 1 found by EdgeR were designated as differentially expressed. **c** A proposed model showing that the SA signaling pathways enhance *Verticillium* wilt tolerance in *G. thurberi*. *V. dahliae* attack induces SA biosynthesis via the isochorismate synthase (ICS) and phenylalanine ammonia-lyase (PAL) pathways in plastids. Enhanced disease susceptibility (*EDS1*) and phytoalexin deficient 4 (PAD4) are required for increased SA accumulation. SA methyltransferase (SAMT) catalyzes SA to MeSA, which diffuses into the cytoplasm, where it is converted back to active SA by SABP2. The red and blue digits in brackets represent the upregulated genes in D_1_ and D_5_, respectively. **d** Phenotypic comparison of *G. davidsonii* (D_3_) and *G. thurberi* (D_1_) seedlings in response to salt stress treatment (250 mM NaCl watering 21-day-old seedlings every 2 days). Photographs were taken under normal conditions or 14 days after treatment with NaCl solution. **e** Heat maps for differentially expressed genes with annotations related to ABA, ethylene, and CBL-CIPK pathways. Genes with an adjusted *P* value < 0.05 and an absolute value of log_2_[foldchange] > 1 found by EdgeR were designated as differentially expressed. **f** Transcriptional network related to salt response in *G. raimondii* and *G. davidsonii*. Ethylene biosynthesis, calcium signaling, and vacuole NHX are activated in *G. davidsonii*. The *NCED3* gene encodes the enzyme which catalyzes the first step of ABA biosynthesis. The ABA signaling pathway, comprising PYR/PYL/RCAR, PP2C, and SnRKs proteins, is a major plant hormone involved in salt stress responses. Ethylene biosynthesis is catalyzed by the SAM, ACS (ACC synthase), and ACO (ACC oxidase) enzymes. The ethylene signaling pathway includes ethylene receptor, CTR1, and EIN2. TPK (two-pore potassium) is K+ channel that trafficks K+ out of the vacuole. NHX1 (tonoplast-based Na+/H+ exchanger) is required for sequestration of excessive Na^+^ and Cl^−^ in the vacuole. The red and blue digits in the brackets represent the upregulated genes in D_3_ and D_5_, respectively

As the D subgenome donor of the widely cultivated upland cotton, *G. raimondii* is known to have contributed stress tolerance traits to allotetraploid cotton [32]. Nevertheless, allotetraploid cotton is sensitive to *Verticillium dahliae* infection and to growth in high salinity soils; these represent major challenges facing cotton production worldwide, and a lack genetic resources for improving plant tolerance to these challenges is a major constraint in current cotton breeding programs. Here, we found that *G. thurberi* seedlings are more tolerant to *Verticillium dahliae* than *G. raimondii*, indicating that *G. thurberi* is a promising resource for upland cotton improvement (Fig. 6a). We identified 3472 and 5042 genes associated to tolerance to *Verticillium dahliae* in *G. thurberi* and *G. raimondii*, respectively. We identified a total of 106 genes including *NB-LRR*, *NPR1/3/4*, *TGA*, and downstream transcriptional factors (e.g., WRKY33, SARD1, and CPB60g) potentially involved in disease responses based on their differential responses to the *Verticillium dahliae* treatments between *G. thurberi* and *G. davidsonii* (Fig. 6b). The SA biosynthesis signal pathway was activated in *G. thurberi*, as the *PAD4*, *EDS1*, *SAMT*, and *SBPB2* genes were upregulated in *G. thurberi* upon *Verticillium dahliae* challenge (Fig. 6c)*.* We overexpressed *WRKY33* (*Gthurberi12G176500*) genes in *G. hirsutum* to test whether the genes from wild cotton can be used in cultivated cotton improvement. As expected, the overexpression lines displayed improved upland cotton tolerance to *Verticillium dahliae*, indicating that *G. thurberi* can be understood as an important genetic resource for cotton breeding (Additional file 2: Fig. S31).

Unlike *G. thurberi*, *G. davidsonii* displayed significant salt tolerance in seedlings when compared with *G. raimondii* (Fig. 6d). A total of 14 ethylene-related genes (including *SAM*, *ACS*, *ACO*, *EIN4*, *CTR*, and *EIN3*) showed differential responses to salt treatment between *G. davidsonii* and *G. raimondii* (Fig. 6e). Genes of the *CBL-CIPK* pathway *s*howed differential responses to salt between *G. davidsonii* and *G. raimondii*, with the *CIPK* and *NHX* genes being upregulated by salt treatment of *G. davidsonii* (Fig. 6e)*.* Moreover, we found that other well-known stress-related genes including *ERFs*, *GRASs WRKY*, *NACs*, and *MYBs* were upregulated in *G. davidsonii* upon salt treatment (Fig. 6f); such genes have likely played important roles in species divergence and have likely contributed to the spread of the cotton D genome sister species in their adaptation to new ecological contexts and environments.

## Discussion

The most outstanding advantage of long-read sequencing is that it provides more comprehensive coverage for the genome, which is often most obviously reflected by its increased capacity to accurately capture highly repetitive sequences. There was 39.4 to 46.9% of genome assembly identified as repetitive elements in the diploid draft D genomes by short-read sequencing [7], here the annotated repetitive DNA increased to approximate 60% of total assemblies.

Centromeres are known to comprise highly repetitive elements that are structures essential for the maintenance of karyotype integrity during meiosis, ensuring the fertility of developed gametes through strict inheritance of full chromosome complements [33]; nevertheless, centromeres are enigmas in many genome assemblies. Centromeres exhibit profound complexity; their length ranges from only hundreds of base pairs to multi-megabases. The simplest “point” centromeres are only ~ 125 bp and found in yeasts, and their sequences are conserved among sister species. In contrast, “regional centromeres” are significantly variable both in size and sequence and can be hundreds of kilobases in length [34]. Some regional centromeres contain non-repetitive sequences, e.g., *Candida albicans*, or have a mixture of repetitive sequence and non-repetitive sequence (e.g., chicken and horse) [31]. A previous study showed that Ty3-gypsy-like LTRs are localized to the centromeric region of all the chromosomes of upland cotton and the B-, D-, and E-genome diploid cottons [35]. In the present study, we found these large numbers of gypsy-like LTR in the centromeric regions account for more than 75% of the total centromere length, strongly supporting that the cotton centromeres originated from retrotransposons.

The precise centromeric DNA sequences of the *G. thurberi* and *G. davidsonii* vary dramatically, and it has been proposed that this rapid evolution could be a consequence of meiotic drive [13, 16]. Comparison between the closely related species human and macaque showed that some centromeres adopt new positions over evolutionary time subsequent to a speciation event, without transposing any surrounding genetic markers. These structures are referred to as evolutionarily new centromeres and have been observed in primates and other mammals [13]. Unlike the human and macaque, for cotton (except for the Chr11) the centromere location is highly conserved between the orthologous chromosomes in *G. thurberi* and *G. davidsonii*.

Hi-C sequence data has recently supported a new era of studies about genome-wide 3D genome structural organization. Previous work in cotton examined how chromatin architecture reorganization may have been affected during polyploidization, in analyses based on comparisons of allotetraploid cotton with the possible progenitor of diploid cotton [24]. In the present study, we compared the 3D genome of three sister D genome species, and our observations of both A/B compartment switching and the reorganization of TADs among the diploid D genomes provide new insights into the effects of genome divergence on the spatial organization of chromatin. Specifically, we found that the DNA sequence surrounding the TAD boundaries displayed a lower level of 5mC (CG, GHG, and CHH) and 6mA modifications than the genome as a whole; these epigenetic modifications have been functionally associated with gene transcription in previous studies and may play roles in the activation of genes positioned near TAD boundary genes.

## Conclusions

In summary, we de novo assembled very high-quality reference genomes for two important cotton germplasm resources using technologically complementary sequencing technologies. Based on these reference-grade genome assemblies for *G. thurberi* and *G. davidsonii*, we comprehensively evaluated genome arrangements, small variations (SNPs, InDels, and PAVs), gene order, long-range interactions between proximal and distal regulatory regions, and gene structure variations in these closely related species to better understand their divergence process. Of particular note, our Nanopore long-read data, in combination with a new method we developed for centromere characterization based on Hi-C data, ultimately revealed insights about the previously mysterious process of centromere evolution in plant genome. Our study also identified multiple genetic networks underlying the unique traits that have long made these D genome cotton species interesting to crop scientists. Thus, our work deepens understanding of crop evolution, centromere divergence, and trait diversification and indicates a way forward for harnessing genes that confer agronomically beneficial traits as useful resources in cotton breeding programs.

## Methods

### Plant growth conditions

*G. thurberi* and *G. davidsonii* plants (collection from National Wild Cotton Nursery, Sanya, China) were grown in a greenhouse for 180 days, and the young leaves from a single plant were harvested and immediately frozen in liquid nitrogen to extract their genomic DNA. For *Verticillium* wilt analysis, *G. raimondii* and *G. thurberi* seedlings were planted in a mixture of sand and vermiculite. Once they had two true leaves, they were treated with *Verticillium dahliae* via the root dipping method according to the methods used in a previous study [36]. For salt tolerance analysis, *G. raimondii* and *G. davidsonii* were planted in vermiculite and watered with 350 mmol of NaCl every 2 days. All of the phenotypic photos were taken 14 days after treatment. For mRNA-seq analysis, the seedlings were hydroponically cultivated according to previously used methods [36]. Seedlings at the two-true-leaf stage were treated with a liquid medium containing 250 mmol of NaCl; seedlings grown in a normal liquid medium were used as the control. The samples were harvested at 3 h, 6 h, and 24 h following treatment, after which they were immediately frozen in liquid nitrogen and stored at − 80 °C for subsequent analysis.

### Nanopore sequencing

An improved CTAB method [6] was used to extract the genomic DNA of *G. thurberi* and *G. davidsonii*. Two micrograms of gDNA was repaired using a NEB Next FFPE DNA Repair Mix kit (M6630, USA) and subsequently processed using the ONT Template prep kit (SQK-LSK109, UK) according to the manufacturer’s instructions. Large segment libraries were premixed with loading beads and washed with an R9 flow cell. The library was sequenced on the ONT PromethION platform with a corresponding R9 cell and ONT sequencing reagent kit (EXP-FLP001.PRO.6, UK) according to the manufacturer’s instructions.

MinKNOW software was used to collect the sequencing data in real-time and process it into basecalls. Single-molecule sequencing was performed on a PromethlON system and yielded a total of 3,575,506 and 3,237,739 filtered subreads with average lengths of 31,963 bp and 33,450 bp for *G. thurberi* and *G. davidsonii*, respectively. Finally, only nanopore subreads equal to or longer than 500 bp were used to generate the two genome assemblies.

Total RNA exaction and cDNA synthesis were performed according to the methods described by Yang et al. [4]. The BluePippin™ Size Selection System (Sage Science, USA) was used to identify and select the requisite sizes (1–2 kb, 2–3 kb, and > 3 kb), and a Pacific Biosciences DNA Template Prep Kits v.2.0 was used to build the SMRT bell libraries. We conducted the SMRT sequencing using the Pacific Bioscience Sequel platform according to the manufacturer’s instructions.

### Illumina sequencing

We constructed libraries with a 350-bp insert fragment for *G. thurberi* and *G. davidsonii* according to the manufacturer’s instructions (Illumina). A HiSeq 2500 system was used to sequence the libraries, along with a PE150 strategy according to the manufacturer’s instructions (Illumina). The sequence adaptors were removed for the uncleaned Illumina reads, and the contaminated reads (viral, mitochondrial, bacterial sequences) were compared with the NCBI-NR database via BWA v0.7.13 [37] (using default instructions). Duplicate pairs were identified using FastUniq v1.12 [38]. In total, we produced 116.9 Gb and 118.2 Gb clean Illumina reads for *G. raimondii* and *G. davidsonii*, respectively. For CENH3 analysis, the raw sequencing data were downloaded from the Gene Expression Omnibus for *G. hirsutum* (accession number GSE119184) [39] and the European Molecular Biology Laboratory-European Bioinformatics Institute (accession number PRJEB14368) [40]. The data were aligned to the reference genome with Botiwe2, and the enrichment was calculated by dividing the CENH3 read counts by the input read counts according to previously used methods [28].

### De novo assembly

Canu [41] (https://github.com/marbl/canu, v1.5) was used to select longer seed reads with the settings “genomeSize = 1000000000” and “corOutCoverage = 50,” after which raw overlapping reads were detected using a highly sensitive overlapper MHAP (mhap-2.1.2, option “corMhapSensitivity = low/normal/high”). An error correction was then performed using falcon_sense method (option “correctedErrorRate = 0.025”’). Smartdenovo (https://github.com/ruanjue/smartdenovo) was used for assembly. Racon software was used for error correction, and Pilon software was used for adjustment [42].

The two assemblies were evaluated by mapping 1440 Benchmarking Universal Single-Copy Orthologs to the genomes using BUSCO v3.0.2 b [17], which showed 1372 (95.28%) and 1374 (98.42%) complete BUSCOs and 18 (1.25%) and 14 (0.97%) fragmented BUSCOs in the *G. thurberi* and *G. davidsonii* assemblies, respectively.

### Hi-C sequencing data

The Hi-C libraries construction and sequencing were performed according to the methods described by Yang et al. [4]. After the reads were filtered, we obtained 284.3 million and 280.3 million valid interaction pairs for the chromosome-level assembly of *G. thurberi* and *G. davidsonii*, respectively. The assembly contigs were separated into 50-kb fragments, and LACHESIS software [43] was used to cluster those that remained with valid interaction read pairs; finally, 74 and 104 contigs with respective total lengths of 779.6 Mb and 801.2 Gb were anchored and oriented to their 13 chromosome-level groups of *G. thurberi* and *G. davidsonii*, respectively.

### Repeated sequence prediction

The repeated sequences were identified according to the methods described by Yang et al. [4]. The repeated sequences occupy 57.96% (451.8 Mb) of the *G. thurberi* assembly and 58.58% (469.4 Mb) of *G. davidsonii* assembly, respectively, of which *Gypsy* retrotransposons account for more than 31% in both assemblies. The insert time was calculated using the solo and intact LTRs according to the following: time = *K*/2*r* (*K* is the distance between all of the alignment pairs; *r* is the rate of nucleotide substitution). The *r* value^9^ was considered to be 7 × 10^−9^, while we used the distmat program to calculate *K*. All of this was performed using the EMBOSS^30^ package according to the Kimura two-parameter model.

### Protein-coding gene prediction

We used Iso-Seq, protein homology, and de novo methods. De novo gene prediction entailed using Genscan v1. 0 [44], Augustus v2. 4 [45], GlimmerHMM v3.0. 4 [46], GeneID v1. 4 [47], and SNAP [48]; the homologous peptides were aligned to the assemblies from *Oryza sativa* L. ssp. japonica, *Arabidopsis thaliana*, *G. raimondii* (JGI), and *G. hirsutum* (CRI) using GeMoMa v1.4. 2 [49], and BLAT [50] was used to align the consensus isoforms derived from PacBio long cDNA reads to the repeat-masked assemblies. Lastly, PASA [51] was used to analyze the gene structures of the results of the BLAT alignment. We used EVidenceModeler [52] to combine the protein alignments, transcript information, and de novo predictions to produce a unifying model for the gene. In total, 41,316 and 41,471 genes were predicted for *G. thurberi* and *G. davidsonii*, respectively, whereas 37,533 and 38,755 genes were annotated in the previously reported draft genomes for *G. thurberi* and *G. davidsonii* [7], respectively. We annotated the expected genes by comparing their sequences with several protein sequence and nucleotide repositories, including NR, COG, KEGG, and TrEMBL, using an *e*-value cutoff of 1e−5. Blast2GO was used to designate gene ontology (GO) terms for all genes based on NCBI databases. BUSCO v3.0.2 was compared with embryophyta_odb10 database to ensure the gene set was complete, compared to the reference genome sequences. More than 97.8% of the BUSCO genes were complete and only 0.2 % of BUSCOs were missing. This indicates that our gene prediction is accurate.

### Paralog analysis for *G. thurberi* and *G. davidsonii*

The all-against-all BLASTP method (*e*-value <1e−5) was used to detect paralogous genes in *G. thurberi* and *G. davidsonii*. Homologous blocks were then detected using MCScanX v1.1 [53], requiring at least five collinear gene pairs within one block and fewer than 25 intervening genes.

### Identification of SNPs, InDel, inversions, and PAVs

We used MUMmer v4.0 [54] (http://mummer.sourceforge.net/) to identify the SNPs and InDel between *G. thurberi* and *G. davidsonii* according to the following procedures: (1) each query genome was aligned with the corresponding reference genome using the nucmer utility under the parameter “—mum,” (2) the delta-filter utility was used to filter mapping noise and determine the one-to-one alignment blocks with the parameters “-1 -r –q,” and (3) the show-snps utility was used for calling the SNP and small InDels(< 100 bp).

Inversions were obtained by screening nucmer outputs with a delta-filter utility using two parameters: “-i 90 -g -r -q” and “-i 90 -1 -r -q,” in which “-1” identified one-to-one alignment blocks for rearrangements, and “-g” identified collinear regions with global alignments and no rearrangements. Alignment blocks with translocated sections associated with global alignments under the -g parameter were considered allelic and were not included. We identified non-allelic parts by comparing the allelic locations from the alignment blocks generated by the “-1” parameter, which were considered translocations or inversions based on their positioning in the overall region. Delta filtering was used to screen nucmer output, with a minimum identity of 90%. Homology blocks not associated with the allelic locations were considered translocations or inversions based on their positioning in the overall region. The PAV of *G. thurberi* or *G. davidsonii* were called by ppsPCP ( --coverage 0.5 --sim_pav 0.9).

### Genes and their structural variations analysis

The one-to-one orthologous genes among *G. arboreum*, *G. thurberi*, *G. davidsonii*, *G. raimondii*, and *G. turneri* were identified using Inparanoid v4. 1 [55]. In some instances, no matching counterpart was detected in the subject genome for an ortholog in the query genome. In these cases, the GeMoMa software was used to verify its absence in order to avoid a prediction error. In total, we obtained 31,319, 32,981, 26,237, 25,925, 31,130, and 26,057 orthologous gene pairs for *G. davidsonii*-*G. raimondii*, *G. davidsonii*-*G. thurberi, G. davidsonii*-*G. turneri*, *G. raimondii-G. turneri*, *G. thurberi*-*G. raimondii*, and *G. thurberi*-*G. turneri*, respectively. Similarly, we identified 28,704, 28,495, 28,631, and 25,063 orthologous pairs between *G. arboreum* and *G. davidsonii*, *G. raimondii*, *G. thurberi,* and *G. turneri*, respectively. The CLUSTALW was used to align the coding and protein sequences of the orthologous pair, while the *Ka* and *Ks* values were calculated using the perl module, “Bio::Align::DNAStatistics.” Divergence time estimation was followed a recent study in allotetraploid cotton [56].

We assessed the genetic variations within each orthologous gene pair according to the following parameters: (1) we identified the longest transcript within each gene loci as a candidate; (2) we obtained the gene coding regions 2 kb upstream and 2 kb downstream and compared them with their associated genomic regions with BWA; (3) we considered structurally conserved genes to be orthologous genes with the same number of exons but with asynchronous differences and a lack of certain codons; (4) large-effect variations were considered to be orthologous genes with the same number of exons, but varying lengths, different splice sites, or mutations in their frameshifts; (5) we considered the orthologous genes that were left to have significant structural differences.

### Gene family expansion analysis

Protein sequences with lengths less than 20 amino acid residues were filtered. The blastp program was used to perform all-vs-all alignments with “-evalue 1e-5, -outfmt 6.” OrthomclBlastParse within OrthoMCL [57] was used to filter the blastp results to become inputs of the Mysql database. The orthmclPair was used to identify potential protein pairs, while orthmclDumpPairFiles was used to obtain the orthologous pairs. The mcl was used to cluster the output of the orthmclDumpPairFiles, while orthomclMclToGroups was used to group the orthologous pairs. The unique genes were analyzed based on grouped orthologous pairs. A total of 7561 genes were identified as single-copy genes, and their protein sequences were combined as an ultra-long fasta species by species. The sequences were then aligned via MAFFT [58], and the conserved sites were extracted by Gblocks [59]. The optimal amino substitution model evaluated by Protest software [60] was “PROTGAMMAJTTX” for phylogenetic tree analysis using RAxML [61]. Previous studies, including this one, demonstrated that the diploid A and D genome species were divergent at ~ 5 MYA and that *G. thurberi* and *G. davidsonii* were divided at 1.40–1.60 MYA. These two time points were used to fix the node divergent time in the r8s [62] analysis. CAFE (http://heanet.dl.sourceforge.net/project/cafehahnlab/cafe.linux.x86_64) was used to evaluate the expansion and contraction of the gene family. A total of 2228 families experienced expansion and contraction.

### Identification of the centromeres

The relatively conserved 5′ LTR sequences (GhCR1-5′ LTR, GhCR2-5′ LTR, GhCR3-5′ LTR, and GhCR4-5′ LTR), which are related to centromeres, have been identified in cotton [35]. Here, we aligned these LTR sequences against the *G. thurberi* and *G. davidsonii* genomes using blastn with sequence similarity ≥ 80% and *e*-value ≤1e–20. After filtering the alignments, we used the R t.test function to calculate the 95% confidence interval for the median, which represents the centromeric region for each chromosome. Previous studies have demonstrated that centromeres form a barrier to intra-chromosomal arm interactions, resulting in less frequent contacts between the chromosome arms on either side compared with the frequency of intra-arm contact [29]. This insulation property is visible on contact maps and can be used to identify centromeres. The Hi-C reads of the *G. thurberi* were truncated at the putative Hi-C junctions using the *Hin*dIII restriction sites, followed by alignment to the D1 reference genomes using bwa (version 0.7.10). The uniquely mapping read pairs with a mapping quality greater than 20 were kept for further analysis. Invalid read pairs, including dangling-end and self-cycle, re-ligation, dumped products, and PCR duplicates were filtered using HiC-Pro software (v2.10.0). The R ggplot2 package was used to draw the Hi-C heatmaps at different resolutions. In our new assemblies, we also observed insulation features in the Hi-C heatmaps: *G. thurberi* and *G. davidsonii* centromeres lack a strong interacting signal and create a barrier within each chromosome, resulting in the expected trend for contacts in plant genomes: less frequent contacts between the chromosome arms on either side of them compared with intra-arm contact frequencies (Additional file 2: Fig. S24-25). We used the Hi-C heat maps to shrink the centromeric region as the centromeres displayed a significantly reduced interaction with the neighboring regions. We found that centromeric regions overlapped with the result obtained by GhCRs blast. Therefore, the centromeric regions identified by Hi-C were used in further analysis.

### Methylation sequencing analysis

We used Basecall with ONT’s Guppy software to convert fast5 format data to the fastq format for QC analysis. The original fastq data were further filtered to remove adapters, short reads (length < 500 bp), and low-quality reads (MeanQual < 6). From this, we obtained the total data set. Sequencing depth and alignment efficiency were counted by aligning clean-read positions at the reference genome after mapping them to the reference genome with mimimap2 software. The minimap2 software uses split-reads and heuristic methods to reduce false comparisons, which are suitable for comparing long-read, high-noise reads with reference genes. We used nanopolish, based on the hidden Markov model, to detect CpG. Tombo was used to detect CHH (H = A/T/C), CHG, and 6mA sites. The bisulfite sequencing (Bs-seq) and data analysis was performed according to a previous report [24].

### mRNA-seq analysis

We used hisat2 to map the clean reads (Additional file 1: Table S13-14) to the reference genome, and Stringtie to calculate the gene expression level and read counts [63]. For differentially expressed genes, we used the DESeq2 package in R, based on the negative binominal distribution. Genes with absolute value of log_2_[fold change] > 1, and Benjamini–Hochberg-adjusted *P* < 0.05 are considered DEGs. GO enrichment was performed using the R package “goseq,” and the package “qvalue” was used to adjust the *P* value. Only GO terms with adjusted *P* value < 0.05 were considered significant.

### Identification of A/B compartment, TAD boundaries, and long-range interactions

The clean reads were mapped against their corresponding reference genomes with the bowtie2 software (version 2.2.3). Based on a Hi-C Pro pipeline, only the uniquely mapped paired-end reads were used for subsequent analysis [64]. The contact matrices were generated at different resolutions using the robust remove multiplex bias method ICE. The A/B compartments were analyzed based on 50 kb resolution ICE matrices using HiTC (version 1.24.0). The eigenvalue of the first principle components was plotted as the compartment assignment, with positive values corresponding to high gene density (compartment A) and negative values corresponding to low gene density (compartment B).

TAD calling was performed by TadLib [65] with a 20-kb resolution. The inclusion ratio (IR) for each TAD was calculated by HOMER [66], with only IR values greater than 1 used in subsequent analysis. TAD boundaries were identified from the calling results of the above TAD. We compared the different directionality index (DI) delta scores of the TAD boundaries between two samples to identify dynamic TAD boundaries. A TAD boundary was considered dynamic when its adjusted *P* value (as calculated by LIMMA (v3.30.13) [67]) was less than 0.1 and it had DI delta scores greater than 70 for one sample and smaller than 70 for the other sample with a fold change of DI delta scores larger than 2. If an adjusted *P* value was larger than 0.1 and the DI delta score for one sample was four times as large as the other sample, the TAD boundary was also deemed as dynamic [68]. A hierarchical clustering heatmap was drawn for all dynamic TAD boundaries based on their DI delta scores.

The Fit-Hi-C (v2.05) tool was used to identify the Hi-C interaction peaks with “-r 10,000, -U 1000000, -L 20000, -x intraOnly.” Then, the *q*-value ≤ 0.00001 was used to filter the candidate interaction sites. HOMER was used to calculate the differential chromosomal interactions. Hi-C peaks 2-kb upstream or 1-kb downstream of the TSS of the genes were annotated as proximal Hi-C peaks (P), and the others were annotated as distal Hi-C peaks (D).

### Statistical analysis

Comparison of methylation levels between the A and B compartments was carried out by using a two-sided Wilcoxon rank-sum test.

## Supplementary Information

**Additional file 1: Tables S1-S14**. **Table S1**. Summary of Nanopore sequencing clean data in *G. thurberi* and *G .davidsonii*. **Table S2**. Nanopore sequencing reads length distribution in *G. thurberi* and *G. davidsonii*. **Table S3**. Genome assembly statistic information for *G. thurberi* and *G. davidsonii*. **Table S4**. Summary of Hi-C contact data mapped against the *G. thurberi* and *G. davidsonii.* **Table S5**. Comparison of repetitive elements between *G. thurberi* and *G. davidsonii*. **Table S6**. The Illumina short reads from *G. thurberi* and *G. davidsonii* mapping against *G. thurberi* and *G. davidsonii* assemblies respectively. **Table S7**. Evaluation of the *G. thurberi* and *G. davidsonii* assemblies using BUSCO database. **Table S8**. Summary of 1-to-1 blocks between *G. thurberi* and *G. raimondii*, between *G. davidsonii* and *G. raimondii*, between *G. thurberi* and *G. davidsonii* or between *G. thurberi* and *G. turneri*. **Table S9**. Summary of gene duplication type information for the *G. thurberi* and *G. davidsonii* genomes. **Table S10**. The duplication gene type of transcription factors. **Table S11**. The centromeric regions identified by Hi-C heatmap combined the centromere specific CRGs mapping. **Table S12**. Comparison of CenLTR with the reported GhCR1-GhCR4. **Table S13**. Summary of Illumina sequencing clean data in *G. thurberi* and *G. davidsonii*. **Table S14**. Summary of accession information for mRNA-seq.

**Additional file 2: Figures S1-S31**.

## Data Availability

All the raw sequencing data for *G. thurberi* and *G. davidsonii* genome assembly data, mRNA-seq, and methylation are accessible through the NCBI under accession PRJNA659592. These supporting data (genome assemblies and genes annotations, as well as gff files for gene models) are available from the website (grand.cricaas.com.cn). Data supporting the findings of this work are available within the paper and its Supplementary Information files. The datasets generated and analyzed during the current study are available from the corresponding author on reasonable request.

## Abbreviations

TAD: Topologically associating domains
*G. raimondii*: *Gossypium raimondii*
*G. hirsutum*: *Gossypium hirsutum*
*G. barbadense*: *Gossypium barbadense*
*G. tomentosum*: *Gossypium tomentosum*
*G. mustelinum*: *Gossypium mustelinum*
*G. darwinii*: *Gossypium darwinii*
Hi-C: High-throughput chromosome conformation capture
CRG: Centromere Retroelement *Gossypium*
