# Supplementary material for: Cotton D genome assemblies built with long-read data unveil mechanisms of centromere evolution and stress tolerance divergence
Source: BMC Biol. 2021 Jun 3;19:115. doi: 10.1186/s12915-021-01041-0 (PMC8176745; doi:10.1186/s12915-021-01041-0)
Supplement: Supplementary file 1 — Additional file 1: Tables S1-S14. Table S1. Summary of Nanopore sequencing clean data in G. thurberi and G .davidsonii. Table S2. Nanopore sequencing reads length distribution in G. thurberi and G. davidsonii. Table S3. Genome assembly statistic information for G. thurberi and G. davidsonii. Table S4. Summary of Hi-C contact data mapped against the G. thurberi and G. davidsonii. Table S5. Comparison of repetitive elements between G. thurberi and G. davidsonii. Table S6. The Illumina short reads from G. thurberi and G. davidsonii mapping against G. thurberi and G. davidsonii assemblies respectively. Table S7. Evaluation of the G. thurberi and G. davidsonii assemblies using BUSCO database. Table S8. Summary of 1-to-1 blocks between G. thurberi and G. raimondii, between G. davidsonii and G. raimondii, between G. thurberi and G. davidsonii or between G. thurberi and G. turneri. Table S9. Summary of gene duplication type information for the G. thurberi and G. davidsonii genomes. Table S10. The duplication gene type of transcription factors. Table S11. The centromeric regions identified by Hi-C heatmap combined the centromere specific CRGs mapping. Table S12. Comparison of CenLTR with the reported GhCR1-GhCR4. Table S13. Summary of Illumina sequencing clean data in G. thurberi and G. davidsonii. Table S14. Summary of accession information for mRNA-seq. [file 12915_2021_1041_MOESM1_ESM.docx]

Table S1 Summary of Nanopore sequencing clean data in *G. thurberi* and *G.davidsonii*

| Species | Seq Number | Total length | N50 | N90 | Average length | Max length | Mean Quality |
| --- | --- | --- | --- | --- | --- | --- | --- |
| *G. thurberi* | 3,575,506 | 114,287,348,957 | 42,915 | 18,986 | 31,963 | 431,233 | 8.33 |
| *G. davidsonii* | 3,237,739 | 108,304,182,926 | 43,855 | 20,294 | 33,450 | 409,570 | 8.35 |

Table S2 Nanopore sequencing reads length distribution in *G. thurberi* and *G. davidsonii*

|  | *G. thurberi* (D_1_) | | | | *G. davidsonii* (D_3_) | | | |
| --- | --- | --- | --- | --- | --- | --- | --- | --- |
| Length | Reads Number | Length (bp) | Ratio (%) | Average Length (bp) | Reads Number | Length (bp) | Ratio (%) | Average Length (bp) |
| 2000~5000 | 255,294 | 865,426,424 | 0.75 | 3,389.92 | 189,713 | 643,339,246 | 0.59 | 3,391.11 |
| 5000~10000 | 273,471 | 2,018,458,834 | 1.76 | 7,380.88 | 230,560 | 1,719,973,428 | 1.58 | 7,459.98 |
| 10000~20000 | 655,079 | 10,316,095,225 | 9.02 | 15,747.86 | 512,202 | 8,004,706,615 | 7.39 | 15,628.02 |
| 20000~30000 | 789,997 | 19,532,590,900 | 17.09 | 24,724.89 | 737,535 | 18,285,956,491 | 16.88 | 24,793.34 |
| 30000~40000 | 549,780 | 19,083,721,096 | 16.69 | 34,711.55 | 536,955 | 18,650,938,529 | 17.22 | 34,734.63 |
| 40000~50000 | 388,673 | 17,369,171,100 | 15.19 | 44,688.39 | 382,607 | 17,104,157,061 | 15.79 | 44,704.24 |
| 50000~60000 | 263,475 | 14,402,069,259 | 12.60 | 54,661.99 | 260,414 | 14,234,810,004 | 13.14 | 54,662.23 |
| 60000~70000 | 170,302 | 11,002,267,178 | 9.62 | 64,604.45 | 167,261 | 10,807,581,903 | 9.97 | 64,615.07 |
| 70000~80000 | 103,755 | 7,735,967,416 | 6.76 | 74,559.94 | 100,914 | 7,523,206,144 | 6.94 | 74,550.66 |
| >=80000 | 125,680 | 11,961,581,525 | 10.46 | 95,174.90 | 119,578 | 11,329,513,505 | 10.46 | 94,745.80 |

Table S3 Genome assembly statistic information for *G. thurberi* and *G. davidsonii*

|  | *G. thurberi* | |  | *G. davidsonii* | |  | *G. turneri* | |  | *G. raimondii* (JGI) | |  | *G. raimondii* (ISU) | |
| --- | --- | --- | --- | --- | --- | --- | --- | --- | --- | --- | --- | --- | --- | --- |
|  | Size (bp) | Numbers |  | Size (bp) | Numbers |  | Size (bp) | Numbers |  | Size (bp) | Numbers |  | Size (bp) | Numbers |
| N10 | 44,248,292 | 2 |  | 40,375,797 | 2 |  | 17471127 | 4 |  | 380,608 | 148 |  | 12,986,642 | 5 |
| N20 | 34,239,230 | 4 |  | 37,290,725 | 4 |  | 13503268 | 9 |  | 269,816 | 386 |  | 11,324,014 | 11 |
| N30 | 32,405,820 | 6 |  | 33,849,046 | 7 |  | 11127545 | 15 |  | 209,739 | 704 |  | 9,366,740 | 18 |
| N40 | 27,158,635 | 9 |  | 28,892,445 | 9 |  | 9129568 | 23 |  | 171,828 | 1101 |  | 7,264,449 | 27 |
| N50 | 24,700,000 | 12 |  | 26,767,914 | 12 |  | 7909293 | 32 |  | 135,582 | 1591 |  | 6,291,832 | 38 |
| N60 | 23,438,811 | 15 |  | 23,724,233 | 15 |  | 6155871 | 43 |  | 106,546 | 2215 |  | 5,284,150 | 51 |
| N70 | 20,053,490 | 19 |  | 21,064,958 | 19 |  | 4182627 | 58 |  | 80,598 | 3019 |  | 3,769,173 | 67 |
| N80 | 16,387,335 | 23 |  | 16,156,749 | 23 |  | 2621252 | 80 |  | 55,831 | 4132 |  | 3,025,574 | 88 |
| N90 | 8,178,790 | 30 |  | 9,822,526 | 30 |  | 1624019 | 117 |  | 29,657 | 5931 |  | 2,044,991 | 118 |
| Total number |  | 74 |  |  | 104 |  |  | 220 |  |  | 19735 |  |  | 187 |
| Anchored and oriented contigs |  | 63 |  |  | 90 |  |  | 220 |  |  | 16924 |  |  | 187 |
| Total Size (Mb) | 779.6 |  |  | 801.2 |  |  | 755.2 |  |  | 748 |  |  | 734.8 |  |

Table S4 Summary of Hi-C contact data mapped against the *G. thruberi* and *G. davidsonii*

| Mapping Type | *G. thurberi* | |  | *G. davidsonii* | |
| --- | --- | --- | --- | --- | --- |
|  | Number | Ratio（%） |  | Number | Ratio（%） |
| Total Read Pairs | 504,092,582 | 100 |  | 653,648,379 | 100.00 |
| Mapped Reads | 961,054,073 | 95.33 |  | 1,223,038,383 | 93.55 |
| Unique Mapped Read Pairs | 357,446,269 | 70.91 |  | 457,625,506 | 70.01 |
| Valid Interaction Pairs | 284,315,160 | 56.40 |  | 280,326,543 | 42.89 |
| Dangling End Pairs | 43,780,096 | 8.68 |  | 95,280,035 | 14.58 |
| Re-ligation Pairs | 2,517,938 | 0.50 |  | 6,264,974 | 0.96 |
| Self-cycle Pairs | 15,314,570 | 3.04 |  | 56,311,897 | 8.62 |
| Dumped Pairs | 11,518,505 | 2.28 |  | 19,442,057 | 2.97 |

Table S5 Comparison of repetitive elements between *G. thurberi* and *G. davidsonii*

| Type | *G. thurberi* | | |  | *G. daviadsonii* | | |
| --- | --- | --- | --- | --- | --- | --- | --- |
|  | Number | Length （bp） | Ratio(%) |  | Number | Length (bp) | Ratio(%) |
| ClassI | 516,494 | 389,458,271 | 49.96 |  | 543513 | 406,588,855 | 50.74 |
| ClassI/DIRS | 23,449 | 19,044,887 | 2.44 |  | 23855 | 20,821,341 | 2.6 |
| ClassI/LINE | 23,012 | 10,939,924 | 1.4 |  | 23519 | 9,978,435 | 1.25 |
| ClassI/LTR | 1,058 | 766,503 | 0.1 |  | 2317 | 1,111,316 | 0.14 |
| ClassI/LTR/Copia | 109,241 | 66,940,493 | 8.59 |  | 114149 | 69,281,901 | 8.65 |
| ClassI/LTR/Gypsy | 233,348 | 247,171,449 | 31.7 |  | 243569 | 257,999,601 | 32.2 |
| ClassI/PLE\|LARD | 122,450 | 43,577,700 | 5.59 |  | 132314 | 45,730,736 | 5.71 |
| ClassI/SINE | 1,754 | 319,936 | 0.04 |  | 2400 | 596,510 | 0.07 |
| ClassI/TRIM | 1,430 | 530,444 | 0.07 |  | 1156 | 1,018,287 | 0.13 |
| ClassI/Unknown | 752 | 166,935 | 0.02 |  | 234 | 50,853 | 0.01 |
| ClassII | 54,051 | 26,971,475 | 3.46 |  | 58877 | 28,498,717 | 3.56 |
| ClassII/Crypton | 6 | 326 | 0 |  | 8 | 421 | 0 |
| ClassII/Helitron | 13,834 | 4,397,118 | 0.56 |  | 16943 | 5,428,849 | 0.68 |
| ClassII/MITE | 3,134 | 855,376 | 0.11 |  | 4325 | 1,325,977 | 0.17 |
| ClassII/Maverick | 218 | 152,322 | 0.02 |  | 2 | 90 | 0 |
| ClassII/TIR | 35,105 | 21,161,045 | 2.71 |  | 36143 | 21,384,331 | 2.67 |
| ClassII/Unknown | 1,754 | 405,288 | 0.05 |  | 1456 | 359,049 | 0.04 |
| PotentialHostGene | 19,445 | 4,980,268 | 0.64 |  | 16844 | 4,345,886 | 0.54 |
| SSR | 4,418 | 950,397 | 0.12 |  | 2483 | 505,755 | 0.06 |
| Unknown | 93,563 | 29,487,020 | 3.78 |  | 96372 | 29,453,394 | 3.68 |
| Total | 594,408 | 451,847,348 | 57.96 |  | 621717 | 469,392,391 | 58.58 |

Table S6 The Illumina short reads from *G. thurberi* and *G. davidsonii* mapping against *G. thurberi and G. davidsonii* assemblies respectively

| Species | Total_reads | Mapped_reads | Mapped (%) | Properly mapped | Properly mapped(%) |
| --- | --- | --- | --- | --- | --- |
| *G. thurberi* | 192,621,900 | 192,051,468 | 99.70% | 183,849,088 | 95.86% |
| *G.davidsonii* | 212,585,232 | 212,020,100 | 99.73% | 203,670,590 | 96.16% |

Table S7 Evaluation of the *G. thurberi* and *G. davidsonii* assemblies using BUSCO database

| Species | Complete BUSCOs | Complete and single-copy BUSCOs | Complete and duplicated BUSCOs | Fragmented BUSCOs | Missing BUSCOs |
| --- | --- | --- | --- | --- | --- |
| *G. thurberi* | 1372 (95.28%) | 1223 (84.93%) | 149 (10.35%) | 18 (1.25%) | 50 (3.47%) |
| *G. davidsonii* | 1374 (95.42%) | 1223 (85.62%) | 141 (9.79%) | 14 (0.97%) | 52 (3.61%) |

Table S8 Summary of 1-to-1 blocks between *G. thurberi* and *G. raimondii*, between *G. davidsonii* and *G. raimondii, between G. thurberi and G. davidsonii or between G. thurberi and G. turneri*

|  | *G.thurberi*_Vs_*G.davidsonii* | |  | *G. thurberi*_Vs_*G. raimondii* | |  | *G.davidsonii*_Vs_*G.raimondii* | |  | *G.thurberi_*Vs*_G.turneri* | |
| --- | --- | --- | --- | --- | --- | --- | --- | --- | --- | --- | --- |
|  | *G.thurberi* | *G.davidsonii* |  | *G. thurberi* | *G. raimondii* |  | *G. davidsonii* | *G. raimondii* |  | *G.thurberi* | *G.turneri* |
| 1-to-1 | 111,405 | 111,405 |  | 116,868 | 116,868 |  | 110,609 | 110,609 |  | 116,375 | 116,375 |
| Total Length (bp) | 627,325,551 | 626,826,221 |  | 607,110,769 | 606,554,161 |  | 618,663,186 | 617,596,744 |  | 586,742,752 | 587,838,795 |
| Ratio (%) | 78.5 | 80.64 |  | 77.87 | 81.64 |  | 77.21 | 83.13 |  | 75.49 | 77.84 |
| AvgLength (bp) | 5631.04 | 5626.55 |  | 5194.8 | 5190.1 |  | 5593.2 | 5583.6 |  | 5041.83 | 5051.25 |
| AvgIdentity (%) | 95.32 | 95.32 |  | 95.26 | 95.26 |  | 95.18 | 95.18 |  | 95.03 | 95.03 |

Table S9 Summary of gene duplication type information for the *G. thurberi* and *G. davidsonii* genomes. WGD represents whole genome duplication. SD represents segmental duplication.

| Duplication type | *G. thurberi* | *G. davidsonii* |
| --- | --- | --- |
| Singleton | 3,136 | 3,154 |
| Dispersed | 8,978 | 8,931 |
| Proximal | 1,281 | 1,328 |
| Tandem | 3,456 | 3,493 |
| WGD or SD | 24,465 | 24,565 |

Table S10 The duplication gene type of transcription factors

| Duplication type | *G. thurberi* | *G. davidsonii* |
| --- | --- | --- |
| Singleton | 20 | 19 |
| Dispersed | 351 | 319 |
| Proximal | 69 | 73 |
| Tandem | 190 | 169 |
| WGD or SD | 2,045 | 2,049 |

|  | *G. thurberi* | | | *G. davidsonii* | | |
| --- | --- | --- | --- | --- | --- | --- |
| Chromosome | Start | End | Length | Start | End | Length |
| Chr01 | 39250000 | 40350000 | 1,100,000 | 38600000 | 39850000 | 1,250,000 |
| Chr02 | 33850000 | 34950000 | 1,100,000 | 33850000 | 35150000 | 1,300,000 |
| Chr03 | 23450000 | 24450000 | 1,000,000 | 23600000 | 25300000 | 1,700,000 |
| Chr04 | 24950000 | 25450000 | 500,000 | 25900000 | 27200000 | 1,300,000 |
| Chr05 | 30900000 | 31450000 | 550,000 | 30850000 | 32350000 | 1,500,000 |
| Chr06 | 17500000 | 18550000 | 1,050,000 | 18300000 | 19600000 | 1,300,000 |
| Chr07 | 42350000 | 43400000 | 1,050,000 | 42150000 | 43350000 | 1,200,000 |
| Chr08 | 23650000 | 24400000 | 750,000 | 24650000 | 26100000 | 1,450,000 |
| Chr09 | 38250000 | 39600000 | 1,350,000 | 40150000 | 41850000 | 1,700,000 |
| Chr10 | 33700000 | 34700000 | 1,000,000 | 34550000 | 35700000 | 1,150,000 |
| Chr11 | 28200000 | 29150000 | 950,000 | 37000000 | 38800000 | 1,800,000 |
| Chr12 | 26750000 | 28250000 | 1,500,000 | 24150000 | 25600000 | 1,450,000 |
| Chr13 | 26900000 | 27900000 | 1,000,000 | 27900000 | 28950000 | 1,050,000 |

Table S11 The centromeric regions identified by Hi-C heatmap combined the centromere specific CRGs mapping

Table S12 Comparison of CenLTR with the reported GhCR1-GhCR4

|  | Chr. | Strand | Location | Size | 5'-LTR | | 3'-LTR | | LTR region similarity |
| --- | --- | --- | --- | --- | --- | --- | --- | --- | --- |
|  |  |  |  |  | Location | Size (bp) | Location | Size (bp) |  |
| CenLTR | Chr12 | - | 26,765,415-26,783,753 | 18,839 | 26,780,293-26,783,753 | 3461 | 26,765,415-26,768,869 | 3455 | 0.945 |
| GhCR1 | BAC97G20 | + | 2469-8126 | 5658 | 2469-3916 | 1448 | 6674-8126 | 1453 | 0.986 |
| GhCR2 | BAC97G20 | + | 25,391-33534 | 8144 | 25,391-26,891 | 1501 | 32,022-33,534 | 1503 | 0.994 |
| GhCR3 | BAC97G20 | + | 73,857-79229 | 16,075 | 50,180-50518 | 339 | 65,916-66,254 | 339 | 0.985 |
| GhCR4 | BAC97G20 | - | 73,857-74,729 | 5373 | 73,857-74,729 | 873 | 78,356-74,729 | 874 | 0.992 |

| Samples | Read-pair number | Size (Gb) | Samples | Read-pair number | Size (Gb) |
| --- | --- | --- | --- | --- | --- |
| D3-CK-3-R1 | 25,682,990 | 7.7 | D5-CK-3-R1 | 20,535,242 | 6.2 |
| D3-CK-3-R2 | 24,867,285 | 7.5 | D5-CK-3-R2 | 20,289,625 | 6.1 |
| D3-CK-3-R3 | 22,577,374 | 6.8 | D5-CK-3-R3 | 22,274,198 | 6.7 |
| D3-CK-6-R1 | 23,193,320 | 7.0 | D5-CK-6-R1 | 19,477,172 | 5.8 |
| D3-CK-6-R2 | 23,854,580 | 7.2 | D5-CK-6-R2 | 26,393,107 | 7.9 |
| D3-CK-6-R3 | 21,059,552 | 6.3 | D5-CK-6-R3 | 19,556,159 | 5.9 |
| D3-CK-24-R1 | 23,228,055 | 7.0 | D5-CK-24-R1 | 20,230,853 | 6.1 |
| D3-CK-24-R2 | 20,277,798 | 6.1 | D5-CK-24-R2 | 21,401,958 | 6.4 |
| D3-CK-24-R3 | 19,336,153 | 5.8 | D5-CK-24-R3 | 24,157,007 | 7.2 |
| D3-NACL-3-R1 | 19,163,387 | 5.7 | D5-NACL-3-R1 | 22,597,154 | 6.8 |
| D3-NACL-3-R2 | 18,808,643 | 5.6 | D5-NACL-3-R2 | 20,497,346 | 6.1 |
| D3-NACL-3-R3 | 19,543,722 | 5.9 | D5-NACL-3-R3 | 19,973,702 | 6.0 |
| D3-NACL-6-R1 | 20,614,281 | 6.2 | D5-NACL-6-R1 | 20,321,509 | 6.1 |
| D3-NACL-6-R2 | 19,807,566 | 5.9 | D5-NACL-6-R2 | 19,382,843 | 5.8 |
| D3-NACL-6-R3 | 22,653,492 | 6.8 | D5-NACL-6-R3 | 20,341,975 | 6.1 |
| D3-NACL-24-R1 | 25,909,835 | 7.8 | D5-NACL-24-R1 | 24,341,542 | 7.3 |
| D3-NACL-24-R2 | 18,815,657 | 5.6 | D5-NACL-24-R2 | 21,728,082 | 6.5 |
| D3-NACL-24-R3 | 24,526,676 | 7.4 | D5-NACL-24-R3 | 26,052,182 | 7.8 |

Table S13 Summary of Illumina sequencing clean data in *G. thurberi* and *G. davidsonii.*

Table S14 Summary of accession information for mRNA-seq*.* The data have been deposited in NCBI SRA

| Accession | Organism | Treatments |
| --- | --- | --- |
| SAMN15916026 | *G. davidsonii* | Replicate 1 of Control at 24 hours post treatment |
| SAMN15916027 | *G. davidsonii* | Replicate 2 of Control at 24 hours post treatment |
| SAMN15916028 | *G. davidsonii* | Replicate 3 of Control at 24 hours post treatment |
| SAMN15916029 | *G. davidsonii* | Replicate 1 of Control at 3 hours post treatment |
| SAMN15916030 | *G. davidsonii* | Replicate 2 of Control at3 hours post treatment |
| SAMN15916031 | *G. davidsonii* | Replicate 3 of Control at 3 hours post treatment |
| SAMN15916032 | *G. davidsonii* | Replicate 1 of Control at 6 hours post treatment |
| SAMN15916033 | *G. davidsonii* | Replicate 2 of Control at 6 hours post treatment |
| SAMN15916034 | *G. davidsonii* | Replicate 3 of Control at 6 hours post treatment |
| SAMN15916038 | *G. davidsonii* | Replicate 1 of NaCl treatment at 24 hours post treatment |
| SAMN15916039 | *G. davidsonii* | Replicate 2 of NaCl treatment at 24 hours post treatment |
| SAMN15916040 | *G. davidsonii* | Replicate 3 of NaCl treatment at 24 hours post treatment |
| SAMN15916041 | *G. davidsonii* | Replicate 1 of NaCl treatment at 3 hours post treatment |
| SAMN15916042 | *G. davidsonii* | Replicate 2 of NaCl treatment at3 hours post treatment |
| SAMN15916043 | *G. davidsonii* | Replicate 3 of NaCl treatment at 3 hours post treatment |
| SAMN15916044 | *G. davidsonii* | Replicate 1 of NaCl treatment at 6 hours post treatment |
| SAMN15916045 | *G. davidsonii* | Replicate 2 of NaCl treatment at 6 hours post treatment |
| SAMN15916046 | *G. davidsonii* | Replicate 3 of NaCl treatment at 6 hours post treatment |
| SAMN15916050 | *G. raimondii* | Replicate 1 of Control at 24 hours post treatment |
| SAMN15916051 | *G. raimondii* | Replicate 2 of Control at 24 hours post treatment |
| SAMN15916052 | *G. raimondii* | Replicate 3 of Control at 24 hours post treatment |
| SAMN15916053 | *G. raimondii* | Replicate 1 of Control at 3 hours post treatment |
| SAMN15916054 | *G. raimondii* | Replicate 2 of Control at3 hours post treatment |
| SAMN15916055 | *G. raimondii* | Replicate 3 of Control at 3 hours post treatment |
| SAMN15916056 | *G. raimondii* | Replicate 1 of Control at 6 hours post treatment |
| SAMN15916057 | *G. raimondii* | Replicate 2 of Control at 6 hours post treatment |
| SAMN15916058 | *G. raimondii* | Replicate 3 of Control at 6 hours post treatment |
| SAMN15916062 | *G. raimondii* | Replicate 1 of NaCl treatment at 24 hours post treatment |
| SAMN15916063 | *G. raimondii* | Replicate 2 of NaCl treatment at 24 hours post treatment |
| SAMN15916064 | *G. raimondii* | Replicate 3 of NaCl treatment at 24 hours post treatment |
| SAMN15916065 | *G. raimondii* | Replicate 1 of NaCl treatment at 3 hours post treatment |
| SAMN15916066 | *G. raimondii* | Replicate 2 of NaCl treatment at3 hours post treatment |
| SAMN15916067 | *G. raimondii* | Replicate 3 of NaCl treatment at 3 hours post treatment |
| SAMN15916068 | *G. raimondii* | Replicate 1 of NaCl treatment at 6 hours post treatment |
| SAMN15916069 | *G. raimondii* | Replicate 2 of NaCl treatment at 6 hours post treatment |
| SAMN15916070 | *G. raimondii* | Replicate 3 of NaCl treatment at 6 hours post treatment |
| [SRR8267556](https://trace.ncbi.nlm.nih.gov/Traces/sra?run=SRR8267556) | *G. raimondii* | Replicate 1 of Verticillium dahliae treatment at 0 hpi |
| [SRR8267557](https://trace.ncbi.nlm.nih.gov/Traces/sra?run=SRR8267557) | *G. raimondii* | Replicate 2 of Verticillium dahliae treatment at 0 hpi |
| [SRR8267558](https://trace.ncbi.nlm.nih.gov/Traces/sra?run=SRR8267558) | *G. raimondii* | Replicate 3 of Verticillium dahliae treatment at 0 hpi |
| [SRR8267565](https://trace.ncbi.nlm.nih.gov/Traces/sra?run=SRR8267565) | *G. raimondii* | Replicate 1 of Verticillium dahliae treatment at 12 hpi |
| [SRR8267566](https://trace.ncbi.nlm.nih.gov/Traces/sra?run=SRR8267566) | *G. raimondii* | Replicate 2 of Verticillium dahliae treatment at 12 hpi |
| [SRR8267567](https://trace.ncbi.nlm.nih.gov/Traces/sra?run=SRR8267567) | *G. raimondii* | Replicate 3 of Verticillium dahliae treatment at 12 hpi |
| [SRR8267574](https://trace.ncbi.nlm.nih.gov/Traces/sra?run=SRR8267574) | *G. raimondii* | Replicate 1 of Verticillium dahliae treatment at 48 hpi |
| [SRR8267575](https://trace.ncbi.nlm.nih.gov/Traces/sra?run=SRR8267575) | *G. raimondii* | Replicate 2 of Verticillium dahliae treatment at 48 hpi |
| [SRR8267576](https://trace.ncbi.nlm.nih.gov/Traces/sra?run=SRR8267576) | *G. raimondii* | Replicate 3 of Verticillium dahliae treatment at 48 hpi |
| [SRR8267610](https://trace.ncbi.nlm.nih.gov/Traces/sra?run=SRR8267610) | *G. thurberi* | Replicate 1 of Verticillium dahliae treatment at 0 hpi |
| [SRR8267611](https://trace.ncbi.nlm.nih.gov/Traces/sra?run=SRR8267611) | *G. thurberi* | Replicate 2 of Verticillium dahliae treatment at 0 hpi |
| [SRR8267612](https://trace.ncbi.nlm.nih.gov/Traces/sra?run=SRR8267612) | *G. thurberi* | Replicate 3 of Verticillium dahliae treatment at 0 hpi |
| [SRR8267619](https://trace.ncbi.nlm.nih.gov/Traces/sra?run=SRR8267619) | *G. thurberi* | Replicate 1 of Verticillium dahliae treatment at 12 hpi |
| [SRR8267620](https://trace.ncbi.nlm.nih.gov/Traces/sra?run=SRR8267620) | *G. thurberi* | Replicate 2 of Verticillium dahliae treatment at 12 hpi |
| [SRR8267621](https://trace.ncbi.nlm.nih.gov/Traces/sra?run=SRR8267621) | *G. thurberi* | Replicate 3 of Verticillium dahliae treatment at 12 hpi |
| [SRR8267628](https://trace.ncbi.nlm.nih.gov/Traces/sra?run=SRR8267628) | *G. thurberi* | Replicate 1 of Verticillium dahliae treatment at 48 hpi |
| [SRR8267629](https://trace.ncbi.nlm.nih.gov/Traces/sra?run=SRR8267629) | *G. thurberi* | Replicate 2 of Verticillium dahliae treatment at 48 hpi |
| [SRR8267630](https://trace.ncbi.nlm.nih.gov/Traces/sra?run=SRR8267630) | *G. thurberi* | Replicate 3 of Verticillium dahliae treatment at 48 hpi |
