# Supplementary material for: Cotton D genome assemblies built with long-read data unveil mechanisms of centromere evolution and stress tolerance divergence
Source: BMC Biol. 2021 Jun 3;19:115. doi: 10.1186/s12915-021-01041-0 (PMC8176745; doi:10.1186/s12915-021-01041-0)
Supplement: Supplementary file 2 — Additional file 2: Figures S1-S31. [file 12915_2021_1041_MOESM2_ESM.docx]

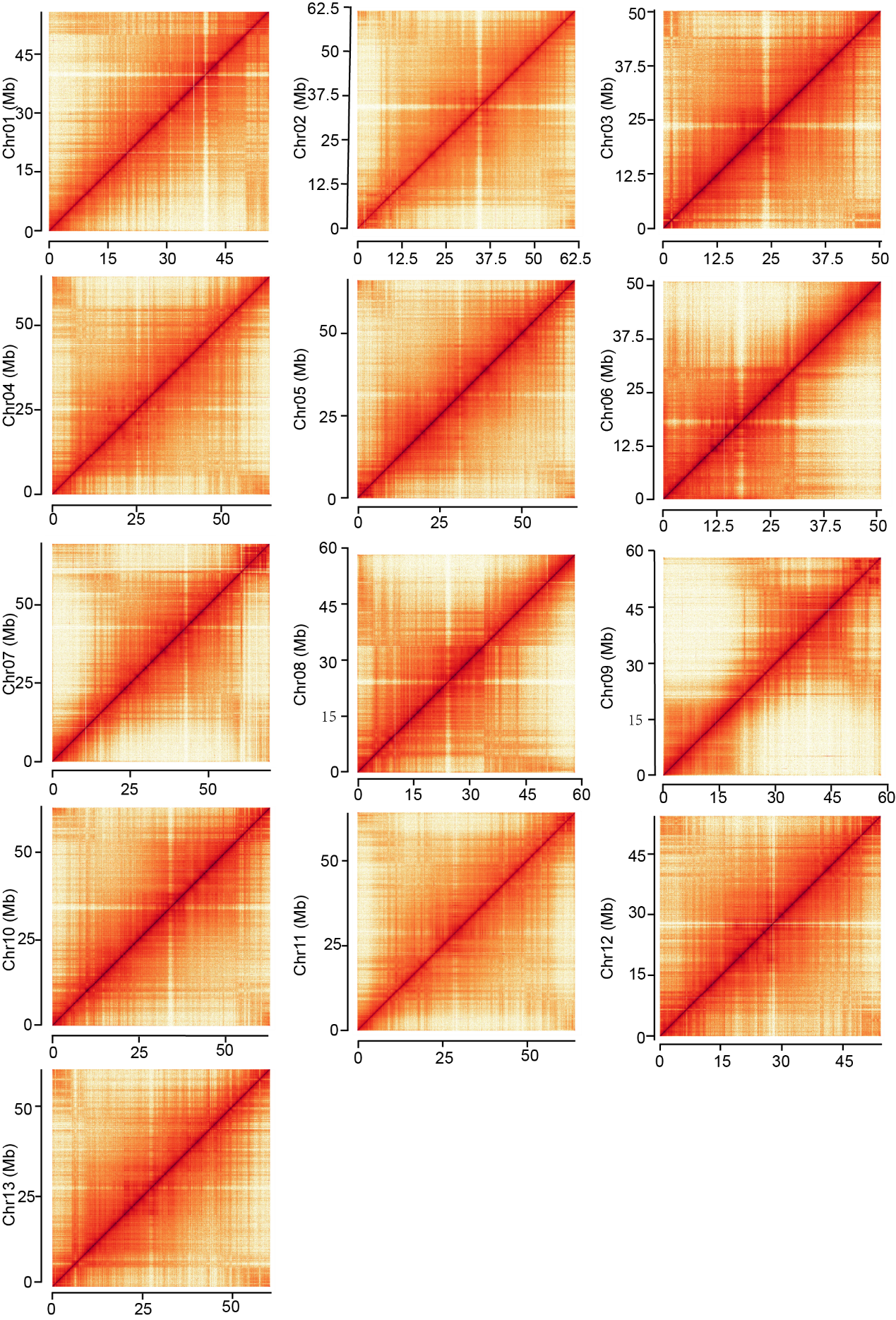


Figure S1 *G. thurberi* Hi-C contact data mapping on the *G. thurberi* genome. The diagonal regions showed strong contact signals, indicating that the contigs were accurately oriented on the pseudochromosomes.


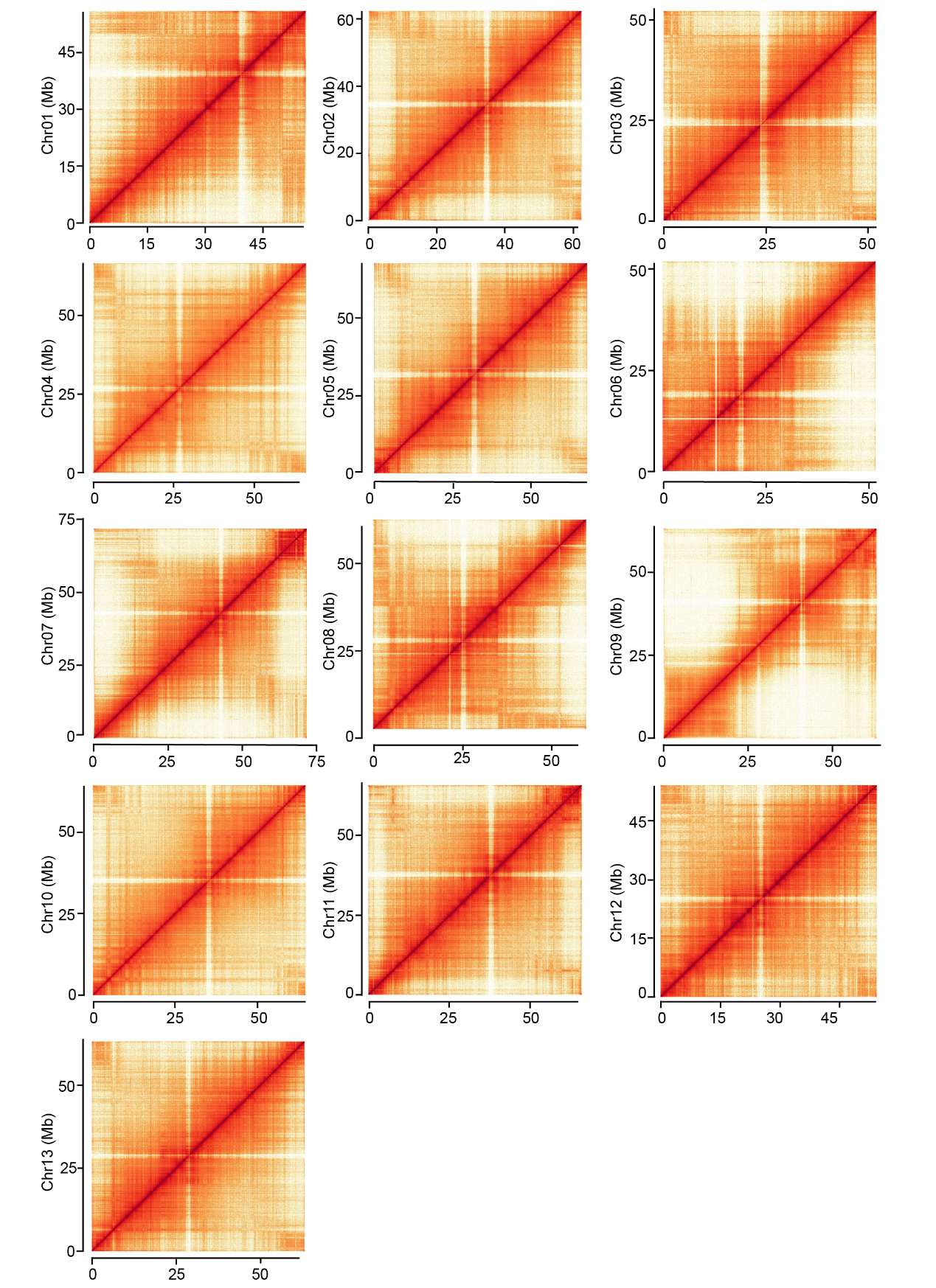


Figure S2 *G. davidsonii* Hi-C contact data mapping on the *G. davidsonii* genome. The diagonal regions showed strong contact signals, indicating that the contigs were accurately oriented on the pseudochromosomes.


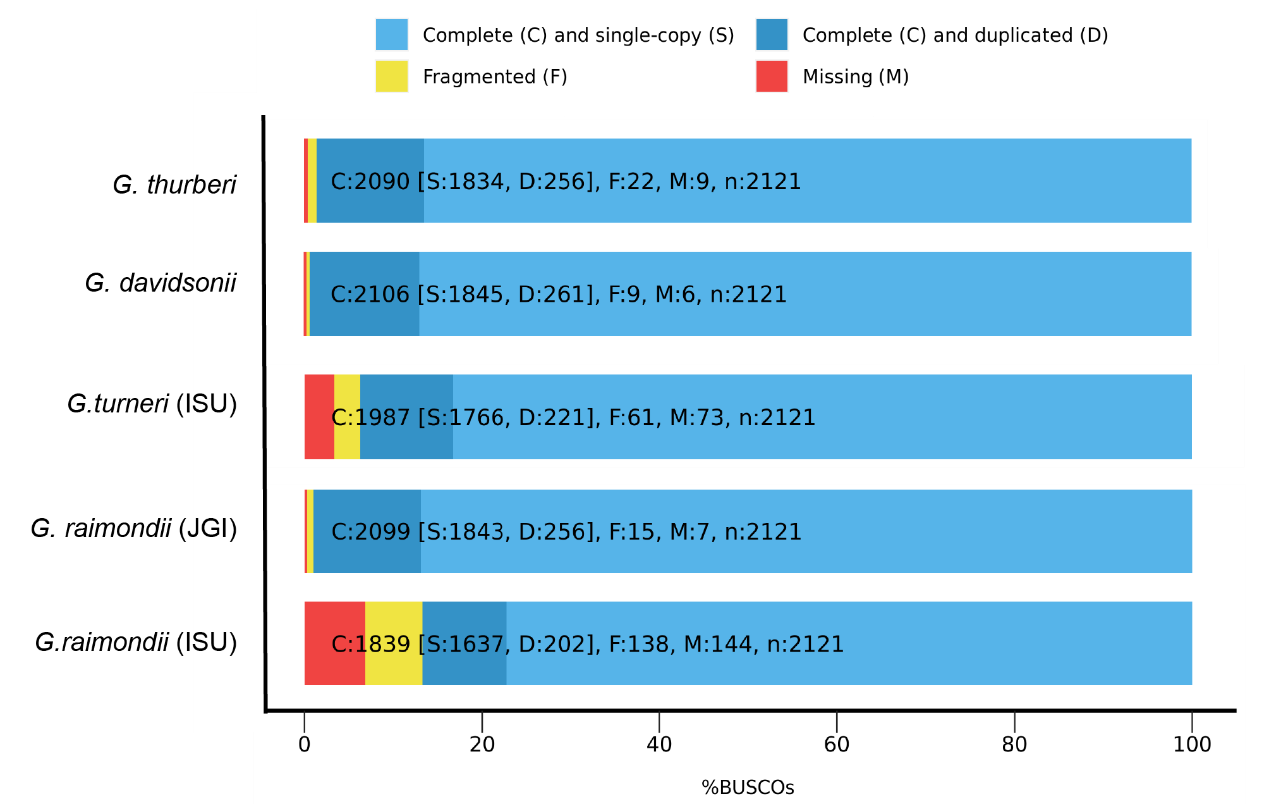


Figure S3 Evaluation the gene completeness in *G. thurberi*, *G. davidsonii,* *G. turner*i, and *G. raimondii* by mapping the protein sequences against 2121 conserved genes in the eudicotyledons_odb10 database. C, S, D, F, M represent complete, complete and single-copy, complete and duplicated, fragmented and missing BUSCOs.


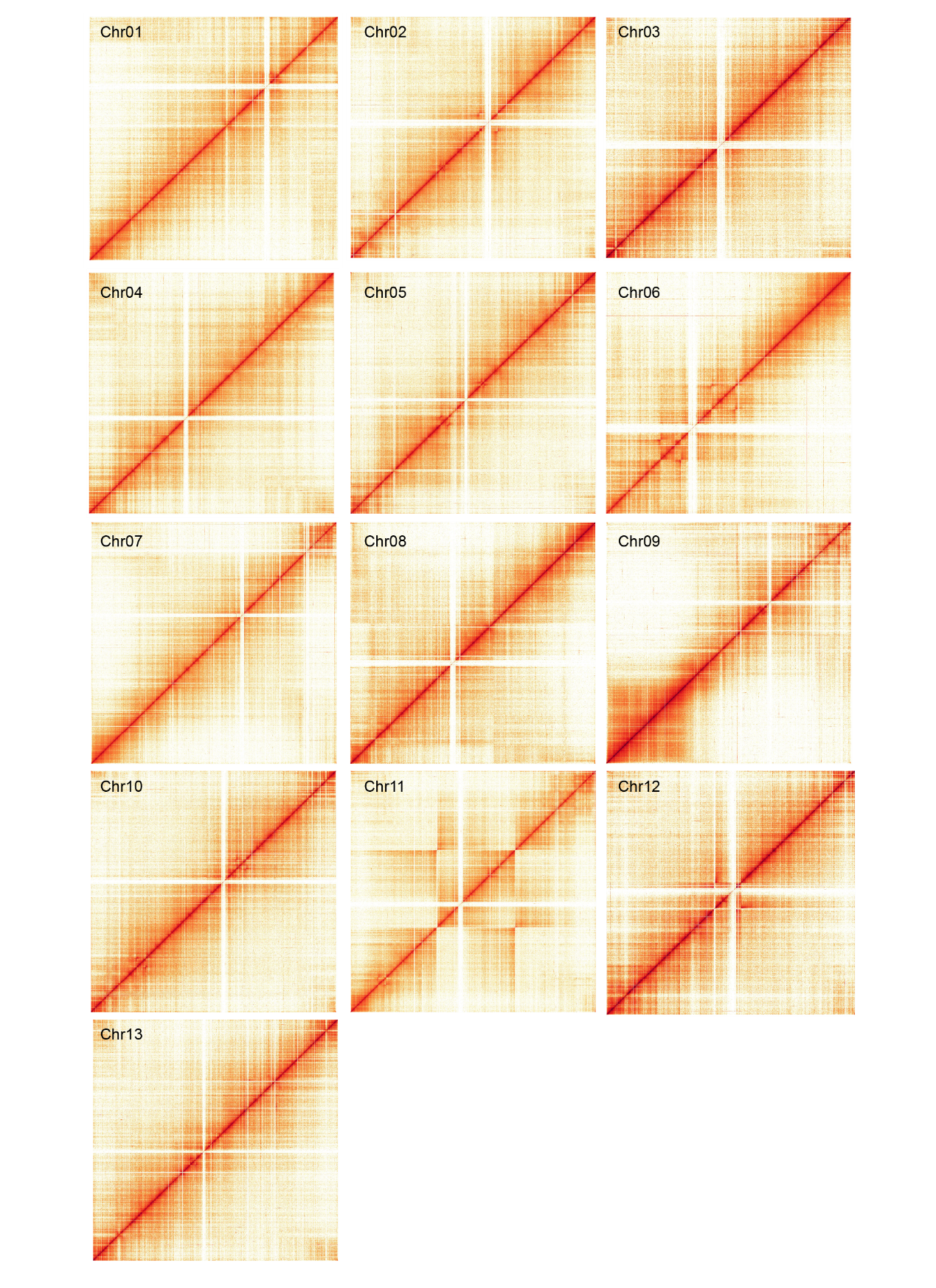


Figure S4 *G. davidsonii* Hi-C contact data mapping on the *G. thurberi* genome. The discontinuous signals are observed in Chr01, Chr02, Chr05, Chr06, Chr10, Chr11, these regions are inverted.


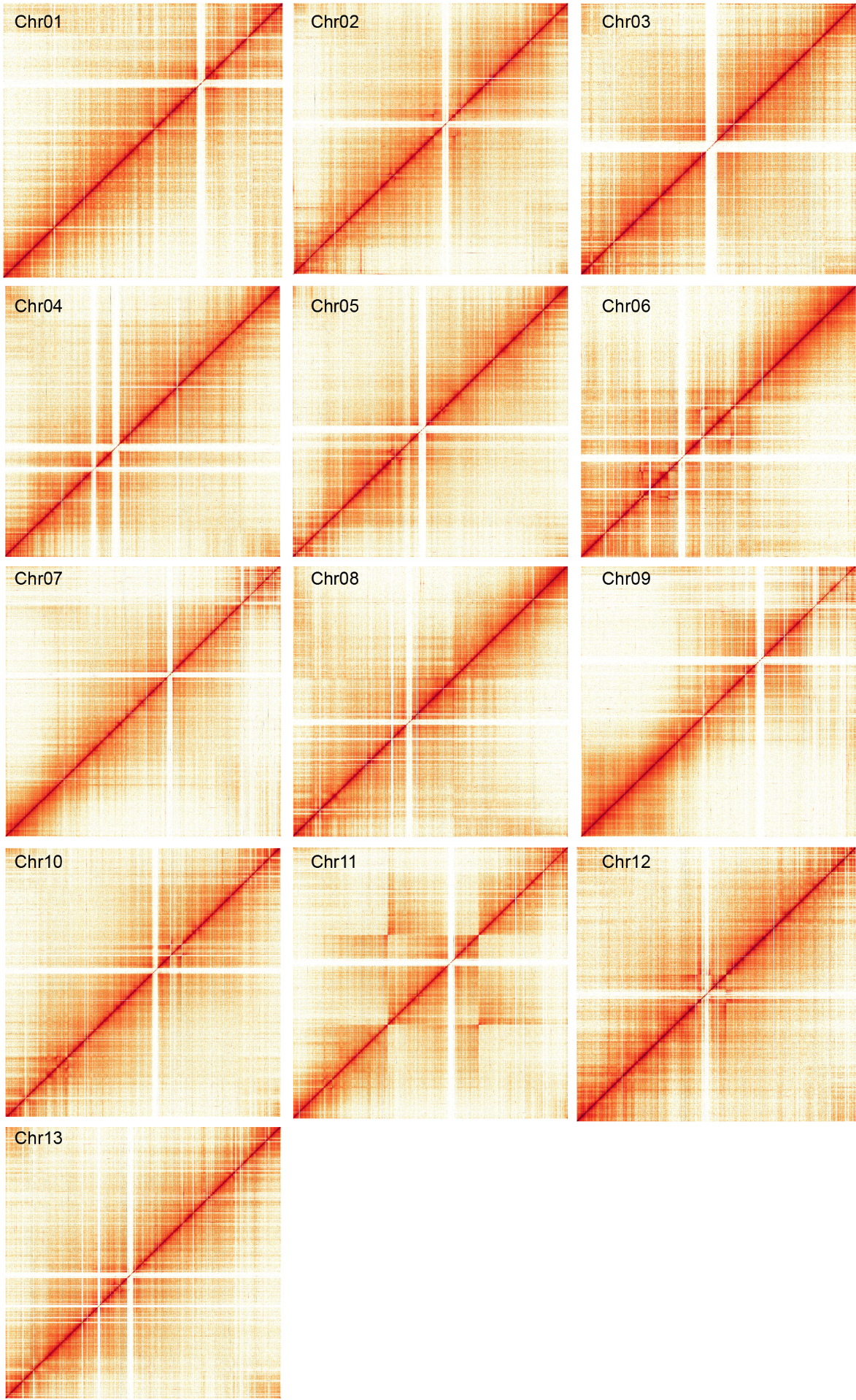


Figure S5 *G. thurberi* Hi-C contact data mapping on the *G. davidsonii* genome. The discontinuous signals are observed in Chr01, Chr02, Chr05, Chr06, Chr10, Chr11, these regions are inverted.


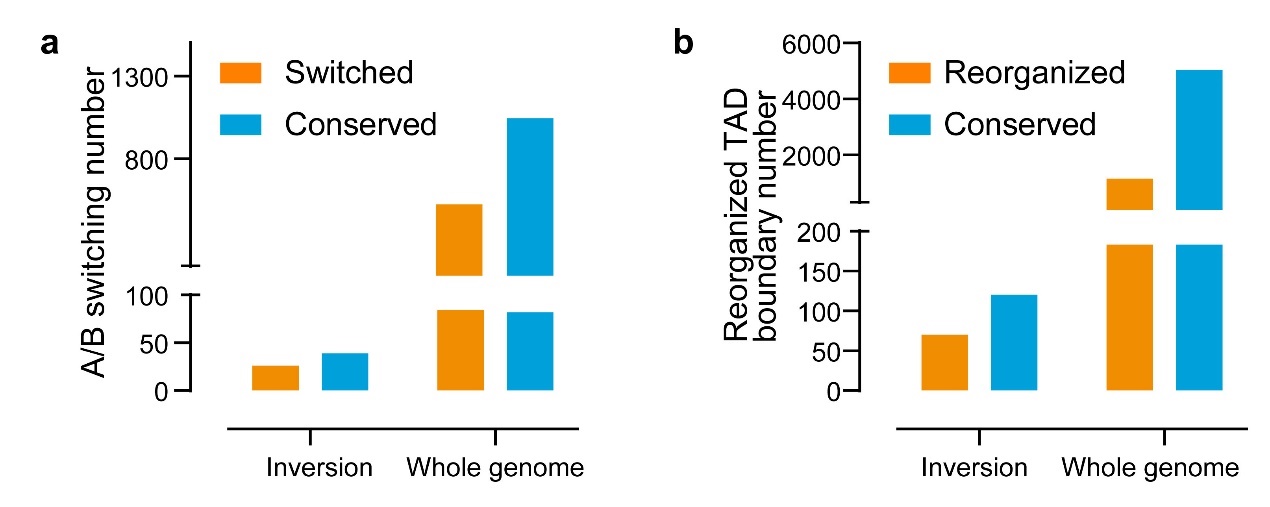


Figure S6 A/B compartments and TAD boundaries near the breakpoint. a, Switched and conserved A/B compartments near the breakpoint and whole genome levels. b，Reorganized and conserved TAD boundaries near the breakpoint.


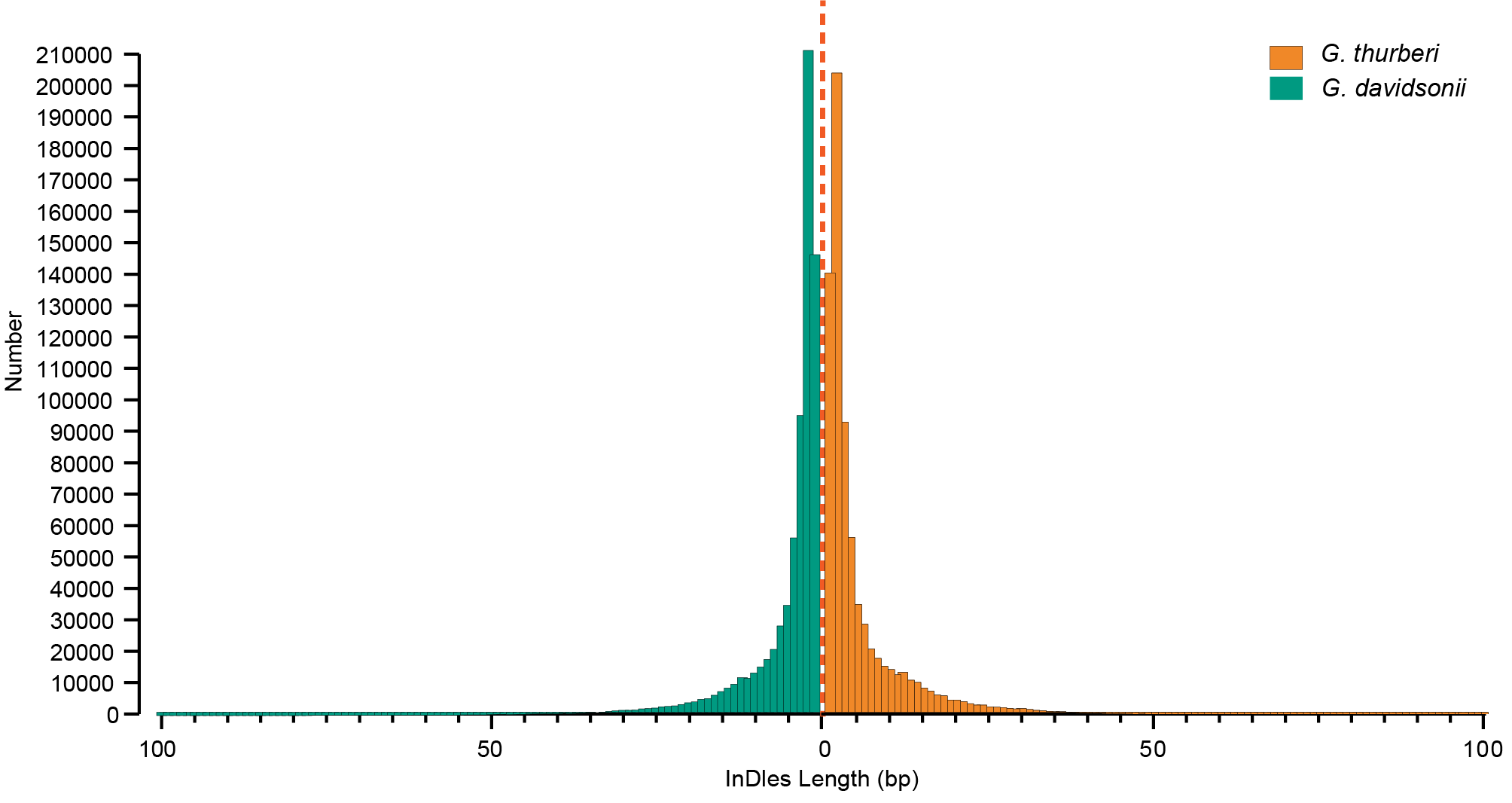


Figure S7 Indels length distribution in *G. thurberi* and *G. davidsonii*.


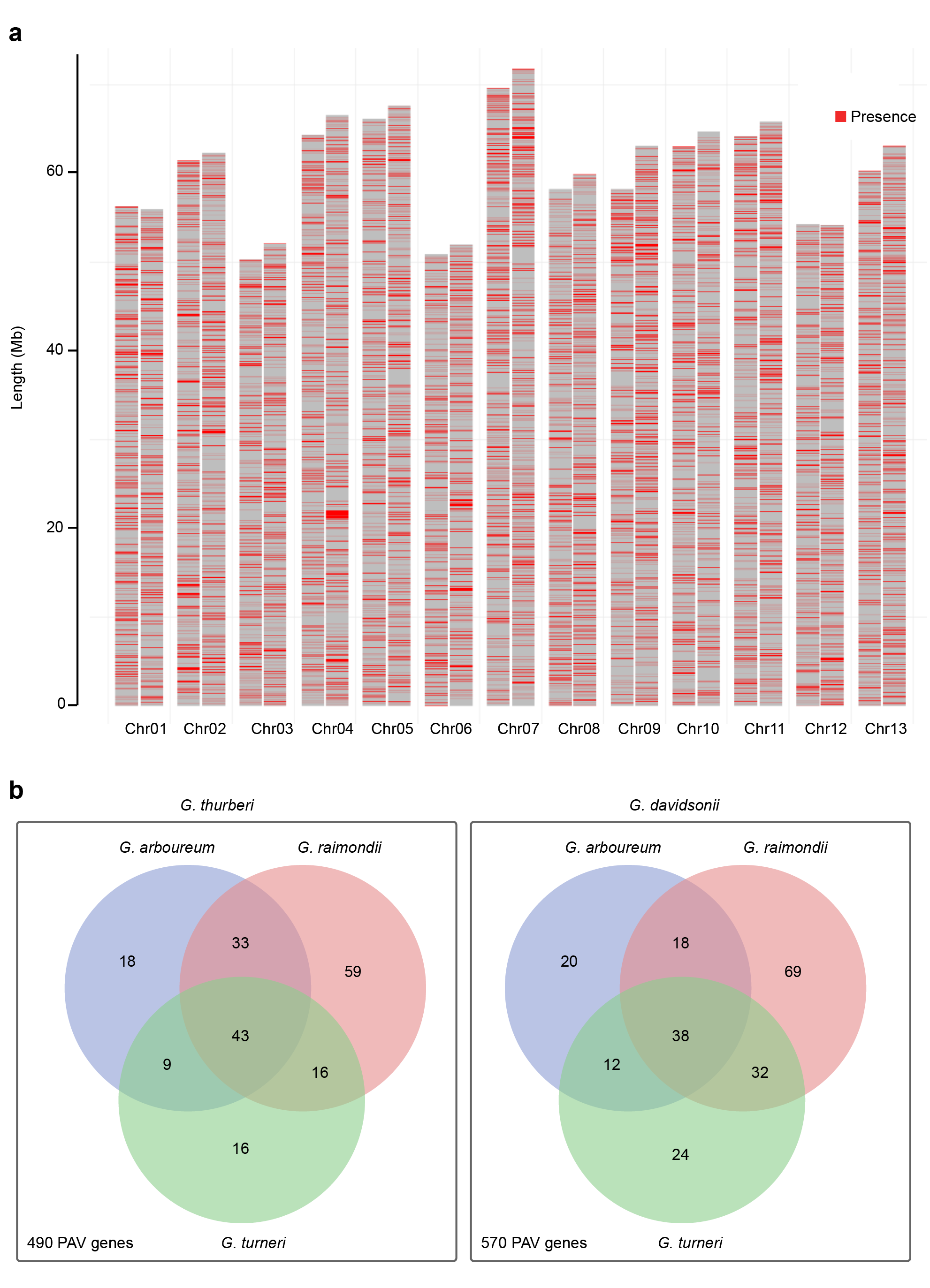


Figure S8 PAV analysis in *G. thurberi* and *G. davidsonii*. a，Present segments in each chromosome. The red boxes indicate the insertion segments. b，The number of PAV genes that have orthologs in *G.arboreum*, *G.raimondii* and *G. turneri*.


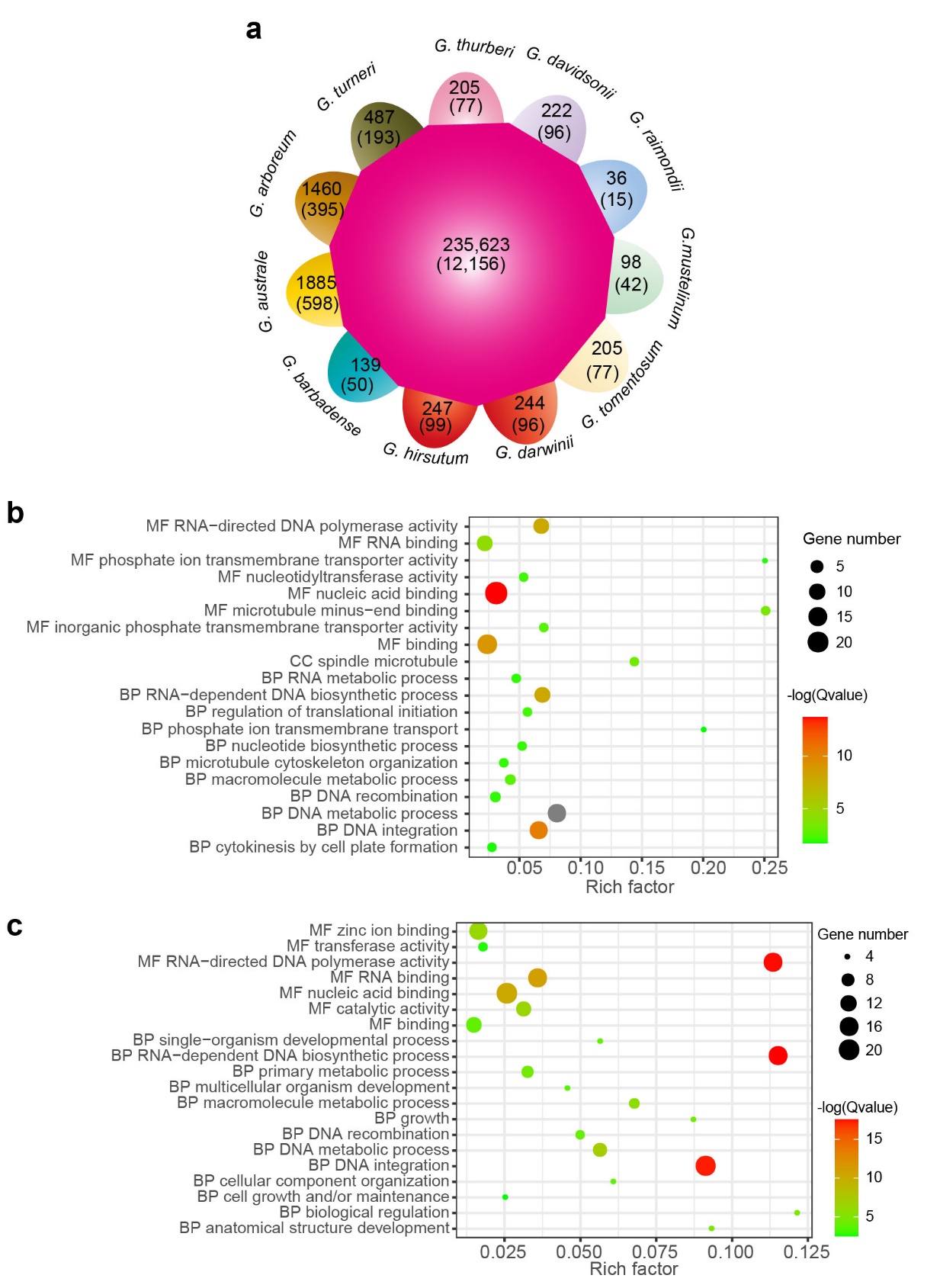


Figure S9 Gene family expansion analysis between the new assemblies with the previously reported close relative species. a，Unique genes in eleven examined species. The D_t_ subgenomes of the five allotetraploid cotton species were used. The digitals above the parentheses are the gene number, and these within the parentheses are the orthologous groups. b. GO enrichment analysis of unique genes from *G. thurberi*. c, GO enrichment analysis of unique genes from *G. davidsonii*. The MF, BP, CC before the GO terms are short for molecular function, biological process and cellular component.


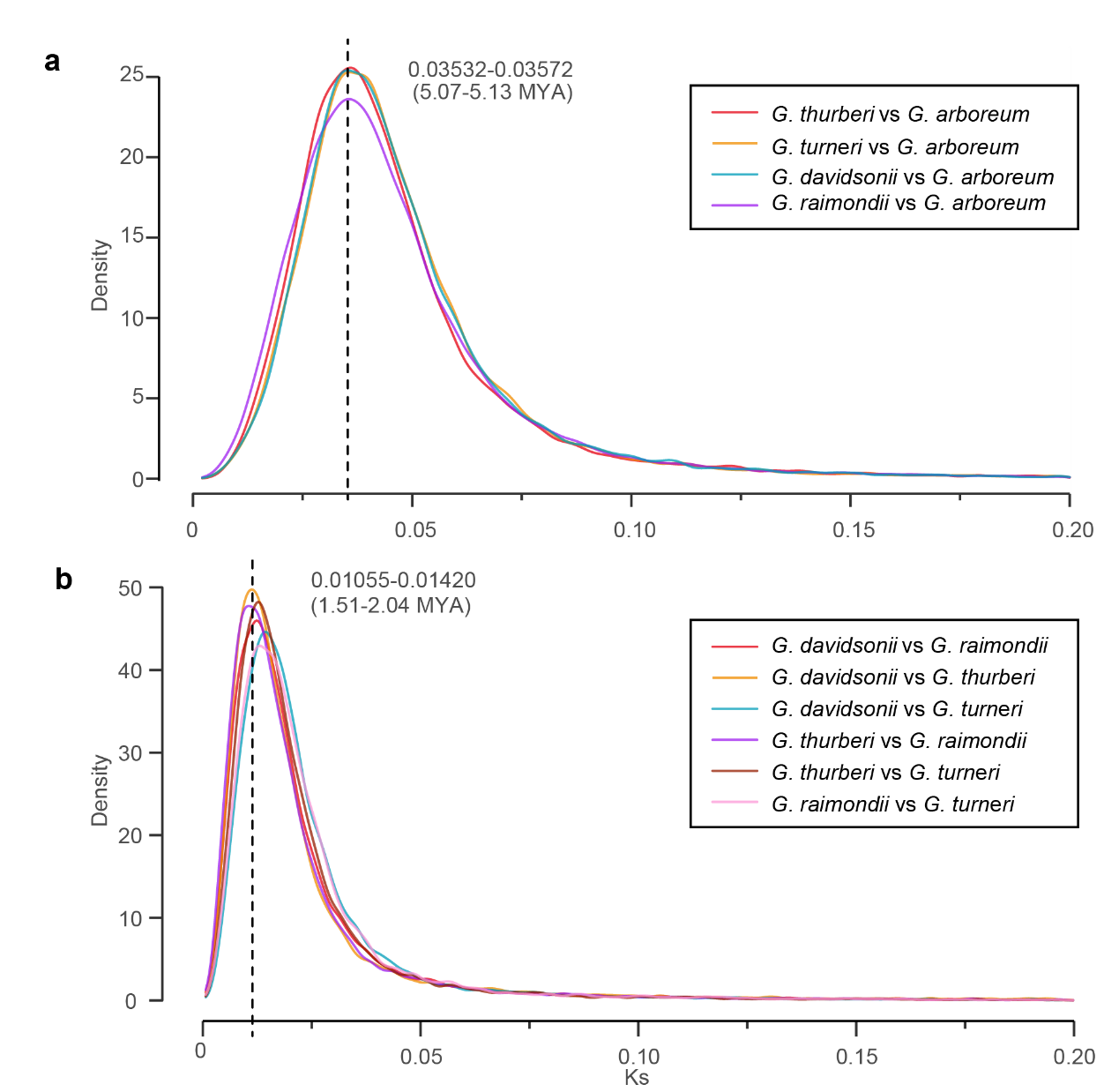


Figure S10 Evaluation of divergence time among the close relative species*.* a，Ks distribution of *G. arboreum* with the four D-genome species (*G. thurberi*, *G. davidsonii*, *G. raimondii* and *G. turneri*). b，Ks distribution within the D-genome species.


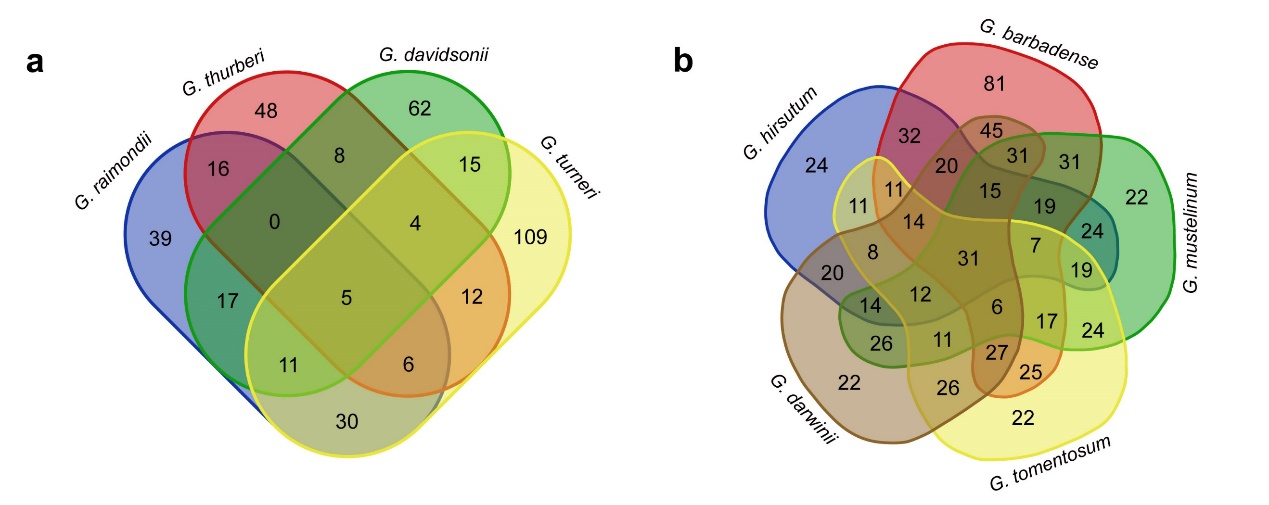


Figure S11 Comparison of gene family have experienced expansion or contraction. a，b, Venn diagram of the gene families which have experienced expansion or contraction in the four diploid D genomes (c) or in the five allotetraploid D subgenomes (d).


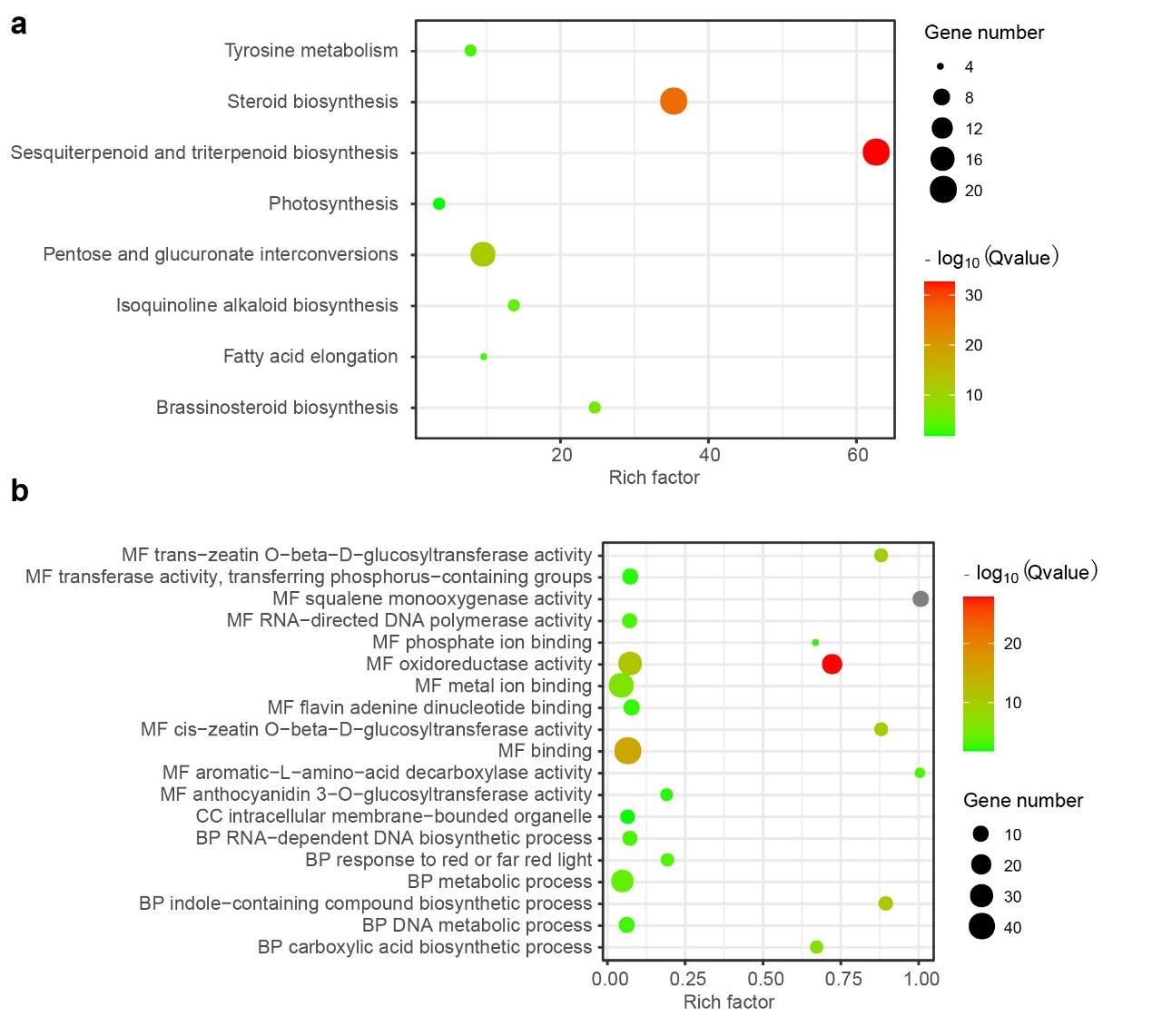


Figure S12 KEGG and GO enrichment analysis of the genes from *G. thurberi* experienced expansion. a, Pathways enrichment in KEGG analysis. b, GO terms enrichment in GO analysis.


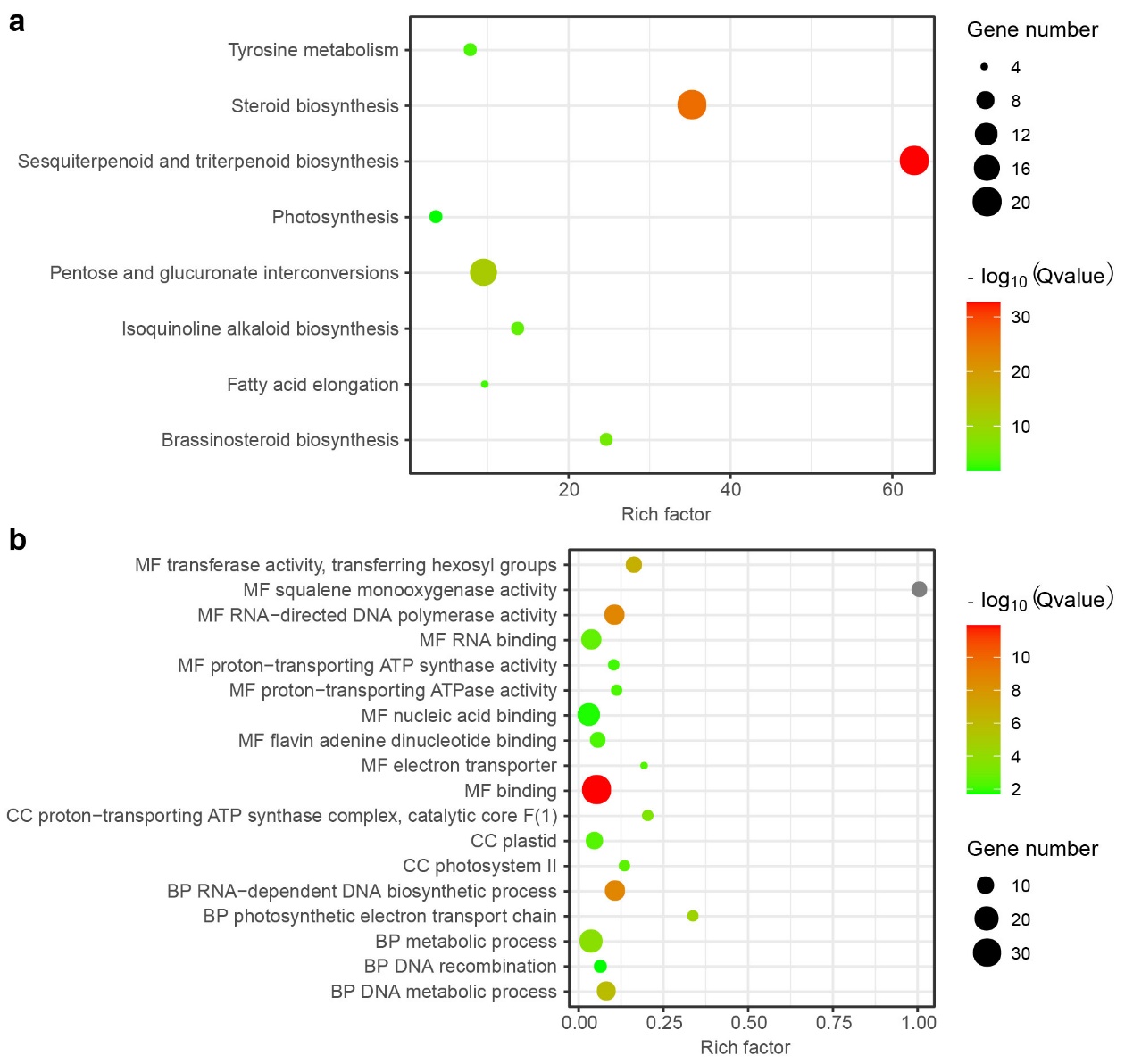


Figure S13 KEGG and GO enrichment analysis of the genes from *G. davidsonii* experienced expansion. a，Pathways enrichment in KEGG analysis. b, GO terms enrichment in GO analysis.


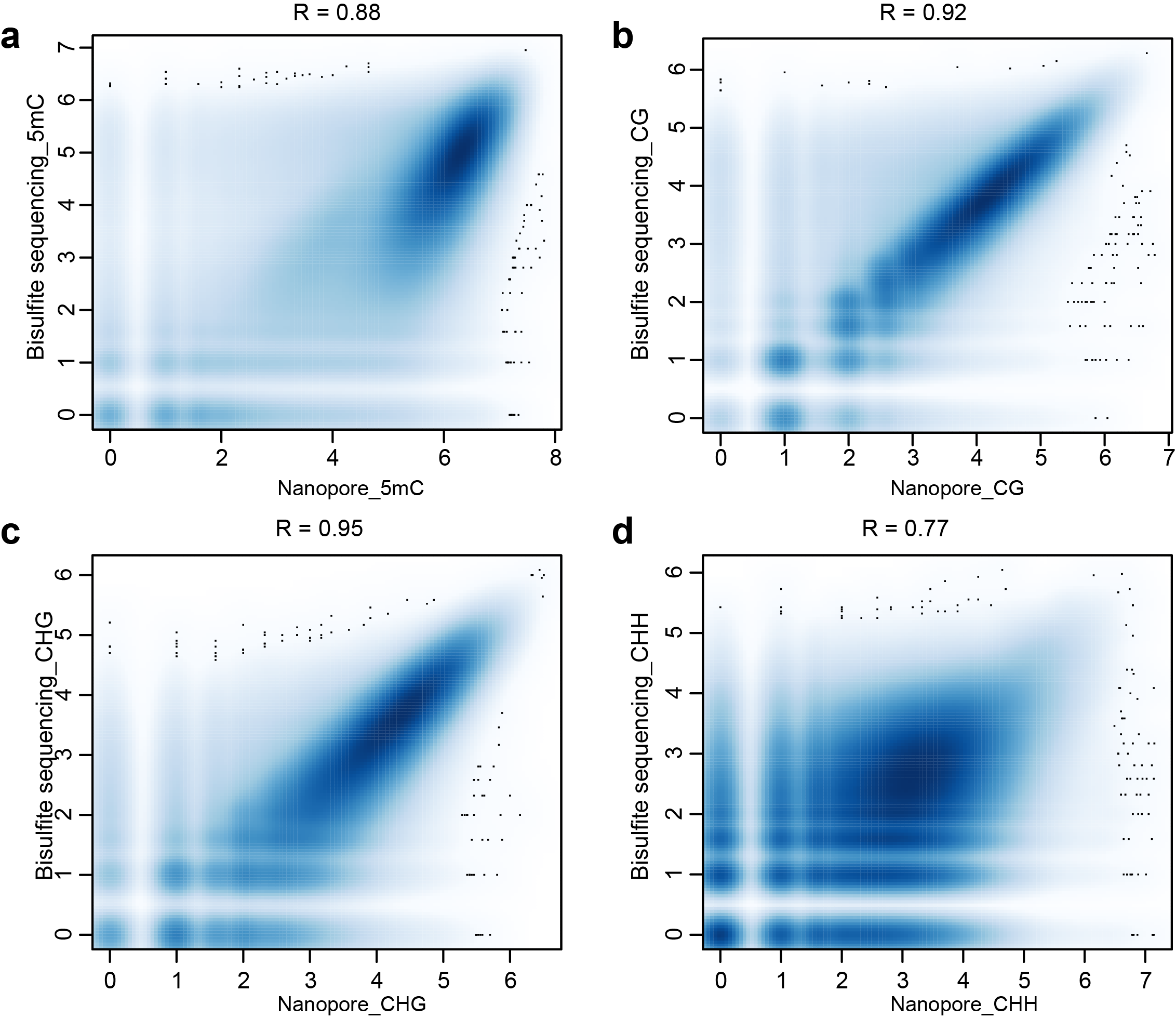


Figure S14 Comparison of 5mC as acquired with ONT Nanopore technology and with bisulfite sequence (Bs-seq) technology. a，Correlation of 5mC (CG, CHG, and CHH). b，Correlation of CG. c，Correlation of CHG. d，Correlation of CHH.

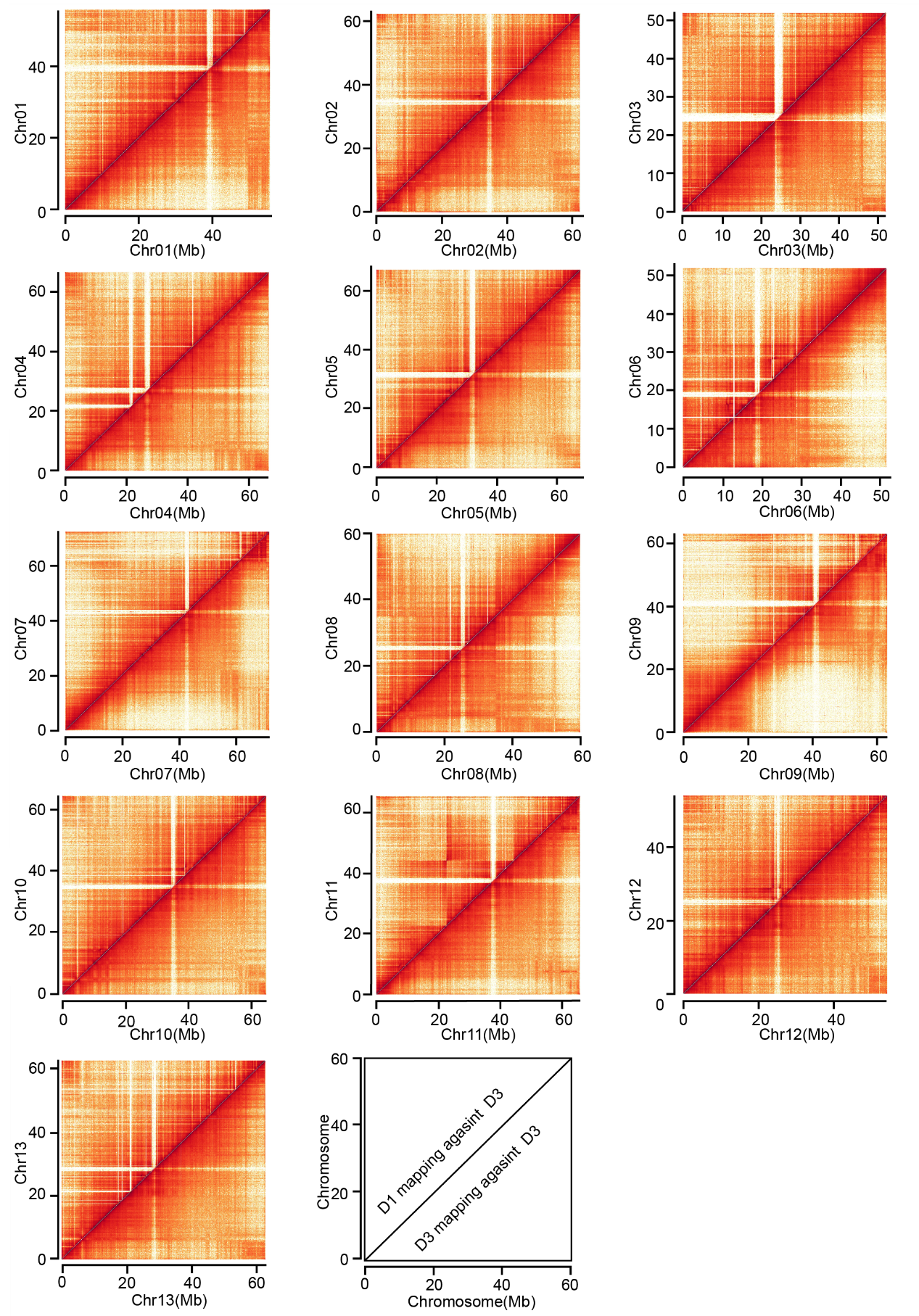


Figure S15 Hi-C contact data mapping against the D_3_ genome. The upper panel is the D_1_ mapping against the *G. davidsonii* genome. The lower panel is the D_3_ mapping against the D_3_ genome.


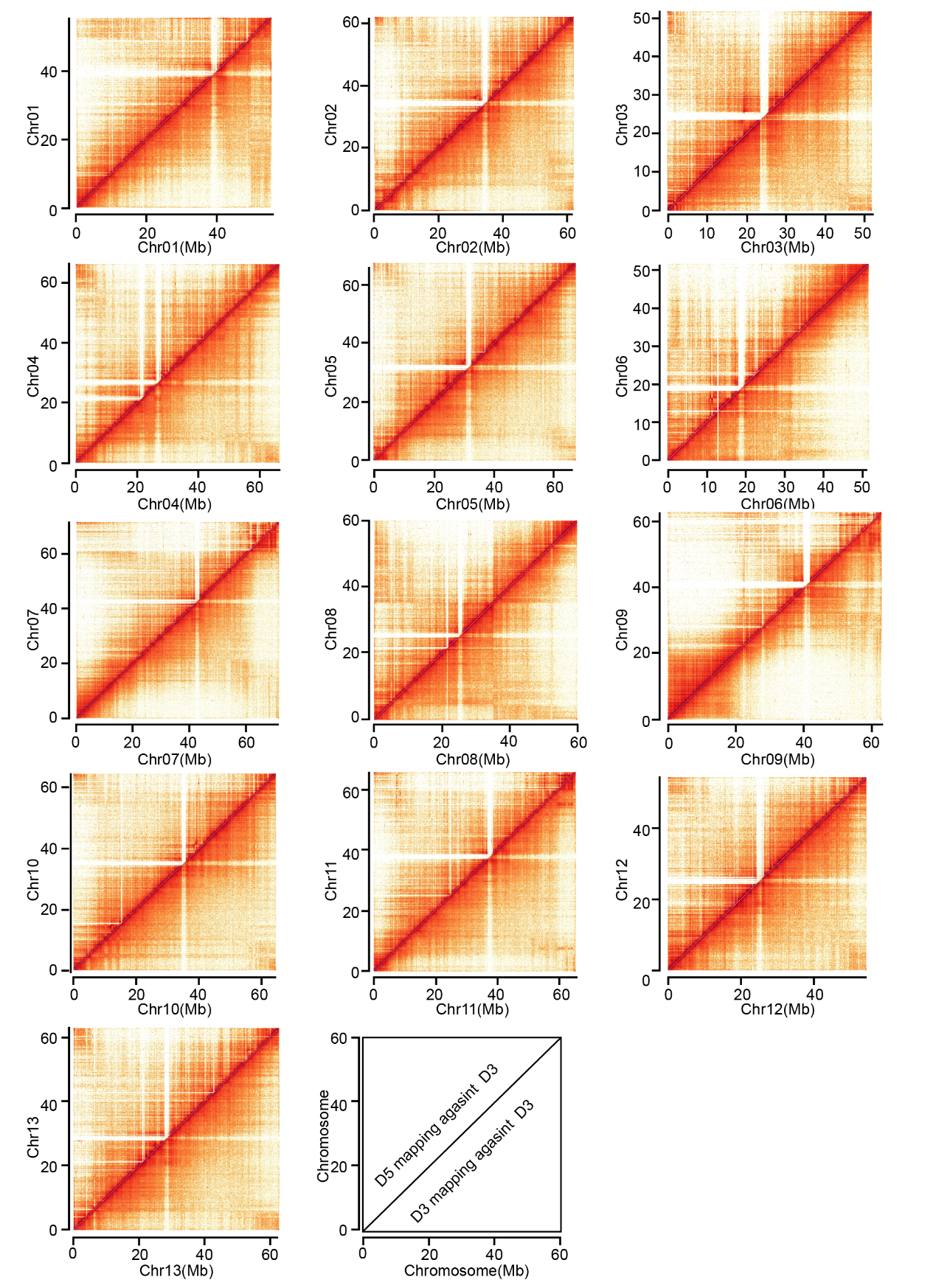


Figure S16 Hi-C contact data mapping against the D_3_ genome. The upper panel is the D_5_ mapping against the D_3_ genome. The lower panel is the D_3_ mapping against the D_3_ genome.


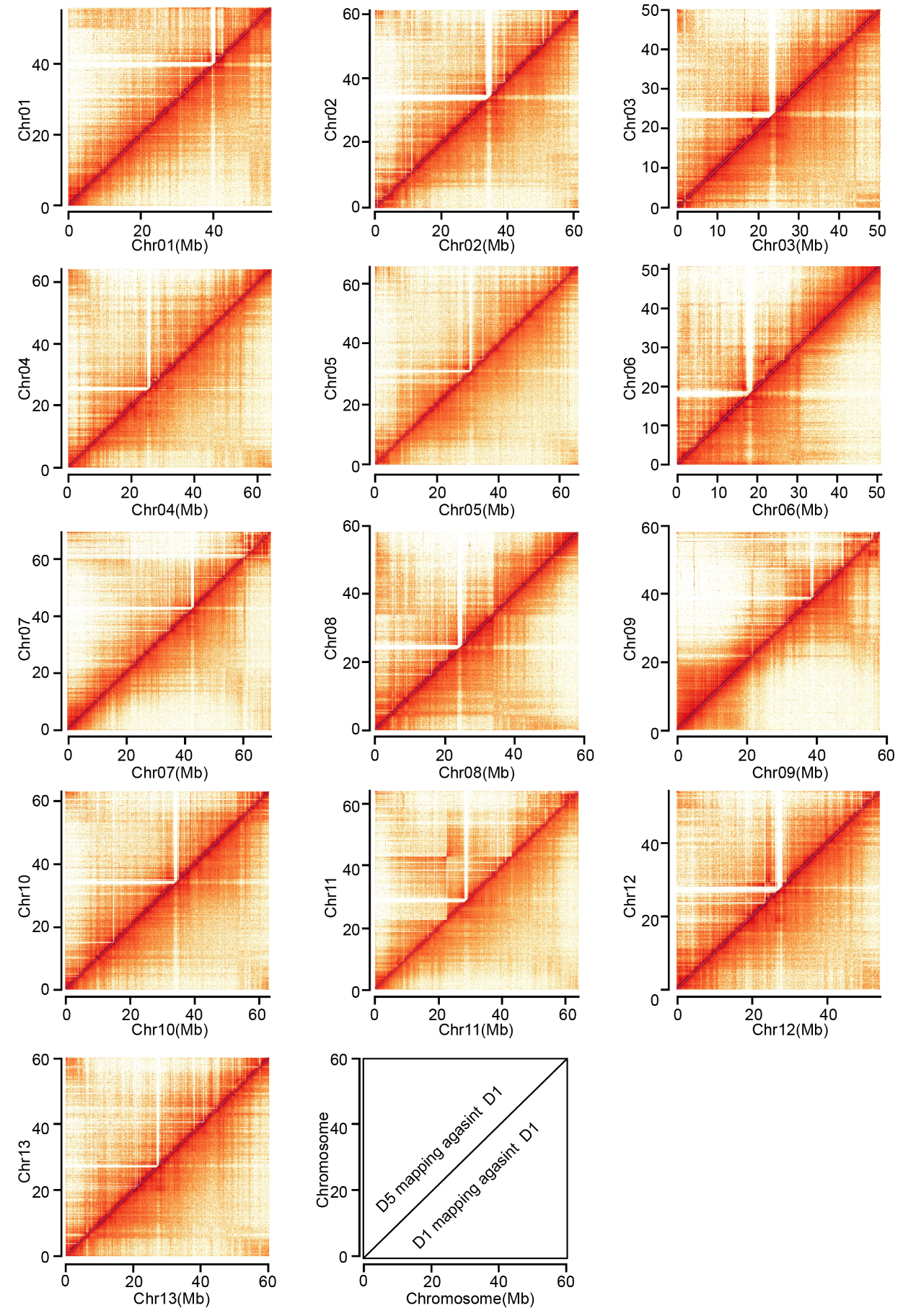


Figure S17 Hi-C contact data mapping against the D_1_ genome. The upper panel is the D_5_ mapping against the D_3_ genome. The lower panel is the D_1_ mapping against the D_1_ genome.


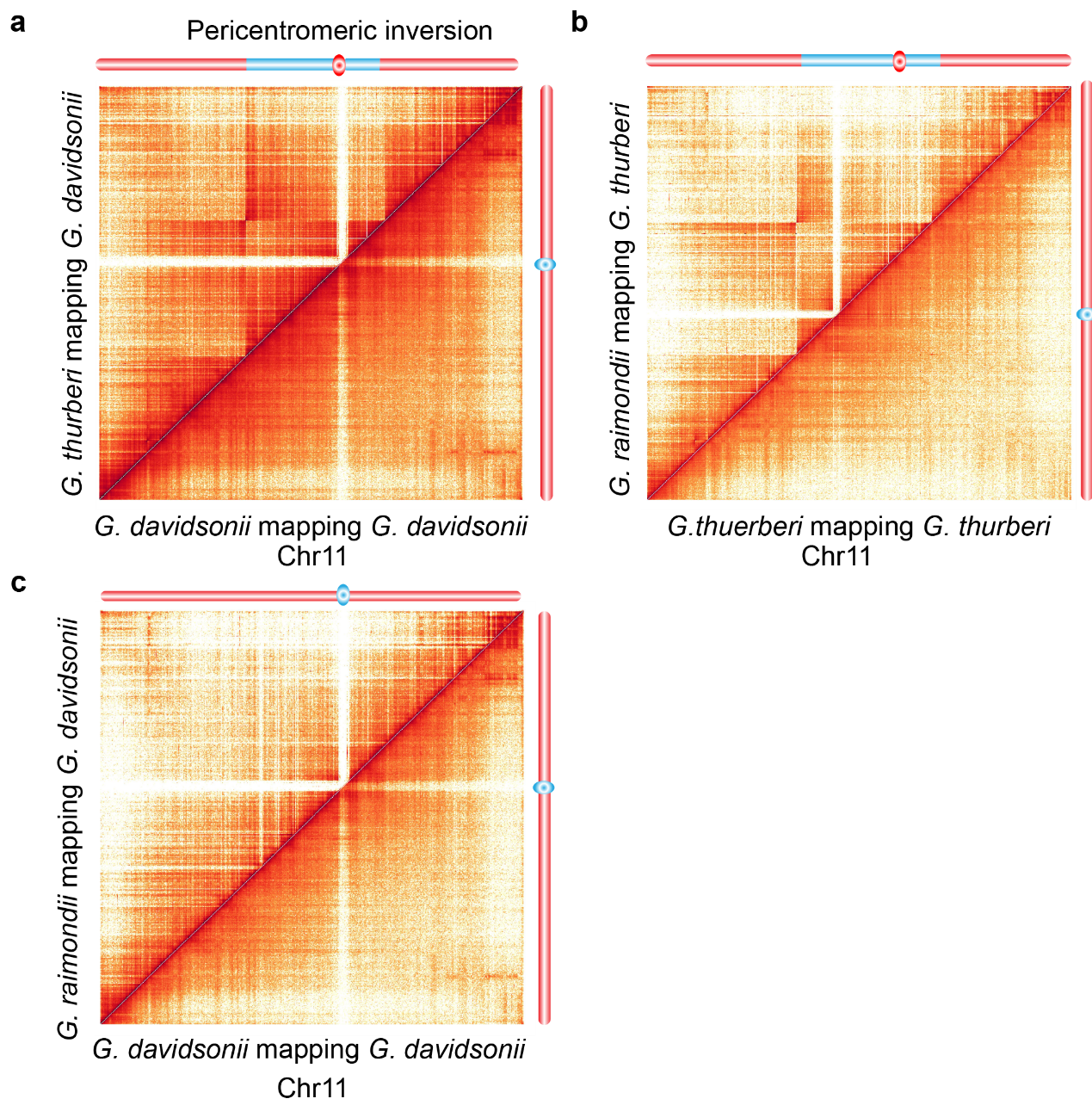


Figure S18 The rewriting of chromatin interactions in the inverted regions. a，Thu upper panel is *G. thurberi* Hi-C contact data mapping against the *G. davidsonii* Chr11 genome. The chromatin interaction showed a reversing pattern in the blue marking inverted regions. The lower panel is *G. davidsonii* Hi-C contact data mapping against the *G. davidsonii* Chr11 genome. The chromatin interaction frequency is decreasing as the distance between two loci is increasing. b，The upper panel is *G. raimondii* Hi-C contact data mapping against the *G. thurberi* Chr11 genome. The chromatin interaction showed a reversing pattern in the blue marking inverted regions. The lower panel is *G. thurberi* Hi-C contact data mapping against the *G. thurberi* Chr11 genome. c，Thu upper panel is *G. raimondii* Hi-C contact data mapping against the *G. davidsonii* Chr11 genome. The lower panel is *G. davidsonii* Hi-C contact data mapping against the *G. thurberi* Chr11 genome.


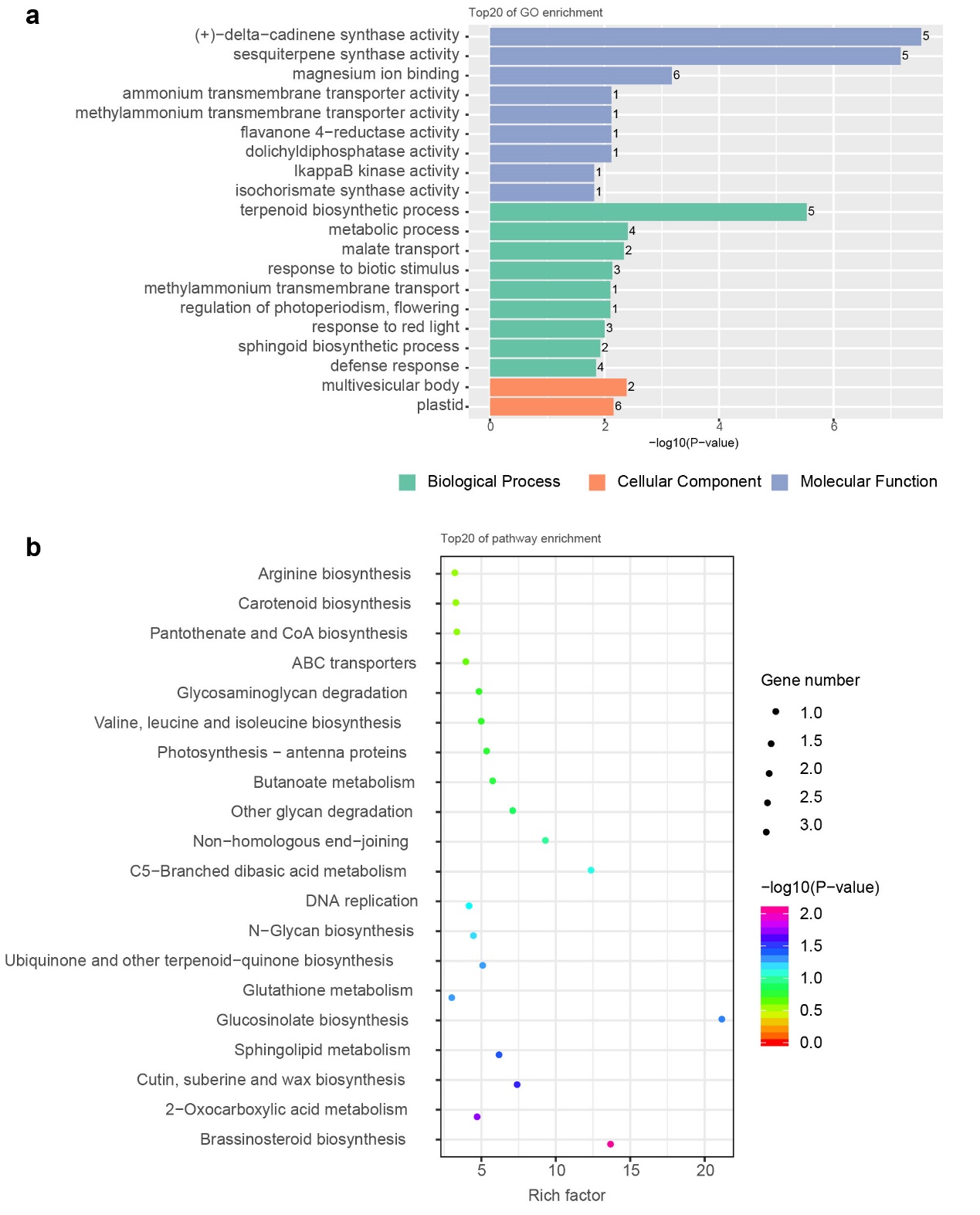


Figure S19 GO and KEGG enrichment analysis of differential expressed genes with differential P-D interactions. a, GO terms enrichment in GO analysis. a，Pathways enrichment in KEGG analysis.


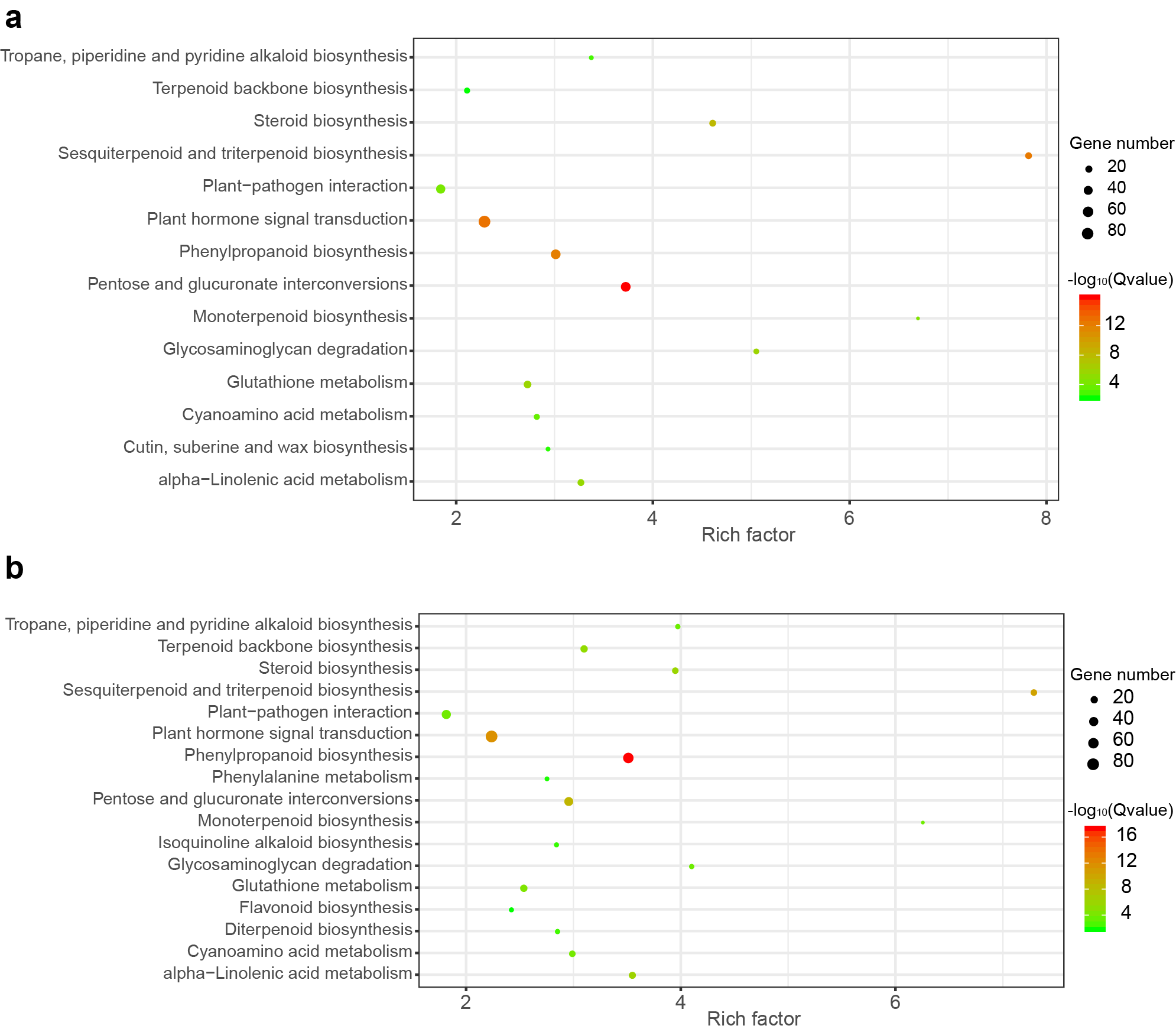


Figure S20 KEGG enrichment of tandem duplication genes in *G. thurberi* (a) and *G. davidsonii* (b)*.*

*
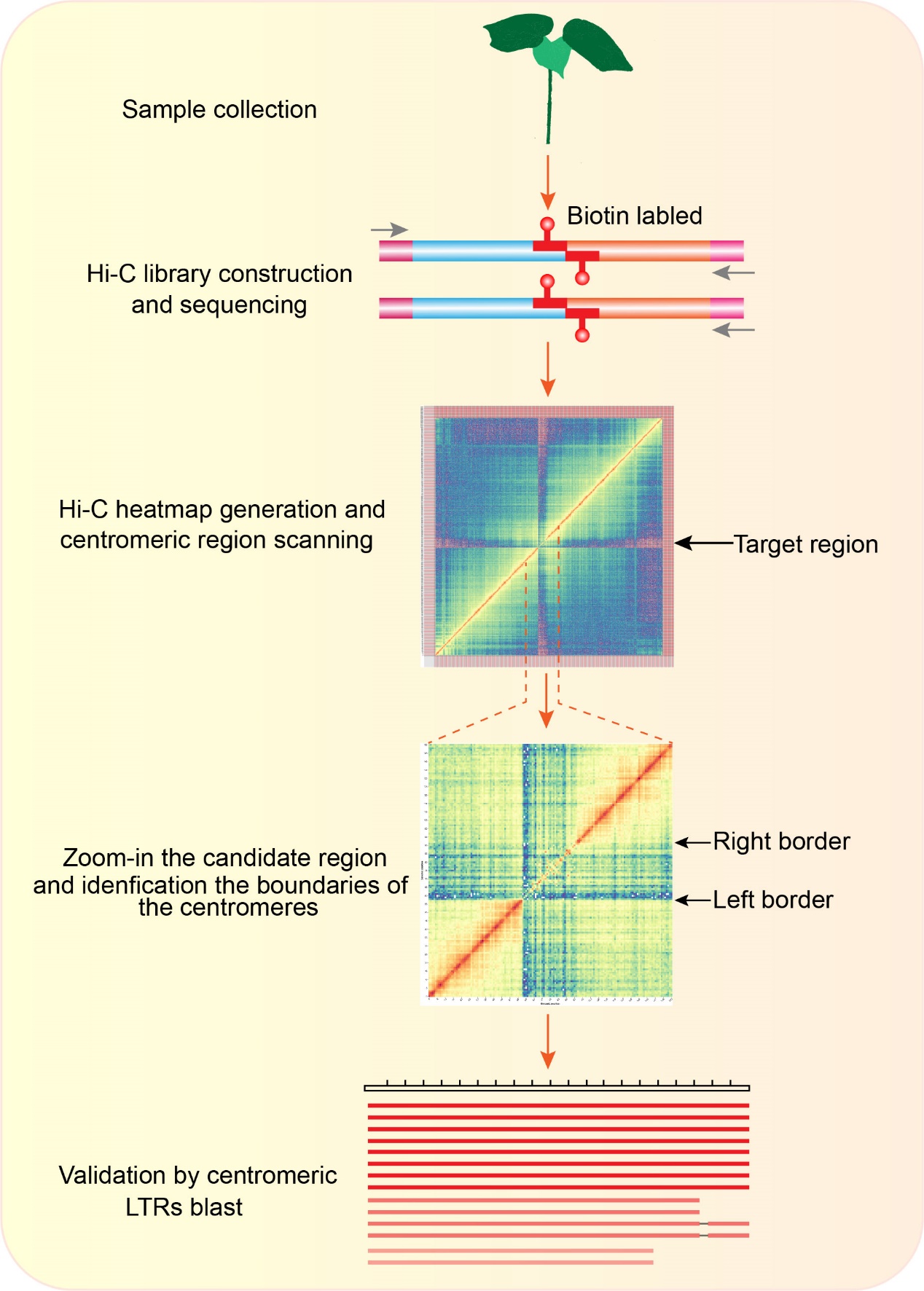
*

Figure S21 Overview of the identification of centromeres by Hi-C heatmap.

*
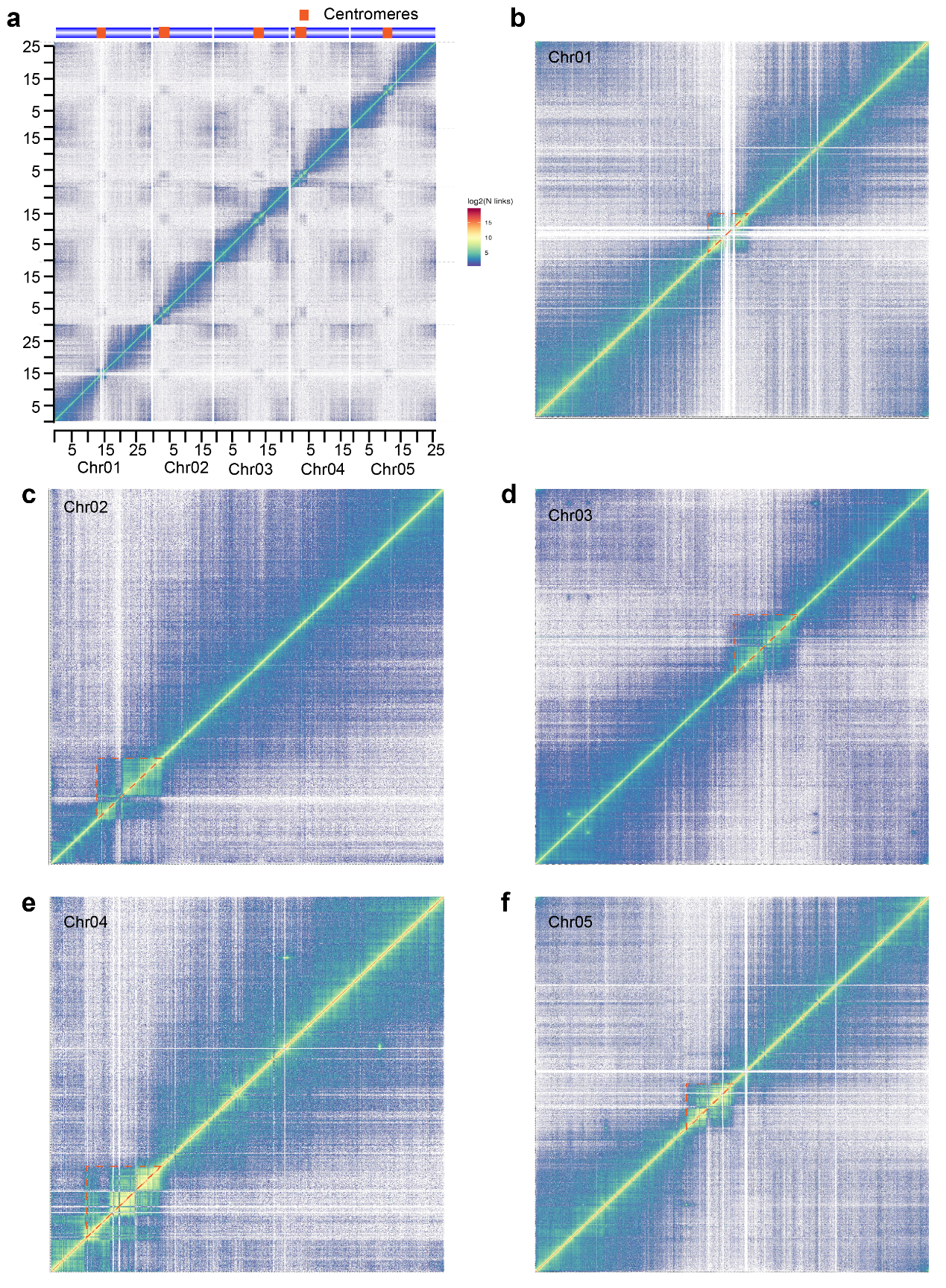
*

Figure S22 Identification of centromeres using Hi-C contact heatmap in *Arabidopsis thaliana*. a, Hi-C heatmap for the whole genome. b-f，The centromeres in Chr01 to Chr05. The triangles indicate the centromeres in the heatmaps. The data used here was downloaded from NCBI SRA under accession SRR1029605.

*
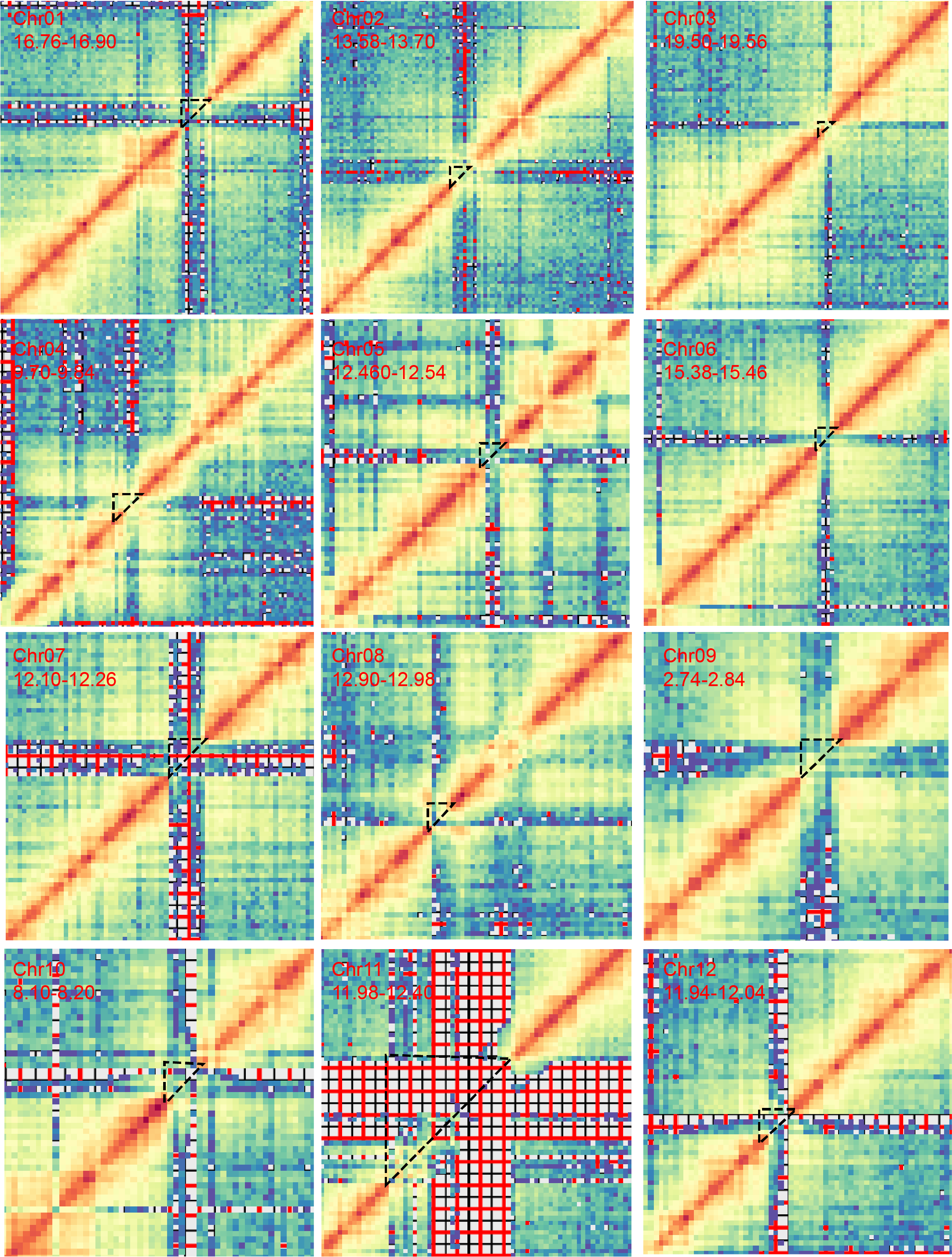
*

Figure S23 Identification of centromeres using a 20-Kb resolution Hi-C contact heatmap for *Oryza sativa* Nipponbare. The triangles indicate the centromeres in the heatmaps. The data used here were downloaded from NCBI SRA (accession SRR5046932). The centromeres have been present in the upper left corner of each heatmap.


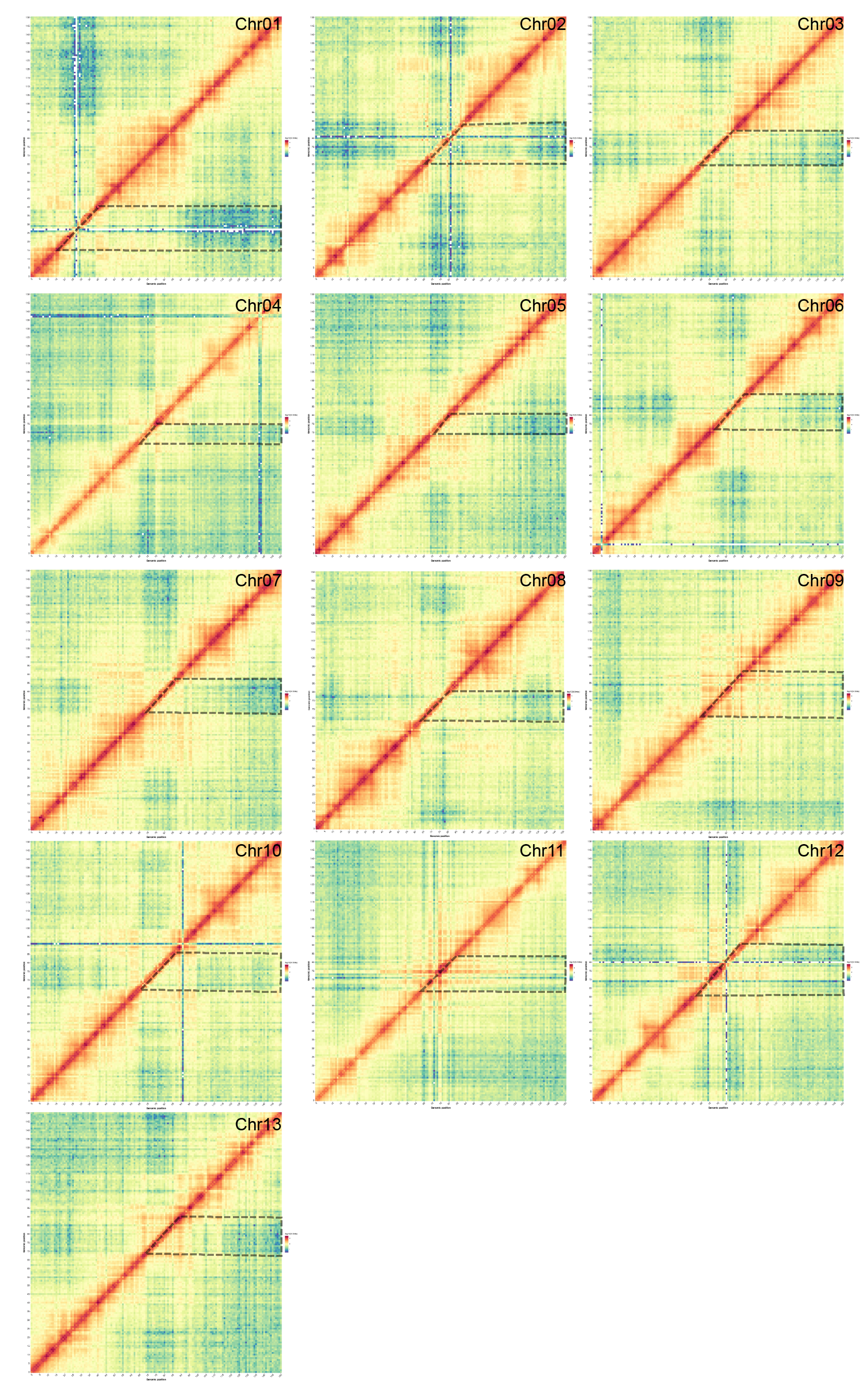


Figure S24 Hi-C contact heatmap of centromeric regions in *G. thurberi*. The dash line trapezoid indicates the centromere.


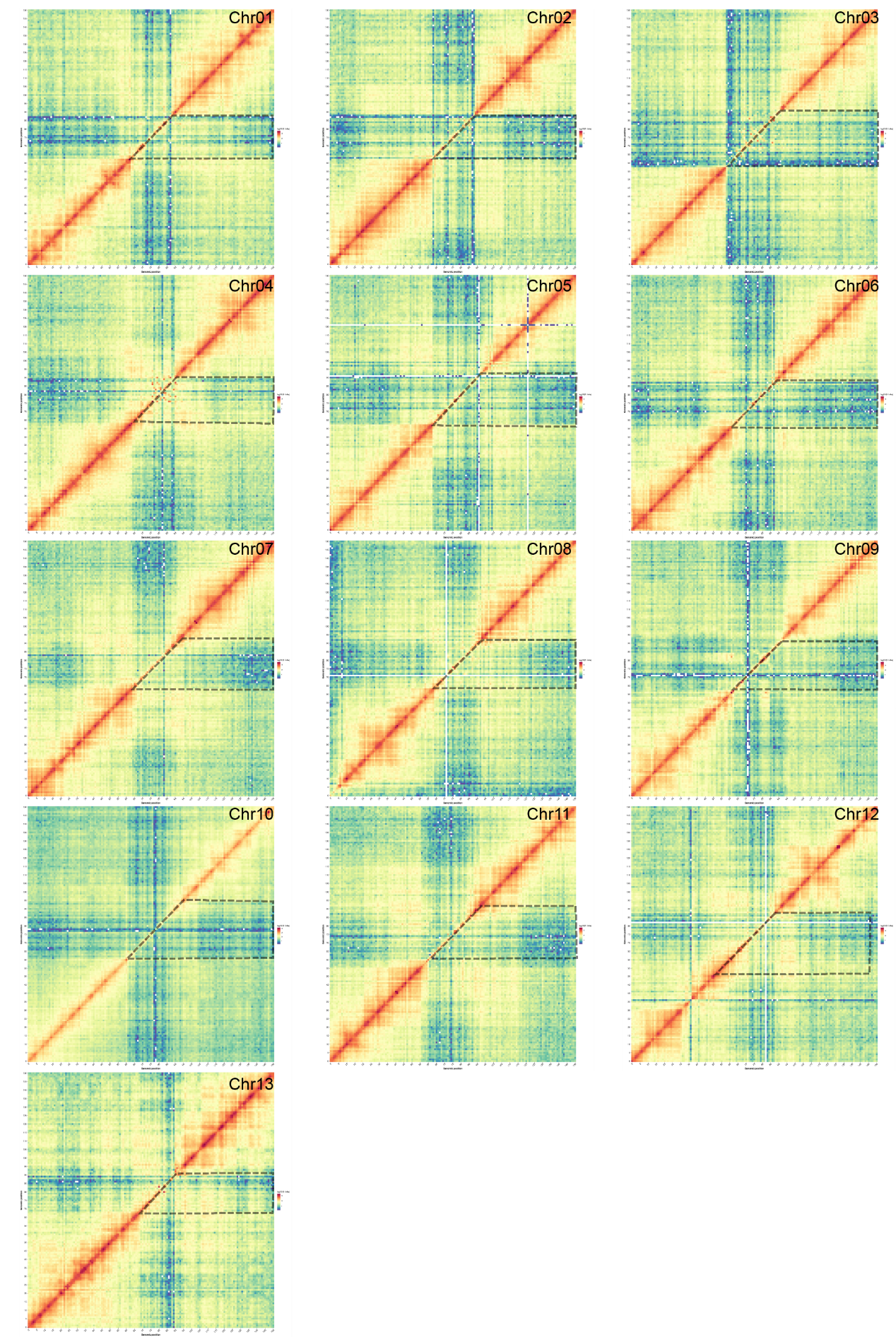
 Figure S25 Hi-C contact heatmap of centromeric regions in *G. davidsonii*. The dash line trapezoid indicates the centromere.


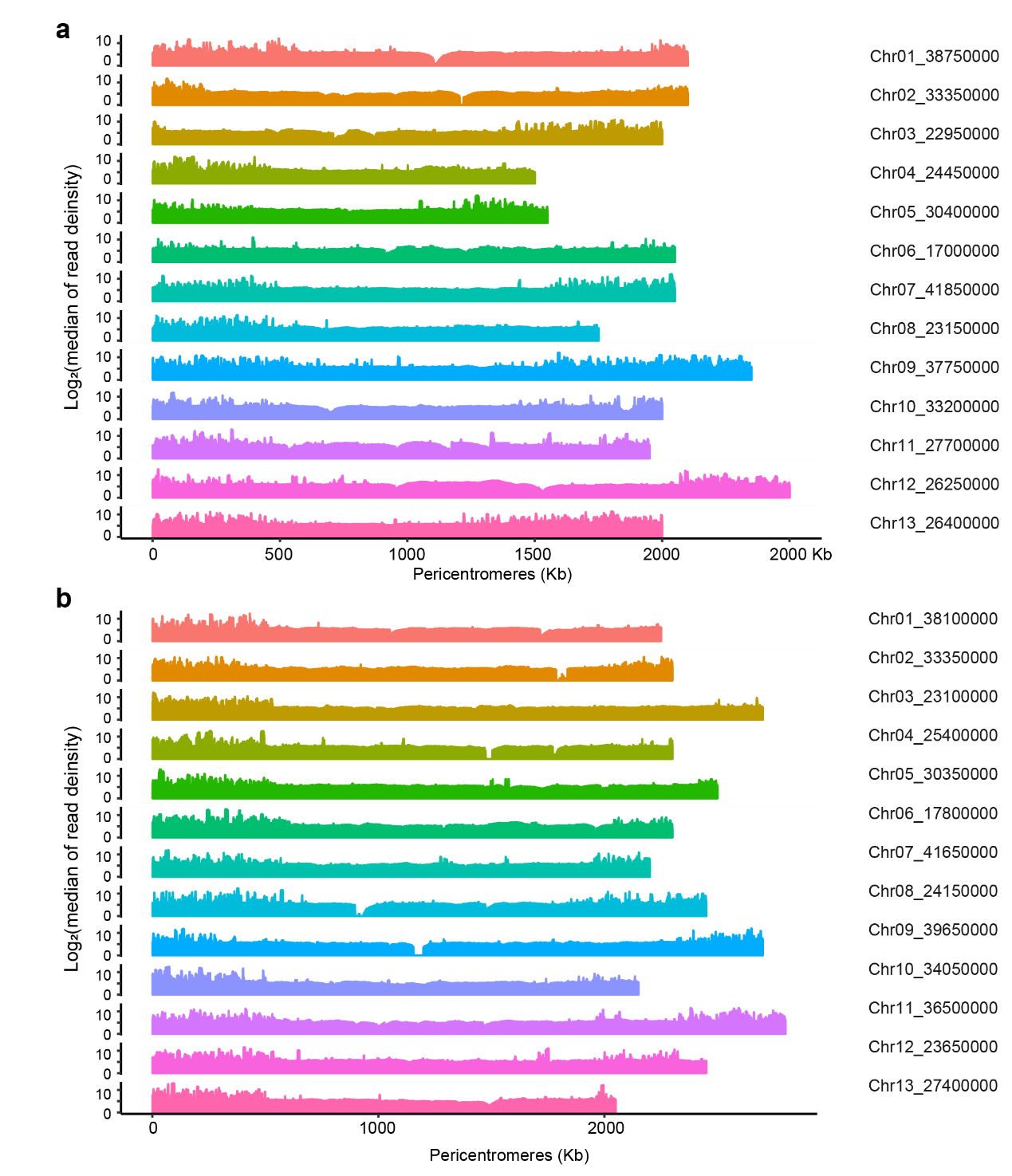


Figure S26 Nanopore reads coverage in centromeric regions*.* a，The reads density in *G. thurberi*. b, The reads density in *G. davidsonii*. The upstream and downstream 500 kb of the centromeres are also shown in the figure. The reads coverage was calculated in 1000 bp window, and the log2 value of median of the read coverages was used in the drawing.


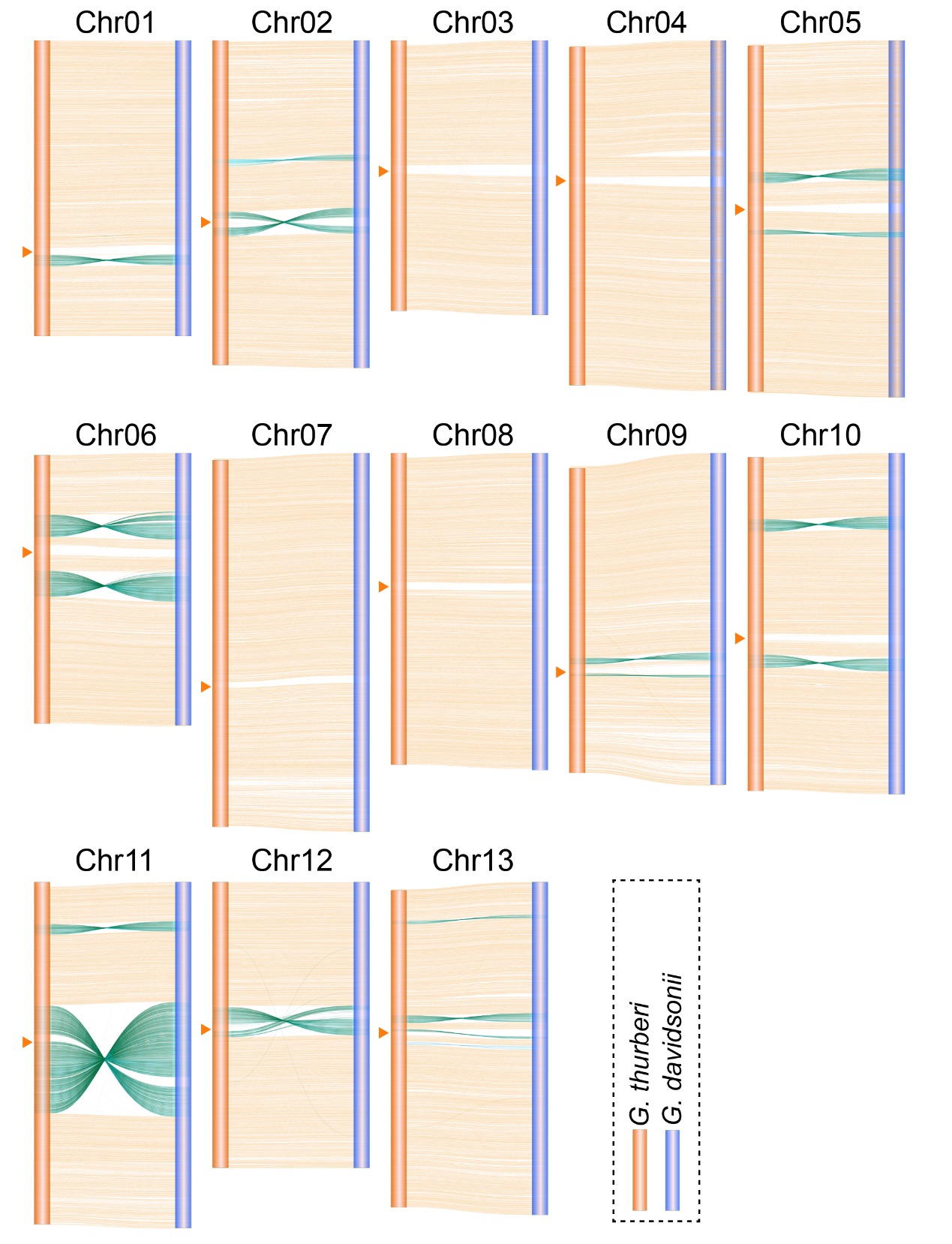


Figure S27 Genomic comparison between *G. thurberi* and *G. davidsonii* shows that the regions indicated by the triangles are highly divergent.


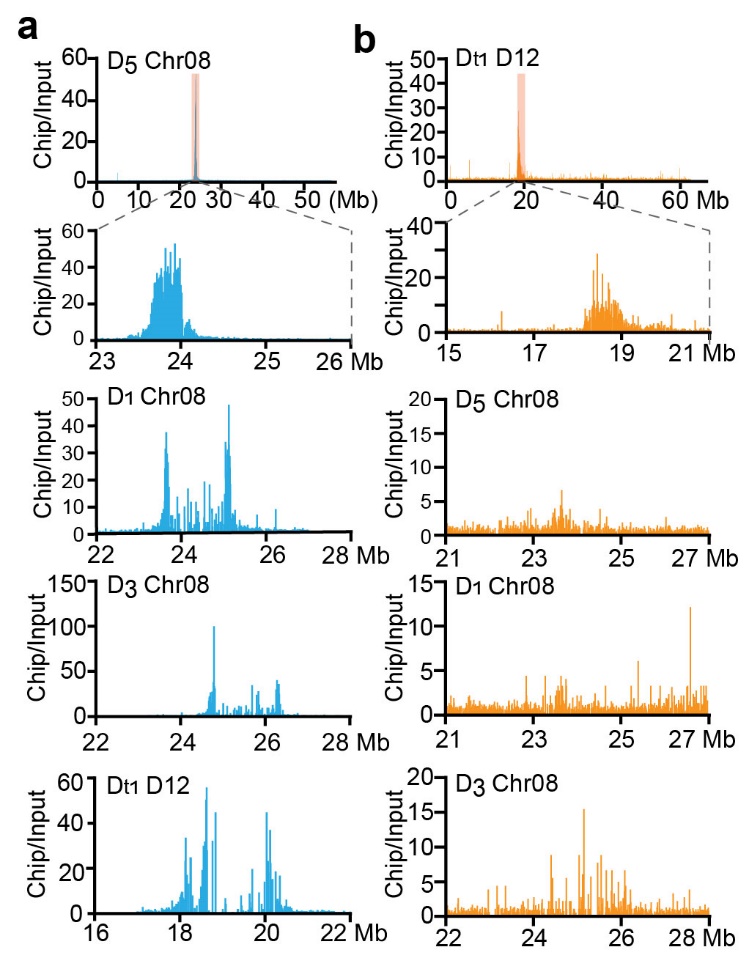


Figure S28 Reads mapping of CENH3 ChIP-Seq data. a，b, The left and right panels are *G. raimondii* and *G. hirsutum* data mapped against *G. raimondii*, *G. thuruberi*, *G. davidsonii*, and *G. hirsutum*. The read count was calculated for 10 Kb windows.


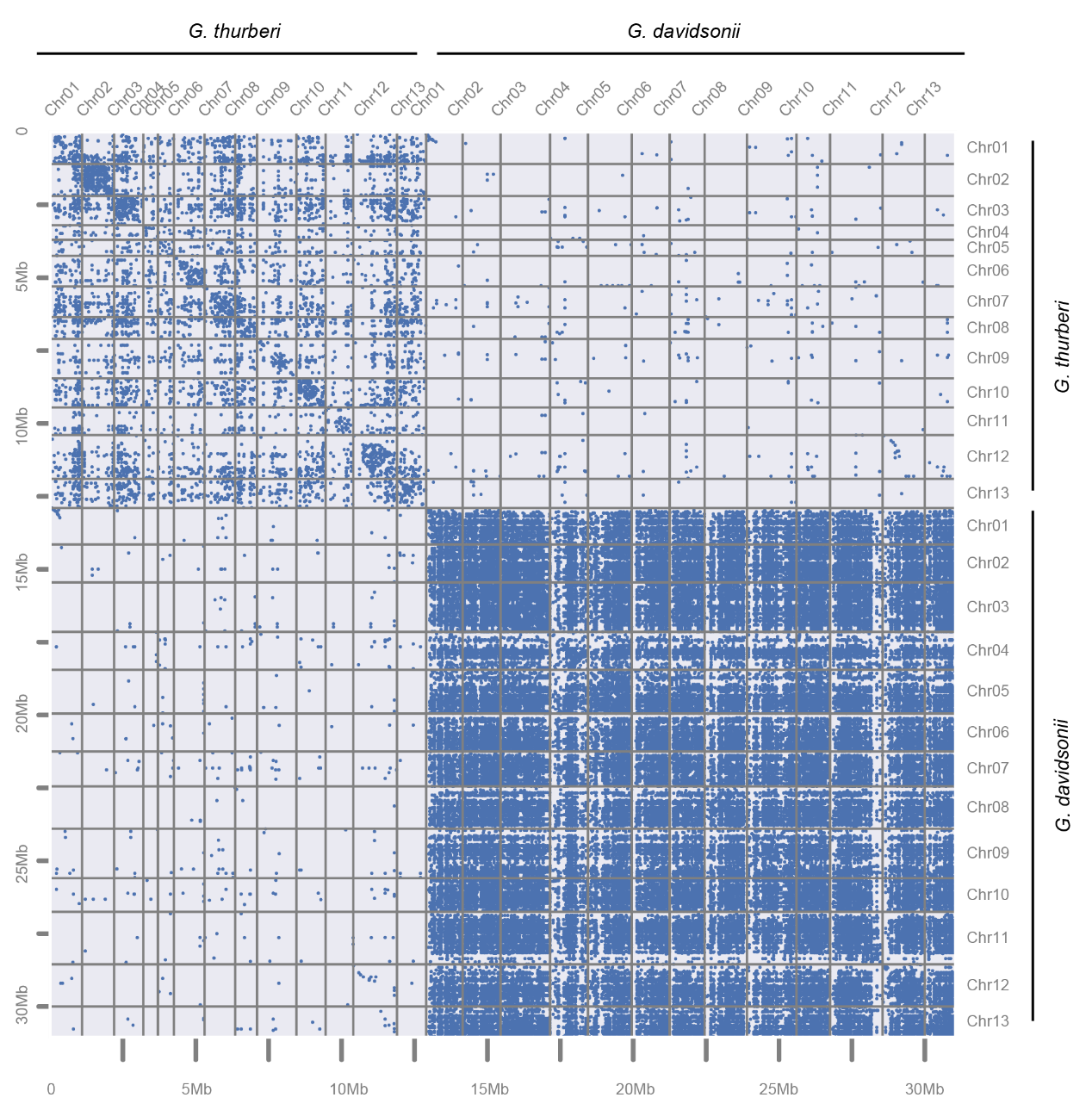


Figure S29 Dotplot shows that segmental duplication arises within the centromeres after speciation of G. *thurberi* and *G. davidsonii*. The Blastn was used to do all_vs_all genomic sequences alignments with evalue of 1e-5. Then blast results with 95 % identity and 2000 bp aligned length for *G. thurberi* are present in the dotplot.


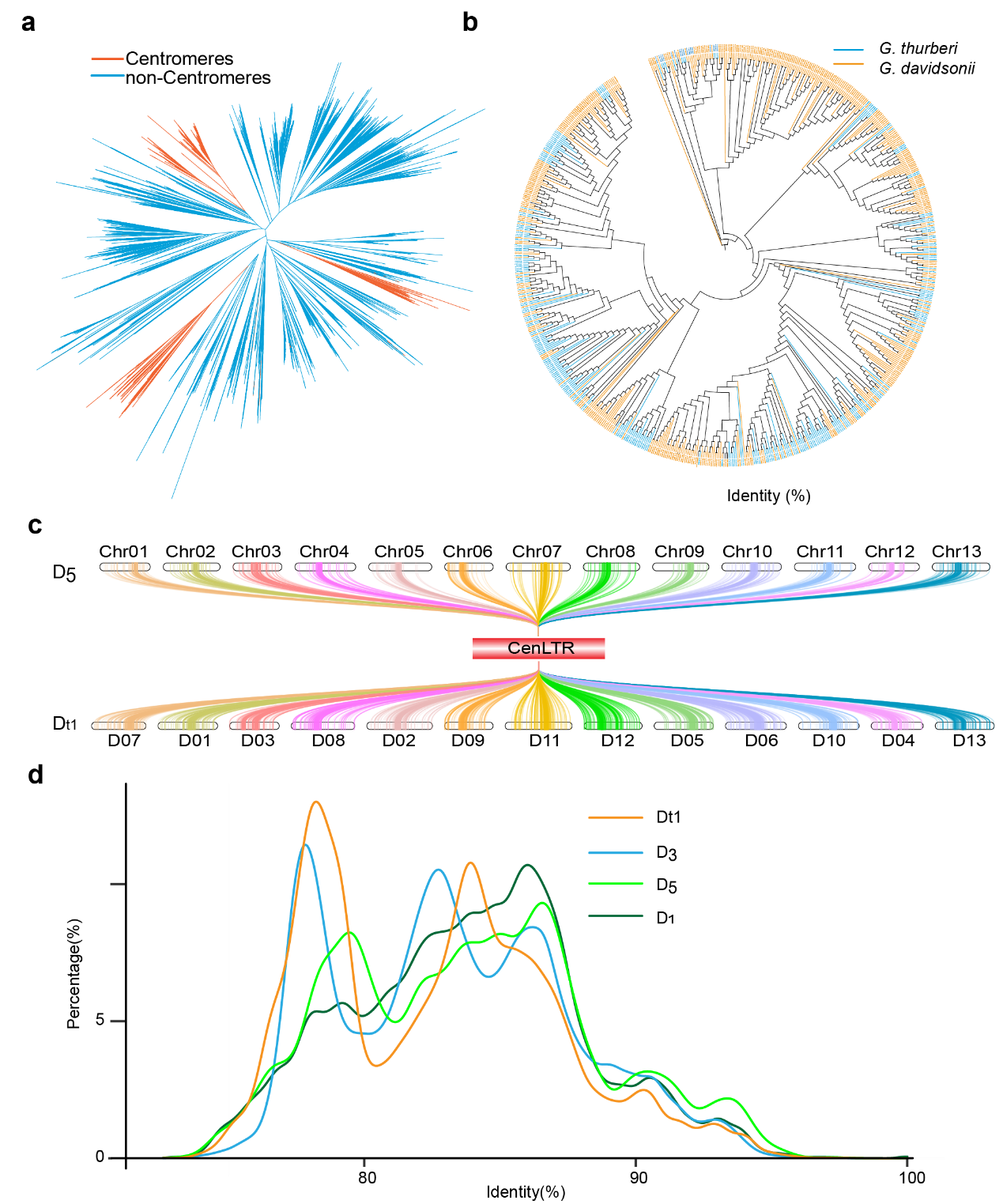


Figure S30 Centromeres are composed of the LTRs. a，Phylogenetic cluster shows that centromere LTRs can divided into 3 subclades. c, Phylogenetic tree analysis based on 5’ LTR from the centromeres shows a species-specific cluster pattern. Th blue color indicates LTRs from D_1_ and orange color indicates the LTRs from D_3_. c，The distribution of CenLTR hits in D_5_ and D_t1_ genomes. d, Sequence identity with the CenLTR consensus sequence in D_1_, D_3_, D_5_ and D_t1_ genomes.


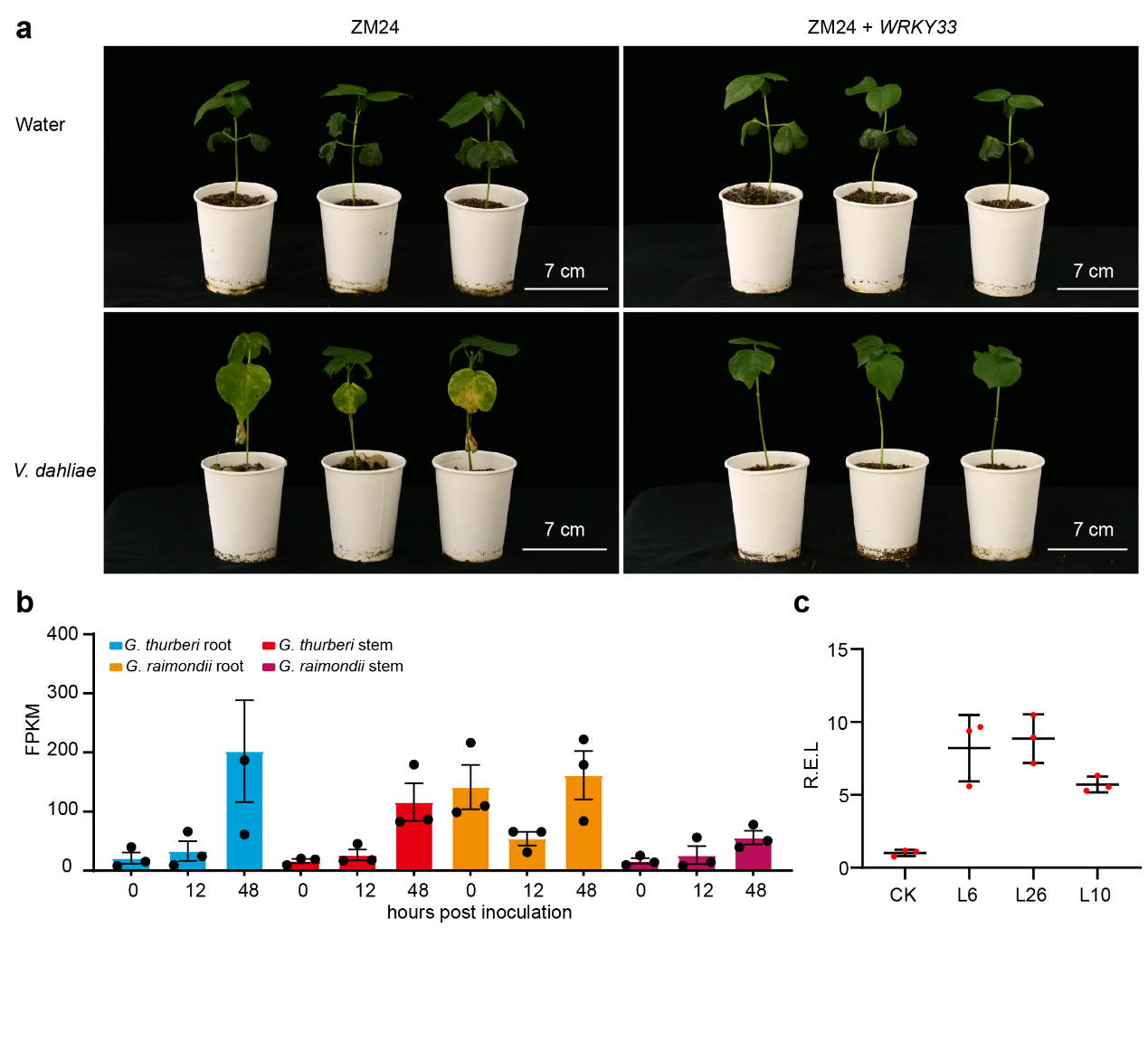


Figure S31 Overexpression WRKY33 gene increasing the ZM24 tolerance to *Verticillium dahliae*. a，The overexpression cotton enhancing tolerance to *Verticillium dahliae*. Phenotypic photos were taken at 14 days after inoculation. b, Gene expression pattern under *Verticillium dahliae* treatment in *G. thurberi* and *G. raimondii*. c, Relative expression level (R.E.L.) of WRKY33 in the ZM24 (CK) and overexpression lines (L6, L26 and L10). The 2^-ΔΔCT^ method was used to normalized the R.E.L (relative expression level).
